# Palladium-Catalyzed Intermolecular Tandem Difunctional Carbonylation of 1,3-Enynes: Synthesis of Fluoroalkylated Butenolides

**DOI:** 10.1021/acs.orglett.5c03004

**Published:** 2025-07-25

**Authors:** Chang-Sheng Kuai, Ru-Han A, Zhi-Peng Bao, Xiao-Feng Wu

**Affiliations:** [a] Dalian National Laboratory for Clean Energy, Dalian Institute of Chemical Physics, Chinese Academy of Sciences, Dalian 116023, China; [b] University of Chinese Academy of Sciences, Beijing 100049, China; [c] Leibniz-Institut für Katalyse e. V., Albert-Einstein-Straβe 29a, 18059 Rostock, Germany

## Abstract

A palladium-catalyzed tandem difunctional carbonylation of 1,3-enynes with fluoroalkyl halides and water has been developed, enabling efficient one-step access to fluoroalkylated butenolides. The method exhibits a broad substrate scope, high chemo- and regioselectivity, and good functional group tolerance. Mechanistic studies support a Pd/Cu-cooperative pathway involving radical addition and carbonylative cyclization.

## Graphical abstract

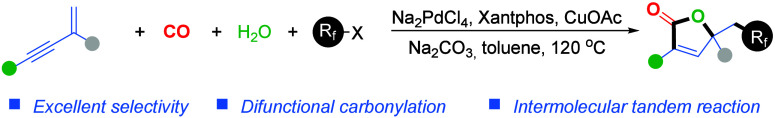

The butenolide scaffold, featuring a five-membered α,β-unsaturated γ-lactone ring, constitutes a privileged structural motif that is broadly embedded in a wide array of biologically active natural products and pharmaceutical agents.^1^ This structural unit is associated with diverse biological functions. For instance, vitamin C (ascorbic acid) is renowned for its potent antioxidant and antimicrobial properties; avenolide, a bacterial signaling molecule, exhibits notable antibiotic activity; norustroporin displays both antiviral and antifungal effects; and the anti-inflammatory drug rofecoxib incorporates a butenolide moiety as a key pharmacophore. In addition, numerous other natural products and synthetic analogues bearing the butenolide core have shown cytotoxic, immunosuppressive, or enzyme-inhibitory activities (Scheme 1A).^2^ These examples collectively underscore the pharmacological importance of this scaffold and have inspired intense interest in the development of efficient synthetic approaches to its construction.

**Scheme 1:**
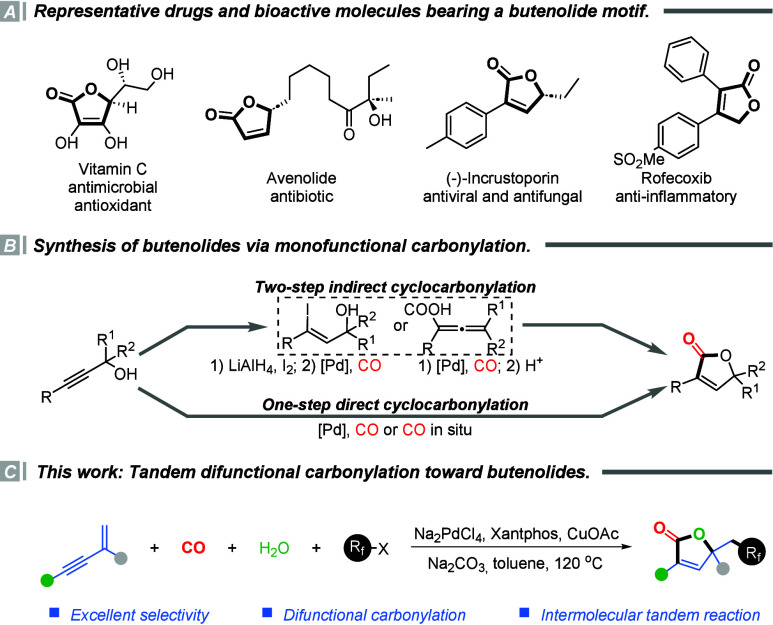
Catalytic Carbonylation for the Synthesis of Butenolides

Over the past several decades, a multitude of synthetic strategies have been developed for assembling butenolide frameworks.^3^ Among them, transition-metal-catalyzed carbonylation reactions have emerged as particularly powerful tools, offering one of the most straightforward and efficient approaches to constructing butenolide skeletons. To date, the synthesis of butenolides via carbonylation has primarily relied on the intramolecular cyclocarbonylation of propargyl alcohols, typically employing one of two general pathways (Scheme 1B). The first is a two-step indirect pathway, wherein propargyl alcohols are initially transformed into iodoallyl alcohols or allenyl carboxylic acid intermediates, which subsequently undergo cyclization to furnish the corresponding butenolide products.^4^ The second is a one-step direct cyclocarbonylation, offering a more atom- and step-economical route that enables efficient access to the butenolide core.^5^ However, both strategies predominantly afford monofunctionalized products, thereby limiting the structural diversity and downstream synthetic utility.

Given these limitations, developing difunctional carbonylation reactions capable of simultaneously constructing the butenolide framework while introducing additional functional elements represents a highly desirable yet underexplored direction. Meanwhile, the incorporation of fluoroalkyl groups has emerged as a valuable strategy in drug design and materials science, owing to their ability to modulate electronic properties, metabolic stability, and lipophilicity.^6^

We envision that a tandem bifunctional carbonylation strategy, capable of simultaneously constructing the butenolide scaffold and introducing fluoroalkyl substituents in a single step, will provide an efficient and modular synthetic route to structurally and functionally diverse butenolide derivatives.^7^ Building on our continued interest in carbonylation chemistry^8^ and the broad synthetic utility of 1,3-enynes as versatile building blocks,^9^ we are committed to developing a difunctionalization strategy using 1,3-enynes as substrates to achieve this efficient construction. Herein, we disclose a palladium-catalyzed intermolecular tandem difunctional carbonylation of 1,3-enynes, which enables the efficient one-step synthesis of fluoroalkyl-substituted butenolides from readily available starting materials (Scheme 1C). This transformation not only expands the synthetic utility of carbonylation chemistry but also provides access to novel fluorinated scaffolds with potential applications in pharmaceutical and materials science.

To explore the feasibility of this intermolecular tandem difunctional carbonylation for the construction of fluoroalkyl-substituted butenolides, we initiated our investigation using 1,3-enyne (**1a**), H_2_O (**2a**), and iodotrifluoromethane (**3a**) as model substrates. The reaction was conducted with Pd­(cod)­Cl_2_ as the catalyst precursor, CuOAc as an additive, and Na_2_CO_3_ as a base, using toluene as the solvent under 10 bar of CO at 100 °C. We first evaluated the effect of ligands on the reaction outcome (Table 1, entries 1–7). Under monodentate phosphine conditions, electron-rich BuPAd_2_ failed to promote the reaction. Interestingly, the use of triphenylphosphine (Table 1, entry 2) afforded the desired product **4a** in 2% yield, thereby providing preliminary validation of our reaction design despite its low efficiency. Encouraged by this result, we next examined a series of bidentate phosphine ligands. Notably, tuning the bite angle had a pronounced impact on reactivity. While dppe and dppp, featuring relatively small bite angles, were ineffective, the use of Xantphos significantly improved the reaction efficiency, affording the target product in 39% yield (Table 1, entries 3–7). This result highlights the importance of the ligand geometry in facilitating the tandem difunctional carbonylation process. We next investigated the effect of Lewis acid additives on the reaction outcome. A variety of mono- and divalent metal salts, including Cu­(I), Cu­(II), Fe­(II), Co­(II), and Zn­(II) species, were examined (Table 1, entries 8–14). However, none outperformed CuOAc, which proved to be the most effective additive under the standard conditions. We then evaluated the influence of the CuOAc loading on the reaction efficiency. Amounts both smaller and larger than the standard 10 mol % led to diminished yields of the desired product (Table 1, entry 15). Notably, no product formation was observed in the absence of CuOAc, indicating that this additive plays a crucial role in enabling the transformation (Table 1, entry 16). These results suggest that CuOAc is essential for the success of the tandem difunctional carbonylation, likely serving as a key Lewis acid cocatalyst involved in the activation of one or more components during the catalytic cycle. To further improve the catalytic efficiency and overall yield, we screened a variety of palladium precatalysts. However, no significant enhancement was observed with alternative Pd sources under standard conditions (Table 1, entries 17–19). Interestingly, during the evaluation of the CO pressure, we found that reducing the pressure to 1 atm led to a notable increase in product yield to 55% (Table 1, entry 20). Building on this result, we tested Na_2_PdCl_4_, a more soluble palladium source, which further improved the yield to 63% (Table 1, entry 21). At the end of each reaction, we consistently observed the formation of palladium Pd black, suggesting catalyst decomposition during the reaction, which could account for the moderate yields obtained in earlier experiments. Based on this hypothesis, we increased the catalyst loading to 15 mol %, which led to a further improvement in efficiency, affording a GC yield of 75% and an isolated yield of 70% for the desired product (Table 1, entry 22).

**Table 1:**
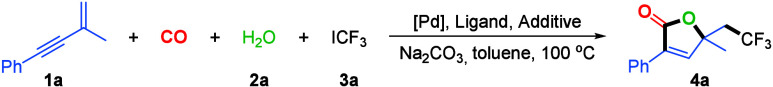
Optimization of the Reaction Conditions^a^,^b^

entry	catalyst	ligand	additive	yield of **4a** (%)^c^
1	Pd(cod)Cl_2_	BuPAd_2_	CuOAc	NR
2	Pd(cod)Cl_2_	PPh_3_	CuOAc	2
3	Pd(cod)Cl_2_	DPPE	CuOAc	NR
4	Pd(cod)Cl_2_	DPPP	CuOAc	NR
5	Pd(cod)Cl_2_	DPPF	CuOAc	5
6	Pd(cod)Cl_2_	Dpephos	CuOAc	19
7	Pd(cod)Cl_2_	Xantphos	CuOAc	39
8	Pd(cod)Cl_2_	Xantphos	CuCl	4
9	Pd(cod)Cl_2_	Xantphos	CuBr	NR
10	Pd(cod)Cl_2_	Xantphos	CuTC	NR
11	Pd(cod)Cl_2_	Xantphos	Cu(OAc)_2_	15
12	Pd(cod)Cl_2_	Xantphos	Co(OAc)_2_	5
13	Pd(cod)Cl_2_	Xantphos	FeCl_3_	3
14	Pd(cod)Cl_2_	Xantphos	ZnCl_2_	3
15	Pd(cod)Cl_2_	Xantphos	CuOAc	9–27^c^,^d^
16	Pd(cod)Cl_2_	Xantphos	–	NR
17	Pd(OAc)_2_	Xantphos	CuOAc	4
18	PdCl_2_	Xantphos	CuOAc	16
19	Pd(PPh_3_)_4_	Xantphos	CuOAc	4
20	Pd(cod)Cl_2_	Xantphos	CuOAc	55^c^
21	Na_2_PdCl_4_	Xantphos	CuOAc	63^c^
22	Na_2_PdCl_4_	Xantphos	CuOAc	75 (70)^e^

a Reaction conditions: **1a** (0.1 mmol), **2a** (10 μL), **3a** (0.3 mmol), [Pd] (10 mol %), ligand (for mono-P, 20 mol %; for bis-P, 10 mol %), Na_2_CO_3_ (0.3 mmol), additive (10 mol %), CO (10 bar), toluene (0.5 mL), 100 °C, 36 h.

b The yield was determined by GC using *^n^* hexadecane as the internal standard.

c Under 1 bar of CO and 5 bar of N_2_.

d CuOAc (2–5 or 15–20 mol %).

e Na_2_PdCl_4_ (15 mol %), Xantphos (15 mol %), CuOAc (15 mol %), 120 °C, 18 h.

With the optimized catalytic system in hand, we next explored the substrate scope of this tandem difunctional carbonylation for the synthesis of fluoroalkyl-substituted butenolides. We first investigated the reactivity of various 1,3-enynes (Scheme 2, top). A range of *para*-substituted aryl 1,3-enynes bearing electron-donating groups (e.g., Me, OMe, and OCF_3_) or halogens (F and Cl) reacted smoothly to afford the corresponding butenolide products in moderate to good yields (**4a**–**4d**, **4f**, and **4g**). Similarly, electron-withdrawing substituents such as CF_3_ and ester groups were also well tolerated (**4h** and **4i**, respectively). Notably, bromo-substituted aryl 1,3-enyne failed to deliver the desired product, possibly due to catalyst deactivation or competitive oxidative addition (**4e**). For the *ortho*-substituted aryl enynes, including those with sterically hindered groups such as *o*-Me or even *o*-F, they were unreactive under the standard conditions (**4j** or **4k**, respectively). This suggests that steric hindrance at the *ortho* position may impede the cyclization step of the tandem process. Aryl 1,3-enynes bearing *meta* substituents, such as F and NO_2_, exhibited good reactivity, affording the desired products in 52% (**4l**) and 72% (**4m**) yields, respectively. The methodology was further extended to other aryl-substituted 1,3-enynes, including those derived from naphthyl and thiophenyl moieties, which also proved to be compatible with the reaction conditions (**4n** and **4o**, respectively). Encouraged by these findings, we further evaluated alkyl-substituted 1,3-enynes, including those bearing linear (phenylpropyl and octyl) and heteroatom-containing substituents such as OTBS. Gratifyingly, all of these substrates were well tolerated, delivering the corresponding fluoroalkylated butenolides in synthetically useful yields (**4p**–**4r**). Finally, we examined the effect of alkene substitution on the reaction efficiency by varying the R^2^ and R^3^ groups in the 1,3-enyne substrates. When R^2^ was changed to an ethyl group, product **4s** was obtained in a moderate yield. In contrast, when R^3^ was substituted with a phenyl group, the reaction was completely suppressed, likely due to electronic or steric interference that hindered the tandem cyclization process. This phenomenon was further improved by the failure with but-3-en-1-yn-1-ylbenzene, (*E*)-pent-3-en-1-yn-1-ylbenzene, but-3-en-1-yne-1,3-diyldibenzene, and (3-(trifluoromethyl)­but-3-en-1-yn-1-yl)­benzene. Only a trace amount of the desired product was detected, while the substrates were transformed with a messy reaction mixture.

**Scheme 2:**
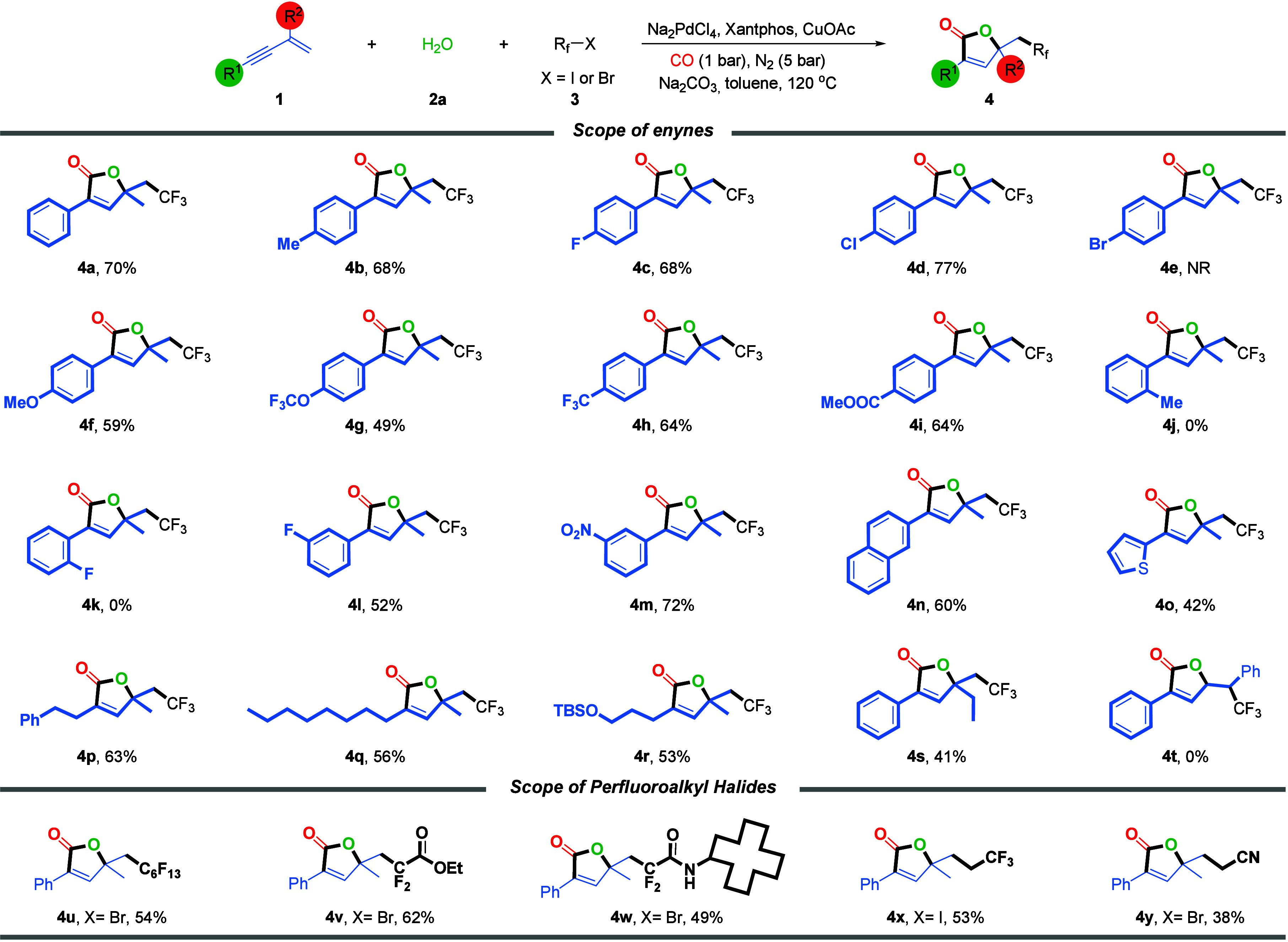
Scope of Enynes and Fluoroalkyl Radical Precursors^a^,^b^

a Reaction conditions: **1a** (0.1 mmol), **2a** (10 μL), **3a** (0.3 mmol), Na_2_PdCl_4_ (15 mol %), Xantphos (15 mol %), Na_2_CO_3_ (0.3 mmol), CuOAc (15 mol %), CO (1 bar), toluene (0.5 mL), 120 °C, 18 h.
b Isolated yields.

Subsequently, we turned our attention to the scope of fluoroalkyl halides (Scheme 2, bottom). A range of electrophilic fluoroalkylating agents—including perfluorohexyl iodide, bromodifluoroacetate, and bromodifluoroacetamide—underwent the transformation smoothly, delivering butenolide products **4u**–**4w**, respectively, in moderate yields. Notably, 2-iodo-1,1,1-trifluoroethane and bromoacetonitrile were also found to be compatible under the standard conditions, affording the desired products in 53% (**4x**) and 38% (**4y**) yields, respectively. However, a low yield of the desired product was obtained when MeI was tested as the substrate under our standard conditions.

To gain insight into the mechanism of this tandem difunctional fluoroalkylative carbonylation, a series of control and mechanistic experiments were carried out (Scheme 3). We first performed radical inhibition experiments. The addition of radical scavengers such as TEMPO or 1,1-diphenylethylene completely suppressed the formation of product **4a**, indicating that the reaction likely proceeds via a radical-mediated pathway. Next, we conducted a deuterium labeling experiment using D_2_O instead of H_2_O under the standard conditions. The reaction afforded deuterated product **4a-D** in 61% yield, with 47% deuterium incorporation observed at the olefinic proton based on ^1^H NMR analysis. No significant H/D scrambling was detected, suggesting that the olefinic hydrogen originates directly from water (D_2_O). The moderate incorporation of deuterium could be attributed to residual H_2_O in the reaction medium. These observations led us to hypothesize the involvement of a fluoroalkylated allenyl carboxylic acid intermediate (**5a**) in the reaction pathway. To test this, independently synthesized **5a** was subjected to the standard reaction conditions, resulting in a 62% yield of butenolide **4a**. This supports the role of **5a** as a plausible intermediate. Interestingly, in the absence of the palladium catalyst, the cyclization of **5a** still proceeded, affording the product in 68% yield, suggesting that Pd is not essential for the cyclization step. Further investigation revealed that the addition of CuOAc alone to the reaction mixture led to an even higher yield (83%), highlighting the crucial role of CuOAc in promoting the cyclization process. To further probe the role of CuOAc in the catalytic cycle, we conducted a sequential one-pot, two-step experiment. The reaction was first carried out in the absence of CuOAc, and after completion of the initial stage, CuOAc was added to the reaction mixture, followed by continued stirring under the standard conditions. Interestingly, no formation of the desired butenolide product **4a** was observed in this stepwise protocol. These findings suggest that CuOAc is involved in multiple stages of the reaction pathway and functions synergistically with palladium to facilitate both intermediate formation and final product assembly.

**Scheme 3:**
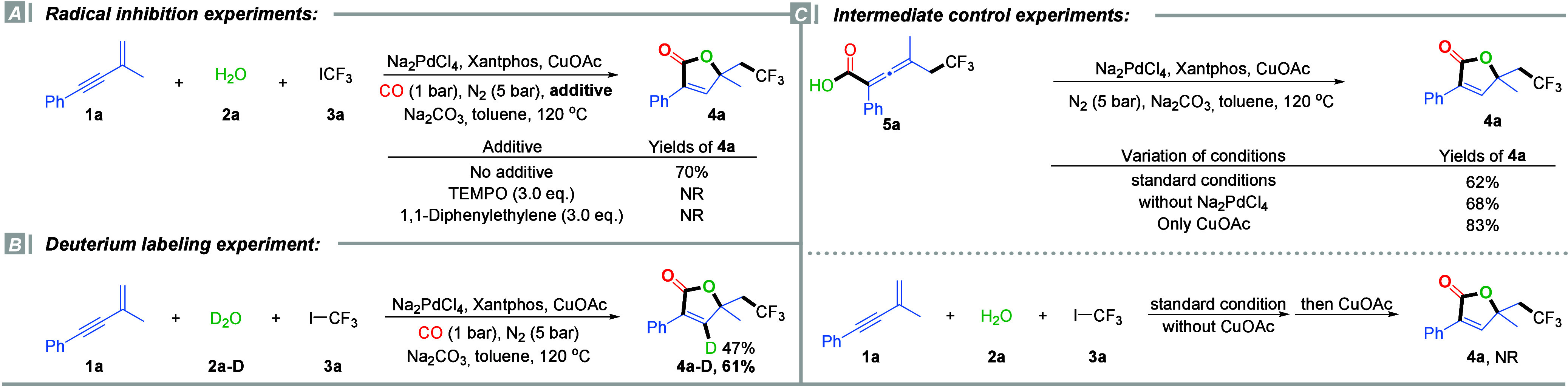
Mechanistic Studies

Based on the mechanistic studies and related literature precedents,^10^ a plausible catalytic cycle is proposed in Scheme 4. The cycle begins with the in situ generation of catalytically active Pd^0^Ln species **A** from the Pd­(II) precatalyst under ligand-assisted reduction. Resulting Pd^0^ complex **A** then engages in a single-electron transfer (SET) with trifluoroiodomethane (CF_3_-I), producing trifluoromethyl radical **B** and Pd­(I) species **E** (Pd^I^LnX). CF_3_ radical **B** could be stabilized by a copper catalyst and readily adds to the 1,3-enyne substrate, forming tertiary propargyl radical intermediate **C**, which undergoes a rapid radical isomerization to generate more stable allenyl radical **D**. Pd­(I) species **E** then reincorporated allenyl radical **D** to form Pd­(II)–allenyl complex **F**, which undergoes CO insertion to generate acyl–Pd­(II) intermediate **G**. In the presence of CuOAc as a cocatalyst and H_2_O as the nucleophile, intermediate **G** undergoes a nucleophilic attack to form carboxylic acid intermediate **5a**. Concurrently, reductive elimination, facilitated by the base, regenerates the Pd^0^Ln species, thereby completing the catalytic cycle. Finally, compound **5a** undergoes CuOAc-promoted intramolecular cyclization through a copper­(I) carboxylate intermediate and then lactonization to furnish the desired fluoroalkylated butenolide product.

**Scheme 4:**
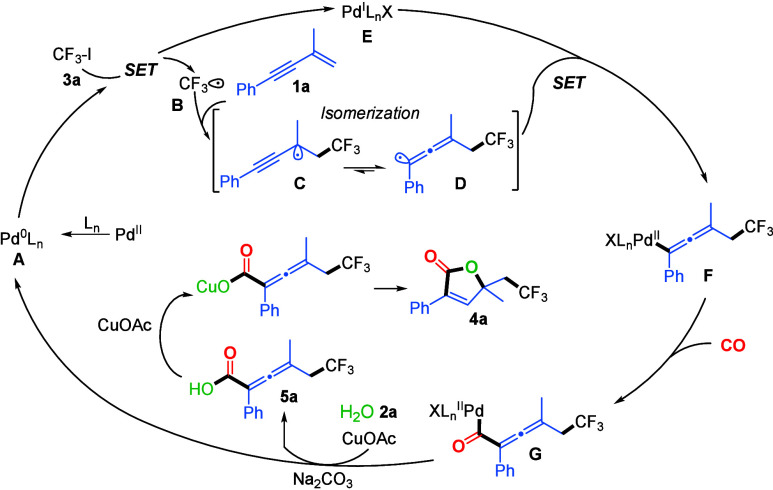
Proposed Mechanism

In summary, we have developed a palladium-catalyzed intermolecular tandem difunctional carbonylation of 1,3-enynes, enabling the efficient and modular one-step synthesis of fluoroalkyl-substituted butenolides from readily available starting materials. This transformation features a broad substrate scope, good functional group compatibility, and high chemo- and regioselectivity. Mechanistic studies suggest the involvement of fluoroalkyl radicals and a Pd/Cu-cooperative catalysis cycle, providing insight into the reaction pathway. Overall, this strategy not only broadens the synthetic utility of carbonylation chemistry but also offers streamlined access to structurally diverse fluorinated butenolide scaffolds with promising applications in drug discovery and development of functional materials.

## Supplementary Material



## Data Availability

The data underlying this study are available in the published article and its Supporting Information.

## Notes

Experimental details, characterization data, and NMR spectra (PDF)
