# Supplementary material for: Palladium-Catalyzed Intermolecular Tandem Difunctional Carbonylation of 1,3-Enynes: Synthesis of Fluoroalkylated Butenolides
Source: Org Lett. 2025 Jul 25;27(31):8811–6. doi: 10.1021/acs.orglett.5c03004 (PMC12340975; doi:10.1021/acs.orglett.5c03004)

Supporting Information

Palladium-Catalyzed Intermolecular Tandem Difunctional Carbonylation of 1,3-Enynes: Synthesis Fluoroalkylated Butenolides

Chang-Sheng Kuai[a], [b], Ru-Han A[a,c], Zhi-Peng Bao[a,c], and Xiao-Feng Wu[a], [b], [c]*

[a] Dalian National Laboratory for Clean Energy, Dalian Institute of Chemical Physics, Chinese Academy of Sciences, Dalian 116023 China, E-mail: xwu2020@dicp.ac.cn

[b] University of Chinese Academy of Sciences, Beijing 100049, China

[c] Leibniz-Institut für Katalyse e. V., Albert-Einstein-Straβe 29a, 18059 Rostock, Germany

Table of Contents

1. General experimental details **S2**

2. Typical procedure for Pd-catalyzed tandem difunctional carbonylation of 1,3-enynes. **S3**

3. Radical inhibition experiment. **S4**

4. Deuterium labeling experiment. **S5**

5. Intermediate control experiments **S6**

6. Spectroscopic Data of Products. **S7**

7. NMR Spectra of the Products. **S14**

1. General experimental details

Unless otherwise noted, all reactions were carried out under a carbon monoxide or nitrogen atmosphere. The amines and reagents were ordered from Adamas-beta®, Energy Chemical Sigma-Aldrich, Bidepharm and used without purification. CF_3I (CAS: 2314-97-8; 25 wt.% solution in THF) was ordered from Energy Chemical and used as received. 1,3-Enynes (**1**) were prepare according literature.[1] All solvents were dried by standard techniques and distilled prior to use. Column chromatography was performed on silica gel (200-300 meshes). All NMR spectra were recorded at ambient temperature using Bruker Avance III 400 MHz NMR (^{1}H, 400 MHz; ^{13}C {^{1}H}, 101 MHz, ^{19}F 376 MHz), Bruker AVANCE III HD 700MHz NMR spectrometers (^{1}H, 700 MHz; ^{13}C{^{1}H}, 100 MHz). ^{1}H NMR chemical shifts are reported relative to TMS and were referenced via residual proton resonances of the corresponding deuterated solvent ($CDCl_3$: 7.26 ppm) whereas ^{13}C{^{1}H} NMR spectra are reported relative to TMS via the carbon signals of the deuterated solvent ($CDCl_3$: 77.0 ppm). Data for ^{1}H are reported as follows: chemical shift (δ ppm), multiplicity (s = singlet, d = doublet, t = triplet, q = quartet, quint = quintet, m = multiplet, br = broad), coupling constant (Hz), and integration. All ^{13}C NMR spectra were broad-band ^{1}H decoupled. All reactions were monitored by GC-FID or NMR analysis. HRMS data was obtained with Micromass HPLC-Q-TOF mass spectrometer (ESI) or Agilent 6540 Accurate-MS spectrometer (Q-TOF).

Because of the high toxicity of carbon monoxide, all the reactions should be performed in an autoclave. The laboratory should be well-equipped with a CO detector and alarm system.

1. Zhang, K.-F.; Bian, K.-J.; Li, C.; Sheng, J.; Li, Y.; Wang, X.-S. Nickel-Catalyzed Carbofluoroalkylation of 1,3-Enynes to Access Structurally Diverse Fluoroalkylated Allenes. *Angew. Chem., Int. Ed.* **2019**, *58*, 5069-5074.

2. Typical procedure for Pd-catalyzed tandem difunctional carbonylation of 1,3-enynes.

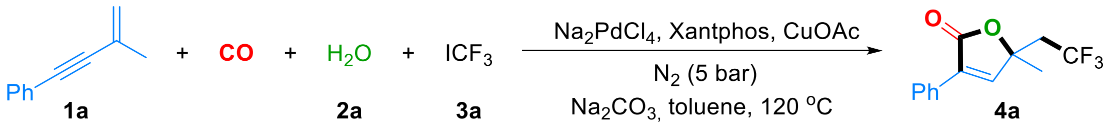

A 4 mL screw-cap vial was charged with Na_2PdCl_4 (15 mol%; 4.41 mg), Xantphos (15 mol%; 8.67 mg), CuOAc (15 mol%; 1.83 mg), Na_2CO_3 (0.3 mmol; 31.8 mg; 3 equiv.), and an oven-dried stir bar. The vial was closed with a Teflon septum and cap and connected to the atmosphere via a needle. After toluene (0.5 mL), 1,3-enyne (**1a**, 0.10 mmol; 14.2 mg; 1 equiv.), H_2O (**2a**, 10 μL) and iodotrifluoromethane (**3a**, 0.30 mmol; 3 equiv.; 225 μL), were added with a syringe under argon atmosphere, the vial was moved to an alloy plate and put into a Parr 4560 series autoclave (300 mL) under an argon atmosphere. At room temperature, the autoclave was flushed with CO three times and charged with 1 bar of CO and 5 bar of N_2. The autoclave was placed on a heating plate equipped with a magnetic stirrer and an aluminum block. The reaction mixture was heated to 120 °C for 18h. After the reaction was complete, the autoclave was cooled down with ice water to room temperature and the pressure was released carefully. The reaction mixture concentration under reduced pressure, the crude product was purified by column chromatography on silica gel to afford the corresponding product **4a**.

1 mmol scale: A 12 mL screw-cap vial was charged with Na_2PdCl_4 (15 mol%), Xantphos (15 mol%), CuOAc (15 mol%), Na_2CO_3 (3 mmol), and an oven-dried stir bar. The vial was closed with a Teflon septum and cap and connected to the atmosphere via a needle. After toluene (5 mL), 1,3-enyne (**1a**, 1.0 mmol; 1 equiv.), H_2O (**2a**, 100 μL) and iodotrifluoromethane (**3a**, 3 mmol; 1 equiv.), were added with a syringe under argon atmosphere, the vial was moved to an alloy plate and put into a Parr 4560 series autoclave (300 mL) under an argon atmosphere. At room temperature, the autoclave was flushed with CO three times and charged with 1 bar of CO and 5 bar of N_2. The autoclave was placed on a heating plate equipped with a magnetic stirrer and an aluminum block. The reaction mixture was heated to 120 °C for 18h. After the reaction was complete, the autoclave was cooled down with ice water to room temperature and the pressure was released carefully. The reaction mixture concentration under reduced pressure, the crude product was purified by column chromatography on silica gel to afford the corresponding product **4a** in 60% yield (153.6 mg).

3. Radical inhibition experiment.

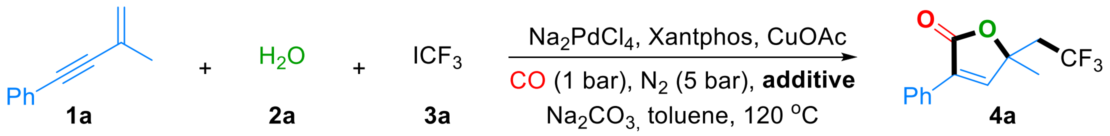

Additive	Yields of **4a**
No additive	70%
TEMPO (3.0 eq.)	NR
1,1-Diphenylethylene (3.0 eq.)	NR

A 4 mL screw-cap vial was charged with Na_2PdCl_4 (15 mol%), Xantphos (15 mol%), CuOAc (15 mol%), Na_2CO_3 (0.3 mmol), radical scavenger TEMPO or 1,1-Diphenylethylene (0.3 mmol) and an oven-dried stir bar. The vial was closed with a Teflon septum and cap and connected to the atmosphere via a needle. After toluene (0.5 mL), 1,3-enyne (**1a**, 0.10 mmol), H_2O (**2a**, 10uL) and iodotrifluoromethane (**3a**, 0.30 mmol), were added with a syringe under argon atmosphere, the vial was moved to an alloy plate and put into a Parr 4560 series autoclave (300 mL) under an argon atmosphere. At room temperature, the autoclave was flushed with CO three times and charged with 1 bar of CO and 5 bar of N_2. The autoclave was placed on a heating plate equipped with a magnetic stirrer and an aluminum block. The reaction mixture was heated to 120 °C for 18 hours. Upon completion, the autoclave was cooled to room temperature using an ice-water bath, and the internal pressure was carefully released. However, no desired product **4a** was detected.

4. Deuterium labeling experiment.

A 4 mL screw-cap vial was charged with Na_2PdCl_4 (15 mol%), Xantphos (15 mol%), CuOAc (15 mol%), Na_2CO_3 (0.3 mmol), and an oven-dried stir bar. The vial was closed with a Teflon septum and cap and connected to the atmosphere via a needle. After toluene (0.5 mL), 1,3-enyne (**1a**, 0.10 mmol), D_2O (**2a-D**, 10uL) and iodotrifluoromethane (**3a**, 0.30 mmol), were added with a syringe under argon atmosphere, the vial was moved to an alloy plate and put into a Parr 4560 series autoclave (300 mL) under an argon atmosphere. At room temperature, the autoclave was flushed with CO three times and charged with 1 bar of CO and 5 bar of N_2. The autoclave was placed on a heating plate equipped with a magnetic stirrer and an aluminum block. The reaction mixture was heated to 120 °C for 18h. After the reaction was complete, the autoclave was cooled down with ice water to room temperature and the pressure was released carefully. The reaction mixture concentration under reduced pressure, the crude product was purified by column chromatography on silica gel to afford the corresponding product **4a-D** in 61% yield.

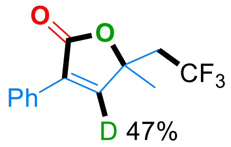

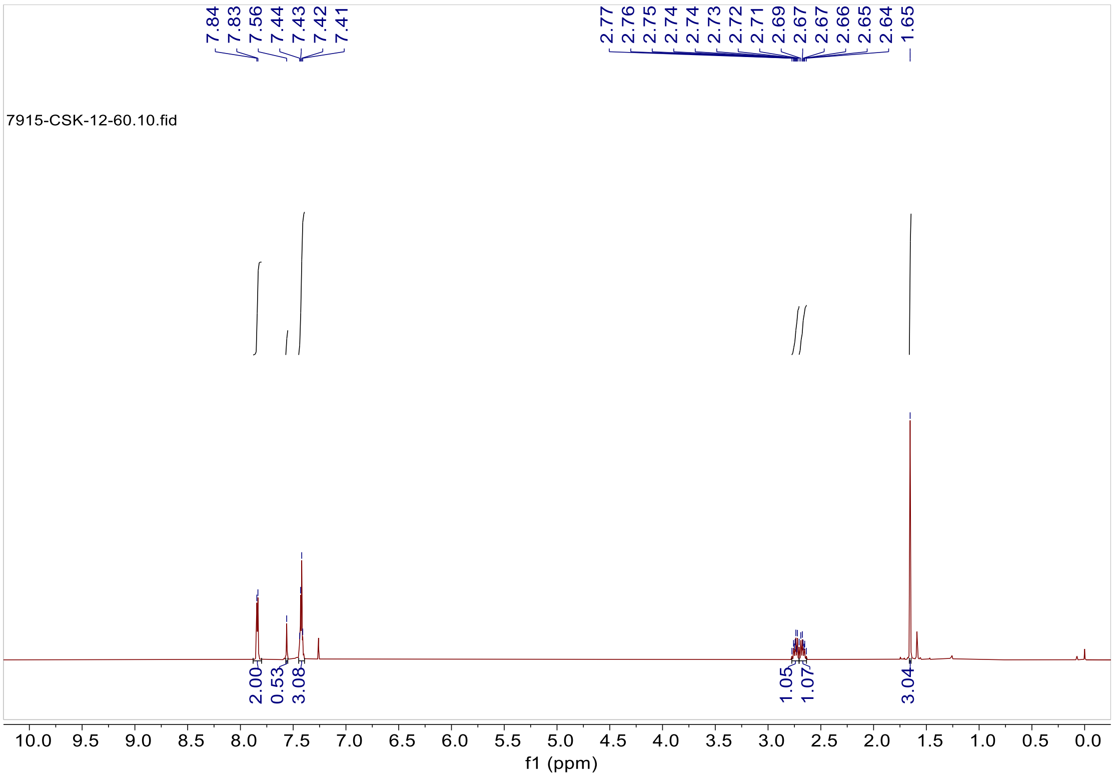

5. Intermediate control experiments.

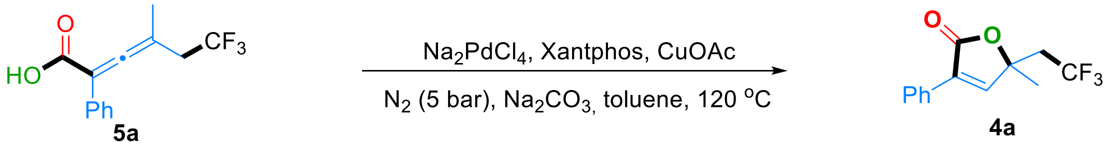

Variation of conditions	Yields of **4a**
standard conditions	62%
without Na_2PdCl_4	68%
Only CuOAc	83%

A 4 mL screw-cap vial was charged with Na_2PdCl_4 (15 mol%), Xantphos (15 mol%), CuOAc (15 mol%), Na_2CO_3 (0.3 mmol), **5a** (0.1 mmol)and an oven-dried stir bar (**variations in other reaction conditions are listed in the table**) .The vial was closed with a Teflon septum and cap and connected to the atmosphere via a needle. After toluene (0.5 mL) were added with a syringe under argon atmosphere, the vial was moved to an alloy plate and put into a Parr 4560 series autoclave (300 mL) under an argon atmosphere. At room temperature, the autoclave was flushed with N_2 three times and charged with 5 bar of N_2. The autoclave was placed on a heating plate equipped with a magnetic stirrer and an aluminum block. The reaction mixture was heated to 120 °C for 18h. After the reaction was complete, the autoclave was cooled down with ice water to room temperature and the pressure was released carefully. The reaction mixture concentration under reduced pressure, the crude product was purified by column chromatography on silica gel to afford the corresponding product **4a**.

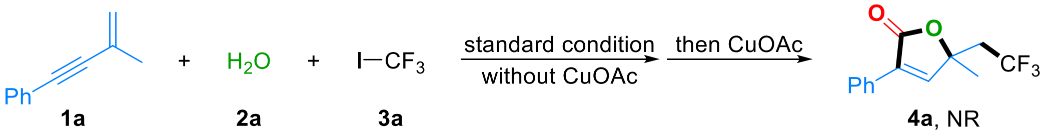

A 4 mL screw-cap vial was charged with Na_2PdCl_4 (15 mol%), Xantphos (15 mol%), Na_2CO_3 (0.3 mmol), and an oven-dried stir bar. The vial was closed with a Teflon septum and cap and connected to the atmosphere via a needle. After toluene (0.5 mL), 1,3-enyne (**1a**, 0.10 mmol), H_2O (**2a**, 10uL) and iodotrifluoromethane (**3a**, 0.30 mmol), were added with a syringe under argon atmosphere, the vial was moved to an alloy plate and put into a Parr 4560 series autoclave (300 mL) under an argon atmosphere. At room temperature, the autoclave was flushed with CO three times and charged with 1 bar of CO and 5 bar of N_2. The autoclave was placed on a heating plate equipped with a magnetic stirrer and an aluminum block. The reaction mixture was heated to 120 °C for 18h.

After completion of the reaction, the autoclave was cooled to room temperature using an ice-water bath, and the internal pressure was carefully released. Subsequently, CuOAc (15 mol%) was added to the reaction mixture, which was then transferred to a 300 mL Parr 4560 series autoclave under an argon atmosphere. The autoclave was flushed with CO three times at room temperature, followed by charging with 1 bar of CO and 5 bar of N_2. It was then placed on a heating plate equipped with a magnetic stirrer and an aluminum block, and the mixture was heated at 120 °C for 18 hours. After the reaction, the system was cooled to room temperature using an ice-water bath, and the pressure was carefully released. However, no desired product **4a** was detected.

6. Spectroscopic Data of Products.

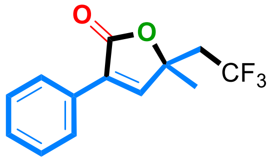

5-Methyl-3-phenyl-5-(2,2,2-trifluoroethyl)furan-2(5H)-one (4a):
Yellow solid, 18.1mg, 70% yield, R_f=0.2 (PE/EtOAc 15/1).
^{1}H NMR (700 MHz, $CDCl_3$) δ 7.88 – 7.80 (m, 2H), 7.56 (s, 1H), 7.44 – 7.40 (m, 3H), 2.78 – 2.64 (m, 2H), 1.66 (s, 3H).
^{13}C NMR (176 MHz, $CDCl_3$) δ169.9, 149.3, 131.5, 129.8, 128.9, 128.8, 127.2, 124.7 (q, J = 277.5 Hz), 80.9 (q, J = 2.2 Hz), 42.6 (q, J = 28.2 Hz), 25.0.
^{19}F NMR (376 MHz, $CDCl_3$) δ -61.65.
HRMS (ESI-TOF) m/z: $[M + H]^+$ calculated for $C_{13}H_{12}F_3O_2$ 257.0784; Found 257.0786.

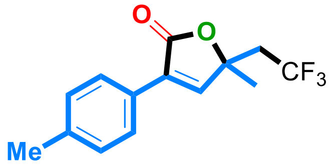

5-Methyl-3-(p-tolyl)-5-(2,2,2-trifluoroethyl)furan-2(5H)-one (4b):
Light brown solid, 18.4mg, 68% yield, R_f=0.2 (PE/EtOAc 15/1).
^{1}H NMR (700 MHz, $CDCl_3$) δ 7.74 (d, J = 8.1 Hz, 2H), 7.50 (s, 1H), 7.23 (d, J = 7.9 Hz, 2H), 2.76 – 2.62 (m, 2H), 2.38 (s, 3H), 1.64 (s, 3H).
^{13}C NMR (100 MHz, $CDCl_3$) δ 170.1, 148.3, 140.0, 131.4, 129.4, 127.1, 126.1, 124.7 (q, J = 277.6 Hz), 80.9 (q, J = 2.2 Hz), 42.6 (q, J = 28.1 Hz), 25.0, 21.4.
^{19}F NMR (376 MHz, $CDCl_3$) δ -61.65.
HRMS (ESI-TOF) m/z: $[M + H]^+$ calculated for $C_{14}H_{14}F_3O_2$ 271.0940; Found 271.0949.

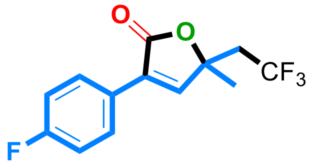

3-(4-Fluorophenyl)-5-methyl-5-(2,2,2-trifluoroethyl)furan-2(5H)-one (4c):
White solid, 18.5mg, 68% yield, R_f=0.2 (PE/EtOAc 15/1).
^{1}H NMR (700 MHz, $CDCl_3$) δ 7.85 (dd, J = 8.7, 5.4 Hz, 2H), 7.52 (s, 1H), 7.12 (t, J = 8.6 Hz, 2H), 2.78 – 2.64 (m, 2H), 1.66 (s, 3H).
^{13}C NMR (176 MHz, $CDCl_3$) δ 169.8, 163.6 (d, *J* = 250.5 Hz), 148.8, 130.5, 129.3 (d, *J* = 8.4 Hz), 125.1 (d, *J* = 3.5 Hz), 124.7 (q, *J* = 277.6 Hz), 115.9 (d, *J* = 21.7 Hz), 81.0 (q, J = 2.0 Hz), 42.6 (q, *J* = 28.2 Hz), 25.0.
^{19}F NMR (376 MHz, $CDCl_3$) δ -61.66, -110.43.
HRMS (ESI-TOF) m/z: $[M + H]^+$ calculated for $C_{13}H_{11}F_4O_2$ 275.0690; Found 275.0688.

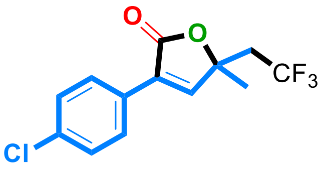

3-(4-Chlorophenyl)-5-methyl-5-(2,2,2-trifluoroethyl)furan-2(5H)-one (4d):

Brown solid, 22.4mg, 77% yield, R_f=0.2 (PE/EtOAc 15/1).

^{1}H NMR (700 MHz, $CDCl_3$) δ 7.80 (d, J = 8.6 Hz, 2H), 7.57 (s, 1H), 7.40 (d, J = 8.6 Hz, 2H), 2.78 – 2.64 (m, 2H), 1.65 (s, 3H).

^{13}C NMR (176 MHz, $CDCl_3$) δ 169.6, 149.5, 135.9, 130.5, 129.0, 128.6, 127.3, 124.6 (q, J = 277.6 Hz), 81.1 (q, J = 2.2 Hz), 42.5 (q, J = 28.3 Hz), 25.0.

^{19}F NMR (376 MHz, $CDCl_3$) δ -61.65.

HRMS (ESI-TOF) m/z: $[M + H]^+$ calculated for $C_{13}H_{11}ClF_3O_2$ 291.0394; Found 291.0395.

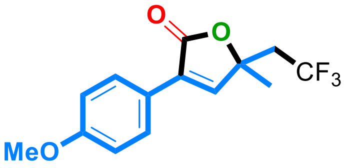

3-(4-Methoxyphenyl)-5-methyl-5-(2,2,2-trifluoroethyl)furan-2(5H)-one (4f):

Brown solid, 16.9mg, 59% yield, R_f=0.2 (PE/EtOAc 10/1).

^{1}H NMR (700 MHz, $CDCl_3$) δ 7.82 (d, J = 8.8 Hz, 2H), 7.44 (s, 1H), 6.94 (d, J = 8.8 Hz, 2H), 3.84 (s, 3H), 2.75 – 2.62 (m, 2H), 1.64 (s, 3H).

^{13}C NMR (176 MHz, $CDCl_3$) δ 170.2, 160.8, 147.0, 130.8, 128.7, 124.7 (q, J = 277.6 Hz), 121.5, 114.1, 80.9 (q, J = 2.2 Hz), 55.4, 42.7 (q, J = 28.2 Hz), 25.0.

^{19}F NMR (376 MHz, $CDCl_3$) δ -61.64.

HRMS (ESI-TOF) m/z: $[M + H]^+$ calculated for $C_{14}H_{14}F_3O_3$ 287.0890; Found 287.0894.

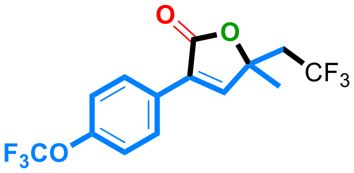

5-Methyl-5-(2,2,2-trifluoroethyl)-3-(4-(trifluoromethoxy)phenyl)furan-2(5H)-one (4g):

Light yellow solid, 16.0mg, 49% yield, R_f=0.2 (PE/EtOAc 10/1).

^{1}H NMR (700 MHz, $CDCl_3$) δ 7.90 (d, J = 8.8 Hz, 2H), 7.58 (s, 1H), 7.28 (d, J = 8.3 Hz, 2H), 2.79 – 2.66 (m, 2H), 1.67 (s, 3H).

^{13}C NMR (176 MHz, $CDCl_3$) δ 169.6, 150.1 (q, J = 1.8 Hz), 149.7, 130.4, 128.9, 127.5, 124.6 (q, J = 277.6 Hz), 120.04 (q, J = 257.8 Hz), 119.7, 81.1 (q, J = 2.4 Hz), 42.5 (q, J = 28.3 Hz), 25.0.

^{19}F NMR (376 MHz, $CDCl_3$) δ -57.77, -61.68.

HRMS (ESI-TOF) m/z: $[M + H]^+$ calculated for $C_{14}H_{11}F_6O_3$ 341.0607; Found 341.0602.

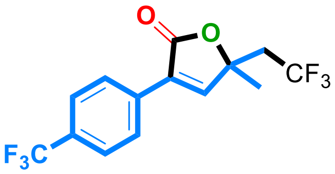

5-Methyl-5-(2,2,2-trifluoroethyl)-3-(4-(trifluoromethyl)phenyl)furan-2(5H)-one (4h):

Light yellow solid, 20.6mg, 64% yield, R_f=0.2 (PE/EtOAc 15/1).

^{1}H NMR (700 MHz, $CDCl_3$) δ 7.97 (d, J = 8.1 Hz, 2H), 7.70 (s, 1H), 7.68 (d, J = 6.0 Hz, 2H), 2.80 – 2.69 (m, 2H), 1.68 (s, 3H).

^{13}C NMR (176 MHz, $CDCl_3$) δ 169.3, 151.1, 132.3, 131.6 (q, J = 32.7 Hz), 130.5, 127.6, 125.7 (q, J = 3.7 Hz), 124.6 (q, J = 277.6 Hz), 123.8 (q, J = 272.3 Hz), 81.2 (q, J = 2.1 Hz), 42.5 (q, J = 28.4 Hz), 24.9.

^{19}F NMR (376 MHz, $CDCl_3$) δ -61.67, -62.92.

HRMS (ESI-TOF) m/z: $[M + H]^+$ calculated for $C_{14}H_{11}F_6O_2$ 325.0658; Found 325.0655.

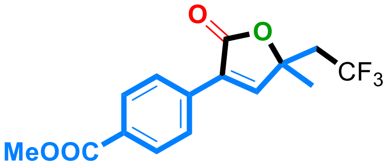

Methyl 4-(5-methyl-2-oxo-5-(2,2,2-trifluoroethyl)-2,5-dihydrofuran-3-yl)benzoate (4i):

Brown solid, 20.2mg, 64% yield, R_f=0.2 (PE/EtOAc 15/1).

^{1}H NMR (700 MHz, $CDCl_3$) δ 8.09 (d, J = 8.6 Hz, 2H), 7.93 (d, J = 8.6 Hz, 2H), 7.69 – 7.67 (m, 1H), 3.94 (s, 3H), 2.79 – 2.68 (m, 2H), 1.68 (s, 3H).

^{13}C NMR (176 MHz, $CDCl_3$) δ 169.4, 166.5. 151.1, 133.1, 131.1, 130.8, 129.9, 127.2, 124.6 (q, J = 277.6 Hz), 81.2 (q, J = 2.3 Hz), 52.3, 42.5 (q, J = 28.3 Hz), 24.9.

^{19}F NMR (376 MHz, $CDCl_3$) δ -61.64.

HRMS (ESI-TOF) m/z: $[M + H]^+$ calculated for $C_{15}H_{14}F_3O_4$ 315.0839; Found 315.0842.

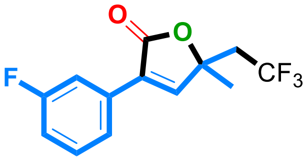

3-(3-Fluorophenyl)-5-methyl-5-(2,2,2-trifluoroethyl)furan-2(5H)-one (4l):

Brown solid, 14.2mg, 52% yield, R_f=0.2 (PE/EtOAc 15/1).

^{1}H NMR (700 MHz, $CDCl_3$) δ 7.65 – 7.56 (m, 3H), 7.44 – 7.36 (m, 1H), 7.15 – 7.09 (m, 1H), 2.79 – 2.72 (m, 1H), 2.72 – 2.65 (m, 1H), 1.66 (s, 3H).

^{13}C NMR (176 MHz, $CDCl_3$) δ 169.4, 162.8 (d, J = 246.6 Hz), 150.2, 130.8 (d, J = 8.4 Hz), 130.5 (d, J = 2.3 Hz), 130.4 (d, J = 8.3 Hz), 124.6 (q, J = 277.6 Hz), 122.9 (d, J = 3.0 Hz), 116.8 (d, J = 21.1 Hz), 114.3 (d, J = 23.3 Hz), 81.0 (q, J = 2.0 Hz), 42.5 (q, J = 28.4 Hz), 24.9.

^{19}F NMR (376 MHz, $CDCl_3$) δ -61.67, -111.96.

HRMS (ESI-TOF) m/z: $[M + H]^+$ calculated for $C_{13}H_{11}F_4O_2$ 257.0690; Found 257.0694.

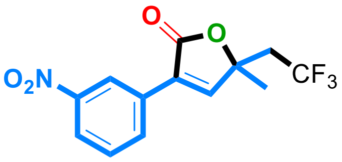

5-Methyl-3-(3-nitrophenyl)-5-(2,2,2-trifluoroethyl)furan-2(5H)-one (4m):

Brown solid, 21.6mg, 72% yield, R_f=0.2 (PE/EtOAc 10/1).

^{1}H NMR (700 MHz, $CDCl_3$) δ 8.66 (t, J = 1.8 Hz, 1H), 8.30 – 8.26 (m, 2H), 7.77 – 7.75 (m, 1H), 7.64 (t, J = 8.0 Hz, 1H), 2.81 – 2.72 (m, 2H), 1.70 (s, 3H).

^{13}C NMR (176 MHz, $CDCl_3$) δ 169.0, 151.5, 148.5, 133.1, 130.5, 130.0, 129.7, 124.5 (q, J = 277.6 Hz), 124.4, 122.2, 81.4 (q, J = 2.3 Hz), 42.4 (q, J = 28.4 Hz), 25.0.

^{19}F NMR (376 MHz, $CDCl_3$) δ -61.64.

HRMS (ESI-TOF) m/z: $[M + H]^+$ calculated for $C_{13}H_{11}F_3NO_4$ 302.0635; Found 302.0636.

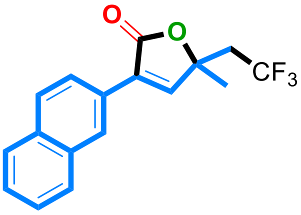

5-Methyl-3-(naphthalen-2-yl)-5-(2,2,2-trifluoroethyl)furan-2(5H)-one (4n):

White solid, 18.3mg, 60% yield, R_f=0.2 (PE/EtOAc 15/1).

^{1}H NMR (700 MHz, $CDCl_3$) δ 8.58 (s, 1H), 7.94 – 7.91 (m, 1H), 7.88 (d, J = 8.5 Hz, 1H), 7.86 – 7.82 (m, 1H), 7.76 (dd, J = 8.5, 1.6 Hz, 1H), 7.67 (s, 1H), 7.53 (p, J = 6.1 Hz, 2H), 2.80 – 2.68 (m, 2H), 1.70 (s, 3H).

^{13}C NMR (176 MHz, $CDCl_3$) δ 167.0, 149.3, 133.7, 133.1, 131.3, 128.9, 128.5, 127.7, 127.3, 127.2, 126.7, 126.1, 124.7 (q, J = 277.5 Hz), 124.1, 81.0 (q, J = 2.5 Hz), 42.7 (q, J = 28.1 Hz), 25.0.

^{19}F NMR (376 MHz, $CDCl_3$) δ -61.64.

HRMS (ESI-TOF) m/z: $[M + H]^+$ calculated for $C_{17}H_{14}F_3O_2$ 307.0940; Found 307.0943.

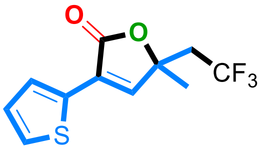

5-Methyl-3-(thiophen-2-yl)-5-(2,2,2-trifluoroethyl)furan-2(5H)-one (4o):

Black solid, 11.1mg, 42% yield, R_f=0.2 (PE/EtOAc 10/1).

^{1}H NMR (700 MHz, $CDCl_3$) δ 7.79 – 7.76 (m, 1H), 7.42 – 7.41 (m, 1H), 7.38 (s, 1H), 7.10 (dd, J = 5.0, 3.7 Hz, 1H), 2.77 – 2.70 (m, 1H), 2.68 – 2.62 (m, 1H), 1.65 (s, 3H).

^{13}C NMR (176 MHz, $CDCl_3$) δ 169.1, 145.3, 130.8, 128.2, 127.9, 127.9, 126.4, 124.6 (q, J = 277.4 Hz), 81.8 (q, J = 2.3 Hz), 42.6 (q, J = 28.3 Hz), 25.0.

^{19}F NMR (376 MHz, $CDCl_3$) δ -61.64.

HRMS (ESI-TOF) m/z: $[M + H]^+$ calculated for $C_{11}H_{10}F_3O_2S$ 263.0348; Found 263.0346.

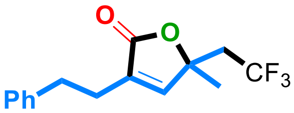

6,6,6-Trifluoro-4-methyl-2-(naphthalen-2-yl)hexa-2,3-dienamide (4p):

Brown oil, 17.4mg, 63% yield, R_f=0.2 (PE/EtOAc 15/1).

^{1}H NMR (700 MHz, $CDCl_3$) δ 7.29 (t, J = 7.5 Hz, 2H), 7.20 (t, J = 7.3 Hz, 1H), 7.16 (d, J = 7.5 Hz, 2H), 6.88 (s, 1H), 2.89 (t, J = 7.6 Hz, 2H), 2.63 (t, J = 7.7 Hz, 2H), 2.60 – 2.54 (m, 1H), 2.50 – 2.43 (m, 1H), 1.47 (s, 3H).
^{13}C NMR (176 MHz, $CDCl_3$) δ 171.8, 151.0, 140.2, 133.3, 128.5, 128.5, 126.4, 124.6 (q, J = 277.6 Hz), 81.7 (q, J = 2.5 Hz), 42.4 (q, J = 28.2 Hz), 33.2, 26.7, 24.7.
^{19}F NMR (376 MHz, $CDCl_3$) δ -61.72.
HRMS (ESI-TOF) m/z: $[M + H]^+$ calculated for $C_{15}H_{16}F_3O_2$ 285.1097; Found 285.1095.

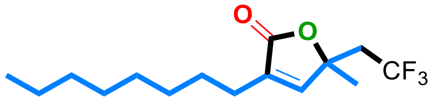

5-Methyl-3-octyl-5-(2,2,2-trifluoroethyl)furan-2(5H)-one (4q):
Yellow oil, 16.3mg, 56% yield, R_f=0.2 (PE/EtOAc 15/1).
^{1}H NMR (700 MHz, $CDCl_3$) δ 7.02 (s, 1H), 2.68 – 2.52 (m, 2H), 2.27 (t, J = 7.7 Hz, 2H), 1.54 (s, 5H), 1.29 (ddt, J = 28.6, 14.5, 9.1 Hz, 10H), 0.88 (t, J = 7.1 Hz, 3H).
^{13}C NMR (176 MHz, $CDCl_3$) δ 172.0, 149.7, 134.7, 124.7 (q, J = 277.6 Hz), 81.6 (q, J = 2.1 Hz), 42.5 (q, J = 28.2 Hz), 31.8, 29.2, 29.2, 29.1, 27.3, 25.1, 25.0, 22.6, 14.1.
^{19}F NMR (376 MHz, $CDCl_3$) δ -61.73.
HRMS (ESI-TOF) m/z: $[M + H]^+$ calculated for $C_{15}H_{24}F_3O_2$ 293.1723; Found 293.1723.

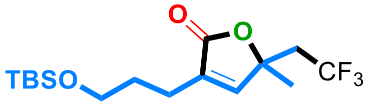

3-(3-((tert-Butyldimethylsilyl)oxy)propyl)-5-methyl-5-(2,2,2-trifluoroethyl)furan-2(5H)-one (4r):
Brown oil, 18.7mg, 53% yield, R_f=0.2 (PE/EtOAc 15/1).
^{1}H NMR (700 MHz, $CDCl_3$) δ 7.00 (s, 1H), 3.59 (td, J = 6.1, 1.5 Hz, 2H), 2.62 – 2.55 (m, 1H), 2.55 – 2.48 (m, 1H), 2.31 (t, J = 7.6 Hz, 2H), 1.74 – 1.70 (m, 2H), 1.49 (s, 3H), 0.85 (s, 9H), -0.00 (s, 6H).
^{13}C NMR (176 MHz, $CDCl_3$) δ 171.9, 149.9, 134.3, 124.7 (q, J = 277.6 Hz), 81.6 (q, J = 1.9 Hz), 62.0, 42.5 (q, J = 28.2 Hz), 30.3, 25.9, 25.0, 21.7, 18.3, -5.4.
^{19}F NMR (376 MHz, $CDCl_3$) δ -61.71.
HRMS (ESI-TOF) m/z: $[M + H]^+$ calculated for $C_{16}H_{28}F_3O_3Si$ 353.1754; Found 353.1754.

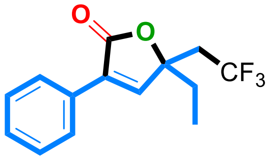

6,6,6-Trifluoro-4-methyl-2-(p-tolyl)hexa-2,3-dienamide (4s):
Light Yellow solid, 11.1mg, 41% yield, R_f=0.2 (PE/EtOAc 15/1).
^{1}H NMR (700 MHz, $CDCl_3$) δ 7.87 – 7.81 (m, 2H), 7.50 – 7.47 (m, 1H), 7.45 – 7.39 (m, 3H), 2.78 – 2.67 (m, 2H), 2.04 (dq, J = 14.7, 7.4 Hz, 1H), 1.91 (dq, J = 14.7, 7.4 Hz, 1H), 0.96 (t, J = 7.4 Hz, 3H).
^{13}C NMR (176 MHz, $CDCl_3$) δ 170.1, 148.0, 132.5, 129.7, 129.0, 128.7, 127.2, 124.8 (q, J = 277.5 Hz), 83.5 (q, J = 2.0 Hz), 41.0 (q, J = 28.1 Hz), 30.9, 7.5.
^{19}F NMR (376 MHz, $CDCl_3$) δ -64.36.
HRMS (ESI-TOF) m/z: $[M + H]^+$ calculated for $C_{14}H_{14}F_3O_2$ 271.0940; Found 271.0938.

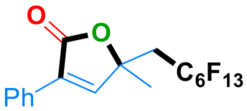

5-Methyl-3-phenyl-5-(2,2,3,3,4,4,5,5,6,6,7,7,7-tridecafluoroheptyl)furan-2(5H)-one (4u):

White solid, 27.4mg, 54% yield, R_f=0.2 (PE/EtOAc 15/1).

^{1}H NMR (700 MHz, $CDCl_3$) δ 7.85 (dd, J = 7.8, 1.5 Hz, 2H), 7.61 (s, 1H), 7.45 – 7.40 (m, 3H), 2.68 (dddd, J = 47.4, 32.2, 15.5, 5.4 Hz, 2H), 1.70 (s, 3H).

^{13}C NMR (176 MHz, $CDCl_3$) δ 169.8, 149.5 (d, J = 2.4 Hz), 131.4, 129.8, 128.9, 128.8, 127.2, 81.6, 39.1 (t, J = 20.3 Hz), 25.6.

^{19}F NMR (376 MHz, $CDCl_3$) δ -80.50 – -86.94 (m), -105.02 – -115.79 (m), -121.66 (d, J = 11.0 Hz), -122.84, -123.41 (dt, J = 17.6, 8.8 Hz), -125.55 – -126.37 (m).

HRMS (ESI-TOF) m/z: $[M + H]^+$ calculated for $C_{18}H_{12}F_{13}O_2$ 507.0624; Found 507.0620.

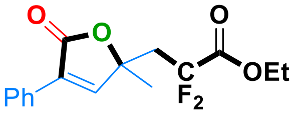

Ethyl-2,2-difluoro-3-(2-methyl-5-oxo-4-phenyl-2,5-dihydrofuran-2-yl)propanoate (4v):

Yellow solid, 19.2mg, 62% yield, R_f=0.2 (PE/EtOAc 15/1).

^{1}H NMR (700 MHz, $CDCl_3$) δ 7.86 – 7.80 (m, 2H), 7.54 (s, 1H), 7.44 – 7.38 (m, 3H), 4.31 (qt, J = 8.6, 4.3 Hz, 2H), 2.79 – 2.64 (m, 2H), 1.63 (s, 3H), 1.32 (t, J = 7.2 Hz, 3H).

^{13}C NMR (176 MHz, $CDCl_3$) δ 170.0, 163.3 (t, J = 31.7 Hz), 150.1, 131.0, 129.6, 129.1, 128.7, 127.2, 114.1 (t, J = 252.2 Hz), 81.5, 63.6, 42.9 (t, J = 23.0 Hz), 25.6, 13.8.

^{19}F NMR (376 MHz, $CDCl_3$) δ-101.22, -101.93, -102.29, -103.00.

HRMS (ESI-TOF) m/z: $[M + H]^+$ calculated for $C_{16}H_{17}F_2O_4$ 311.1089; Found 311.1089.

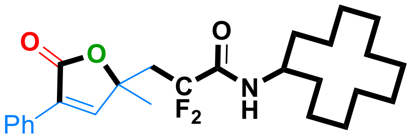

N-cyclododecyl-2,2-difluoro-3-(2-methyl-5-oxo-4-phenyl-2,5-dihydrofuran-2-yl)propanamide (4w):

Yellow solid, 22.0mg, 49% yield, R_f=0.2 (PE/EtOAc 5/1).

^{1}H NMR (700 MHz, $CDCl_3$) δ 7.87 – 7.82 (m, 2H), 7.56 (s, 1H), 7.43 – 7.36 (m, 3H), 6.10 (d, J = 7.7 Hz, 1H), 3.96 – 3.88 (m, 1H), 2.88 (dt, J = 19.0, 15.9 Hz, 1H), 2.78 – 2.67 (m, 1H), 1.62 (s, 3H), 1.42 – 1.26 (m, 20H), 1.22 – 1.20 (m, 2H).

^{13}C NMR (176 MHz, $CDCl_3$) δ 170.3, 162.6 (t, J = 27.7 Hz), 150.2, 130.7, 129.6, 129.2, 128.7, 127.1, 116.3 (t, 256.5), 81.8 (d, J = 4.9 Hz), 47.1, 41.2 (t, J = 22.8 Hz), 29.5, 29.3, 26.0, 24.0, 23.9, 23.9, 23.4, 23.2, 23.4, 23.0, 21.0, 20.9.

^{19}F NMR (376 MHz, $CDCl_3$) δ -99.72 (d, J = 266.0 Hz), -105.25 (d, J = 265.9 Hz).

HRMS (ESI-TOF) m/z: $[M + H]^+$ calculated for $C_{26}H_{36}F_2NO_3$ 448.2658; Found 448.2658.

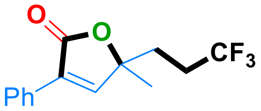

5-Methyl-3-phenyl-5-(3,3,3-trifluoropropyl)furan-2(5H)-one (4x):

Yellow solid, 14.4mg, 53% yield, R_f=0.2 (PE/EtOAc 15/1).

^{1}H NMR (700 MHz, $CDCl_3$) δ 7.88 – 7.83 (m, 2H), 7.46 – 7.44 (m, 1H), 7.44 – 7.39 (m, 3H),

2.23 – 2.16 (m, 2H), 2.09 – 1.98 (m, 2H), 1.58 (s, 3H).

^{13}C NMR (176 MHz, $CDCl_3$) δ 170.5, 150.4, 131.7, 129.7, 129.0, 128.8, 127.2, 126.7 (q, J = 276.0 Hz), 83.8, 30.8 (q, J = 2.8 Hz), 28.7 (q, J = 29.7 Hz), 24.4.

^{19}F NMR (376 MHz, $CDCl_3$) δ -66.39.

HRMS (ESI-TOF) m/z: $[M + H]^+$ calculated for $C_{14}H_{14}F_3O_2$ 271.0940; Found 271.0942.

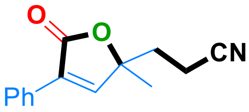

3-(2-Methyl-5-oxo-4-phenyl-2,5-dihydrofuran-2-yl)propanenitrile (4y):

Yellow solid, 8.6mg, 38% yield, R_f=0.2 (PE/EtOAc 5/1).

^{1}H NMR (700 MHz, $CDCl_3$) δ 7.90 – 7.82 (m, 2H), 7.50 (s, 1H), 7.45 – 7.39 (m, 3H), 2.47 – 2.17 (m, 4H), 1.61 (s, 3H).

^{13}C NMR (176 MHz, $CDCl_3$) δ 170.4, 149.8, 133.0, 129.8, 128.9, 128.8, 127.3, 119.0, 83.8, 34.2, 24.5, 12.1.

HRMS (ESI-TOF) m/z: $[M + H]^+$ calculated for $C_{14}H_{14}NO_2$ 228.1019; Found 228.1018.

7. NMR Spectra of the Products.

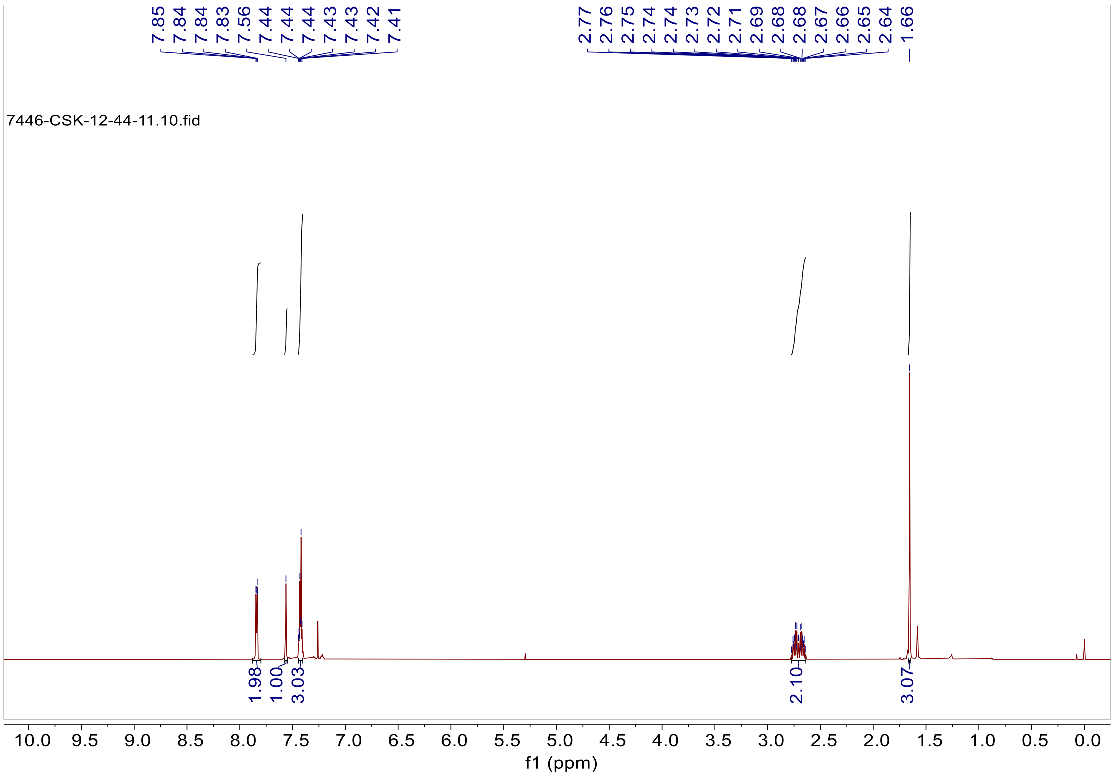

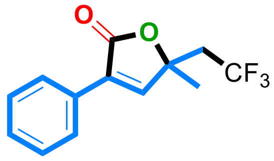

4a

^{1}H NMR (700 MHz, $CDCl_3$)
^{13}C NMR (176 MHz, $CDCl_3$)

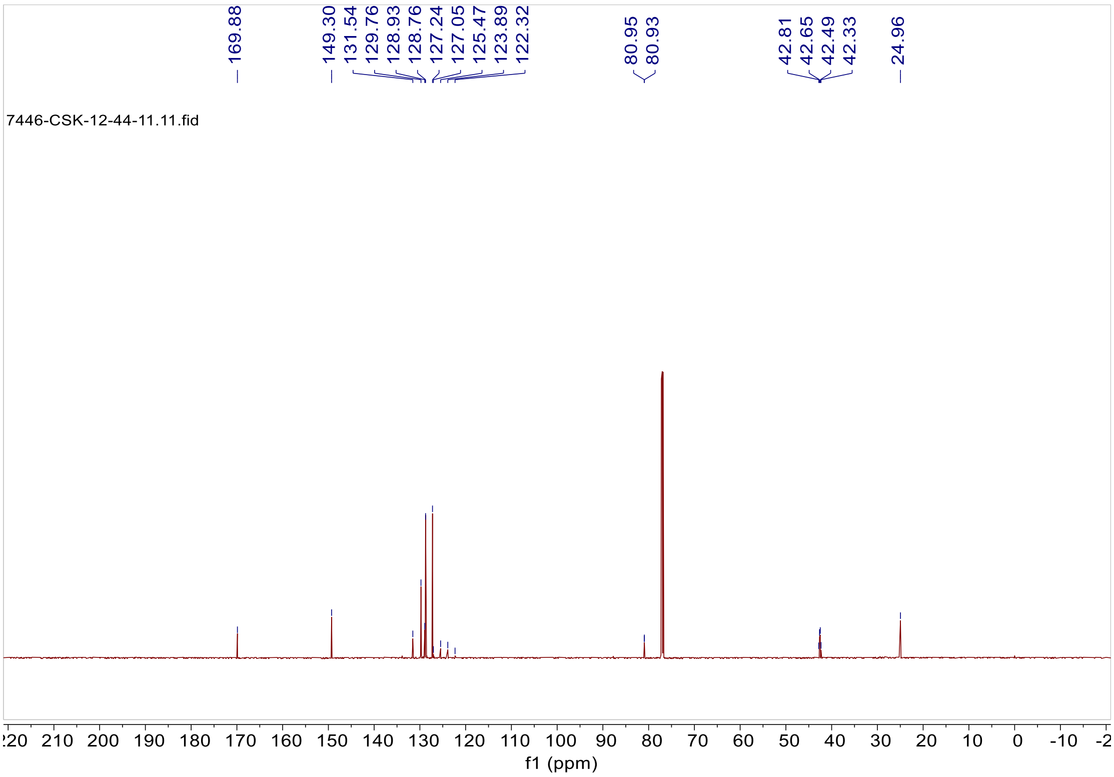

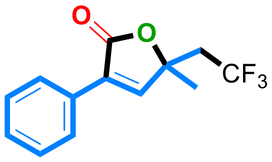

4a

^{19}F NMR (376 MHz, $CDCl_3$)

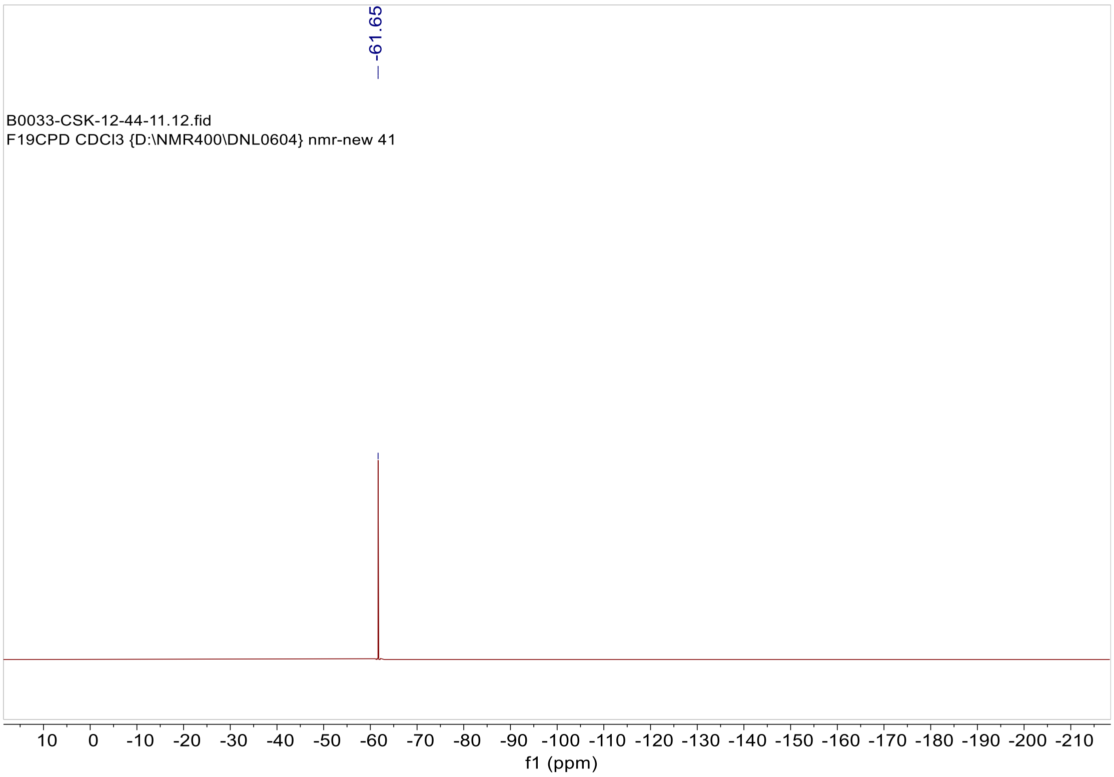

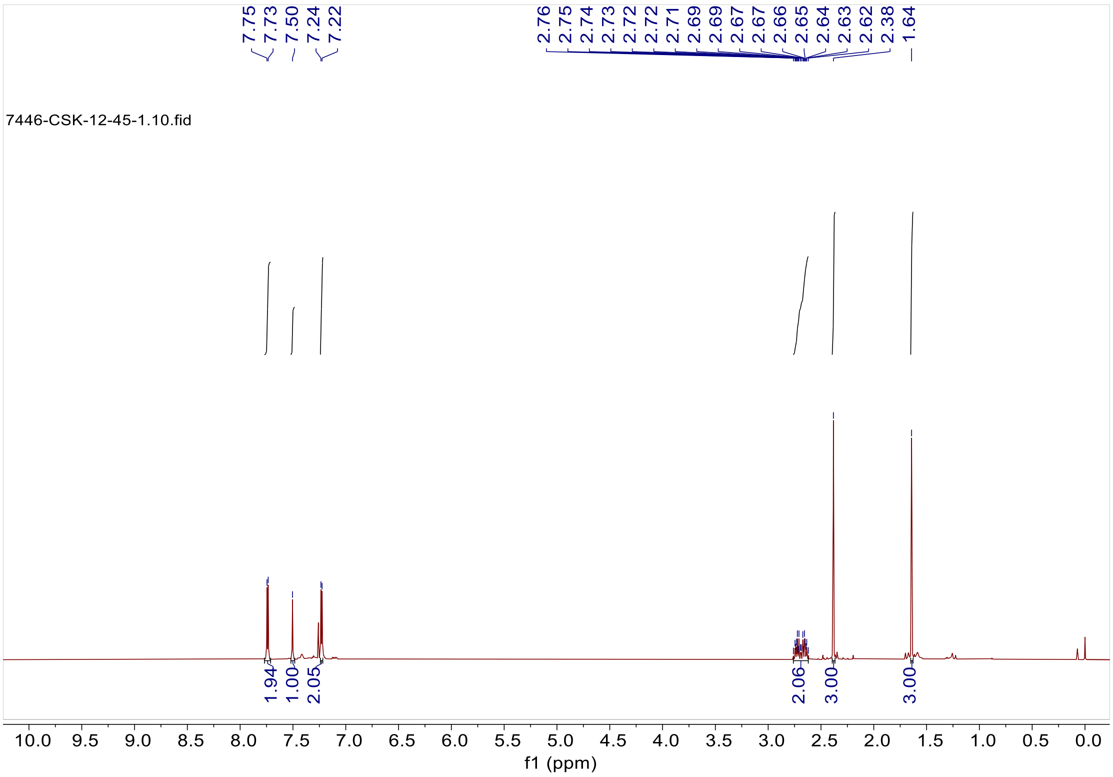

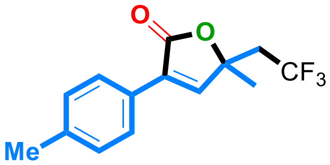

4b

^{1}H NMR (700 MHz, $CDCl_3$)
^{13}C NMR (176 MHz, $CDCl_3$)

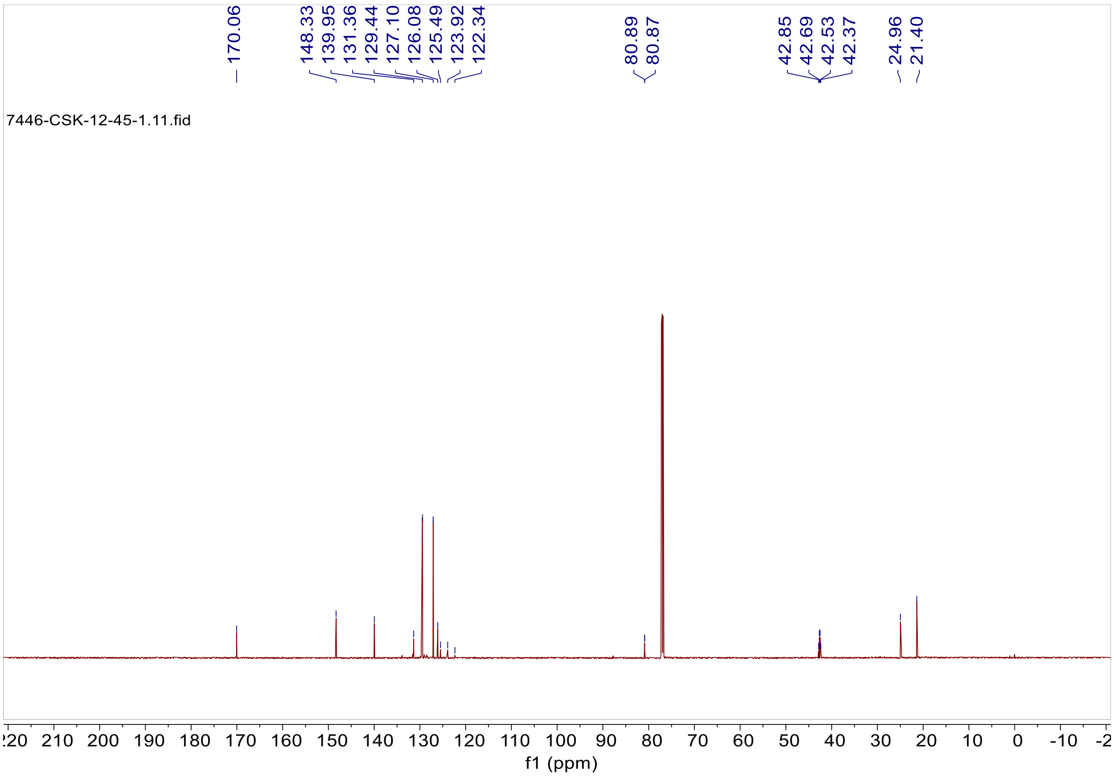

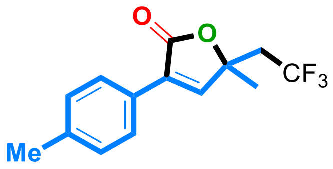

4b

^{19}F NMR (376 MHz, $CDCl_3$)

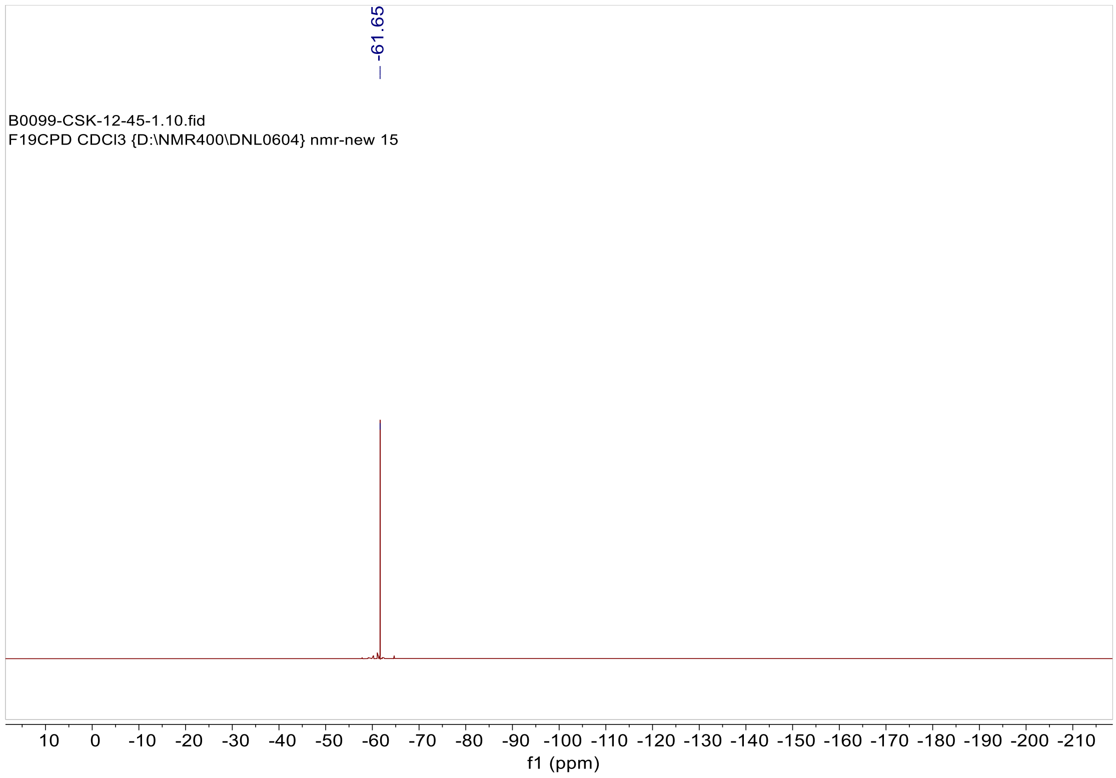

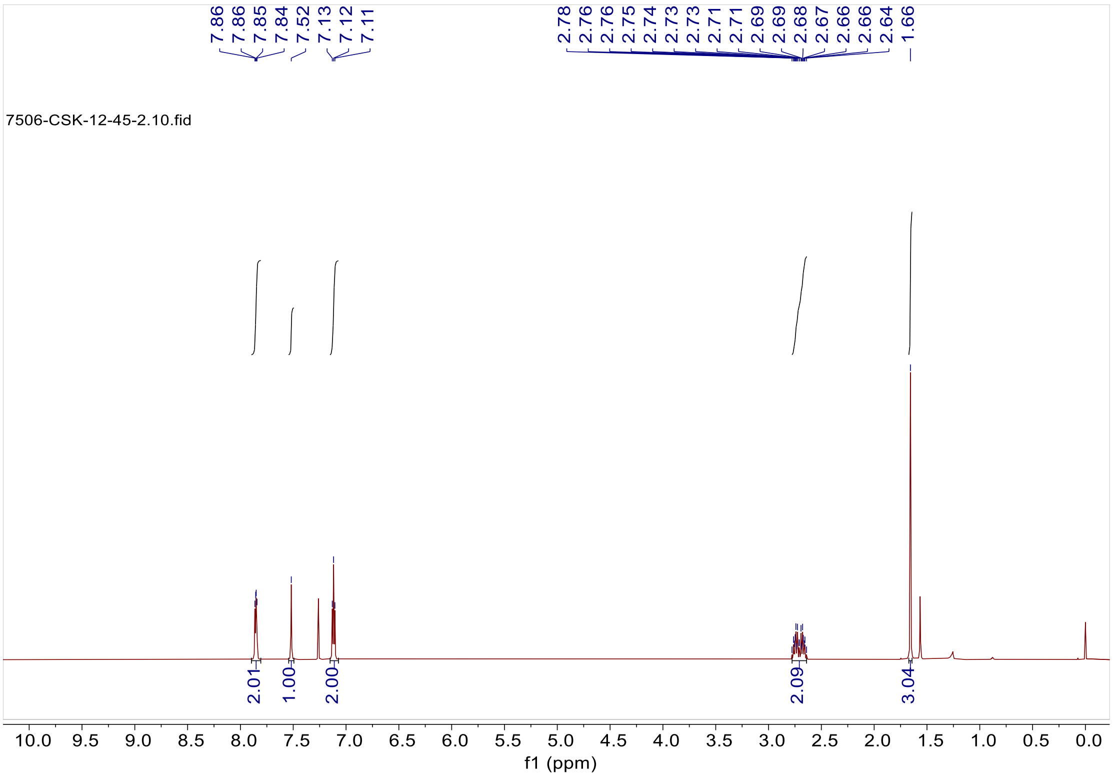

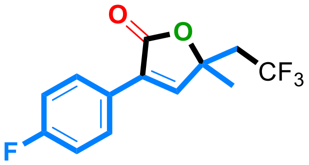

4c

^{1}H NMR (700 MHz, $CDCl_3$)
^{13}C NMR (176 MHz, $CDCl_3$)

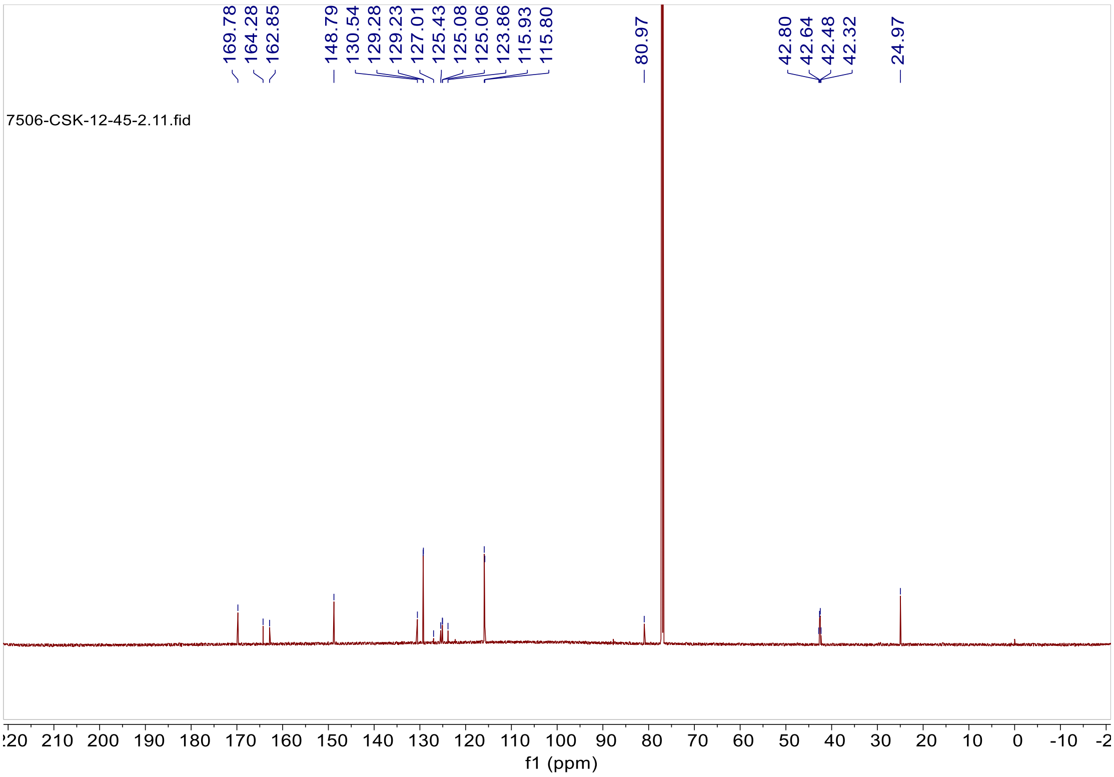

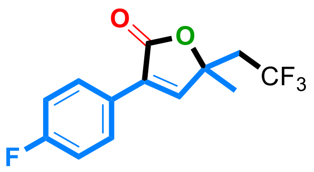

4c

^{19}F NMR (376 MHz, $CDCl_3$)

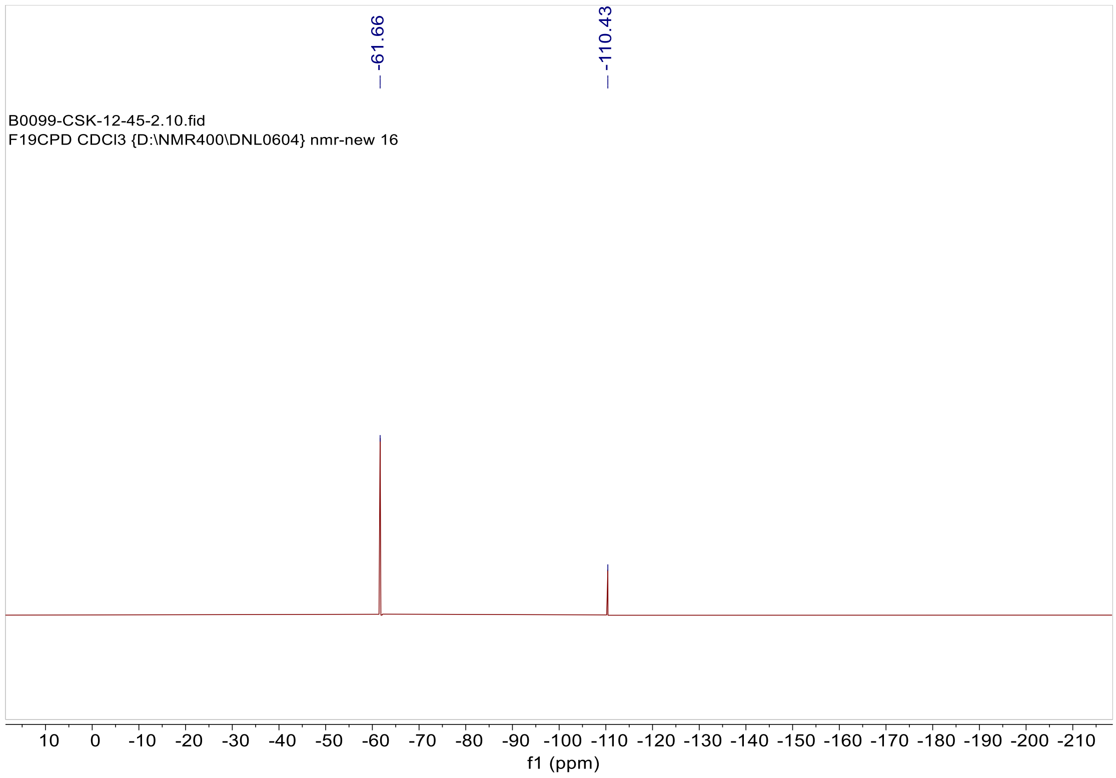

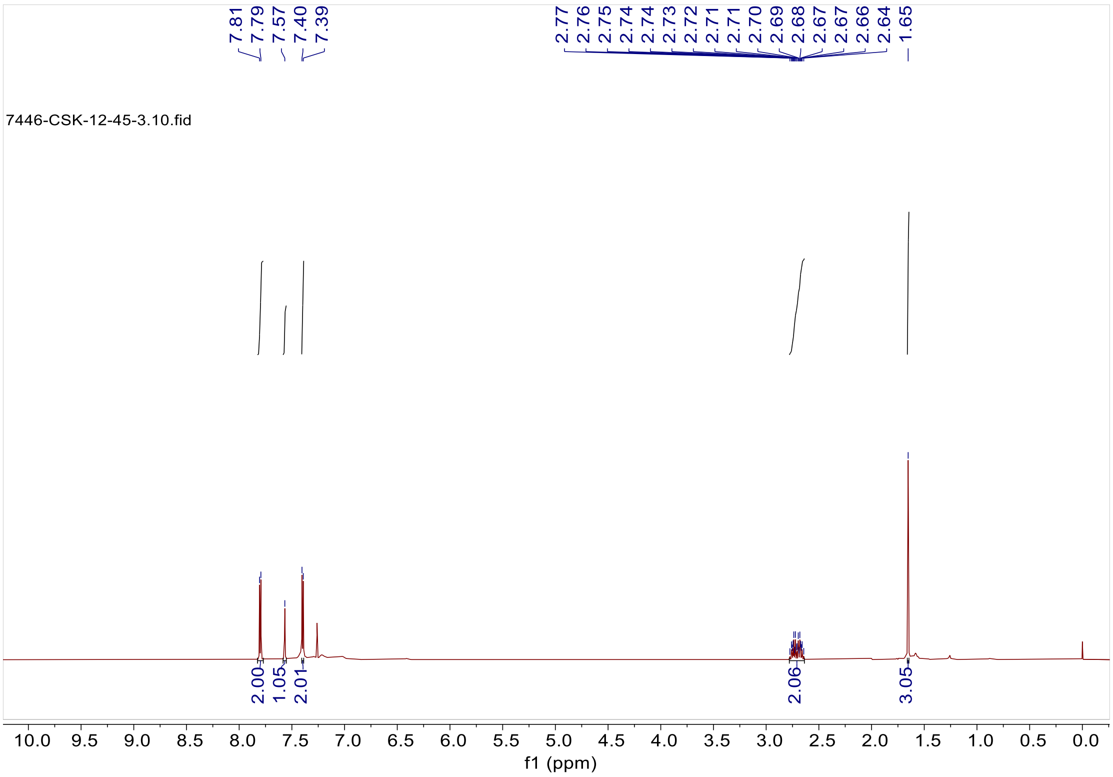

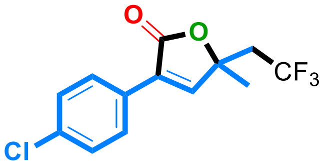

4d

^{1}H NMR (700 MHz, $CDCl_3$)
^{13}C NMR (176 MHz, $CDCl_3$)

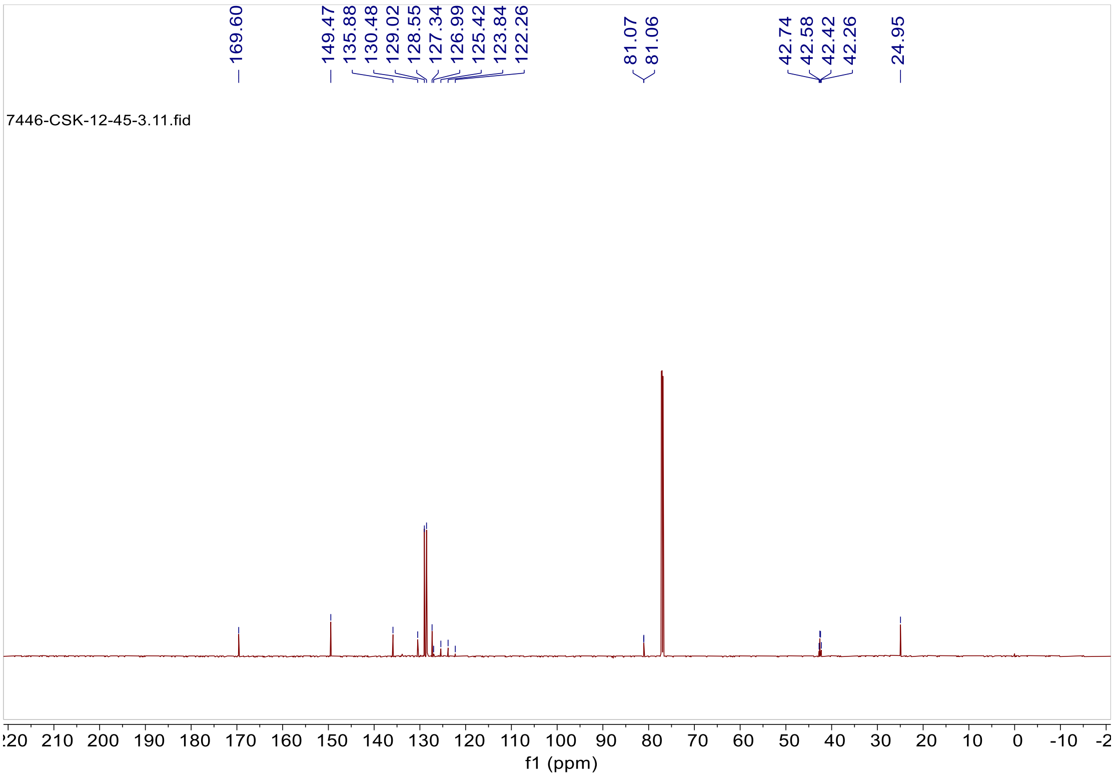

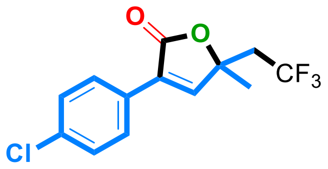

4d

^{19}F NMR (376 MHz, $CDCl_3$)

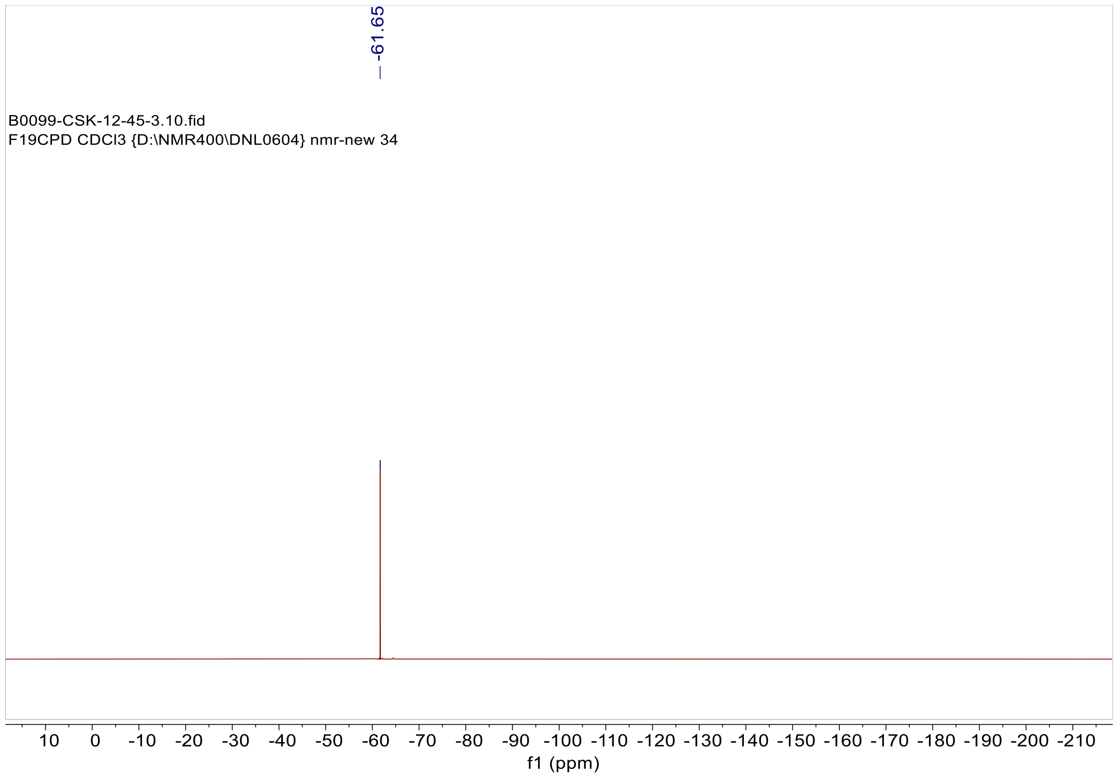

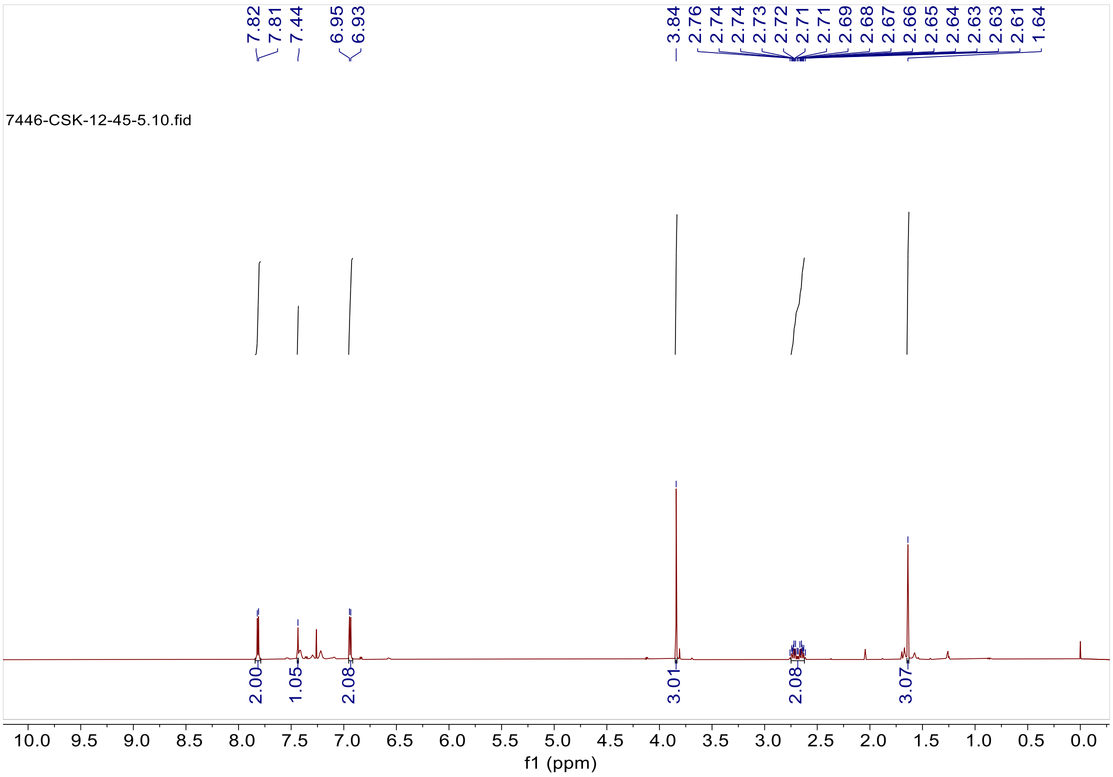

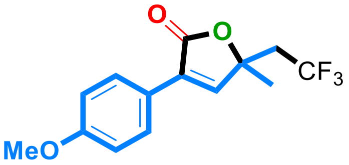

4f

^{1}H NMR (700 MHz, $CDCl_3$)
^{13}C NMR (176 MHz, $CDCl_3$)

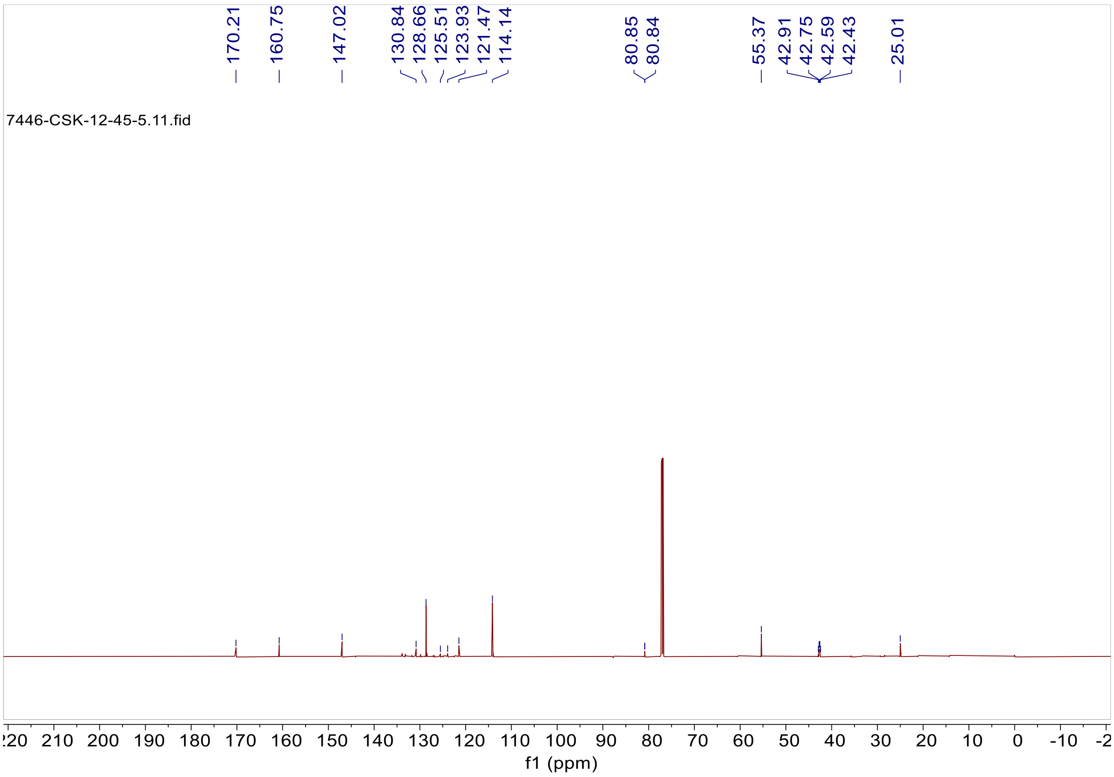

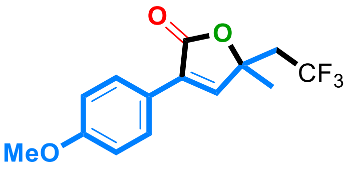

4f

^{19}F NMR (376 MHz, $CDCl_3$)

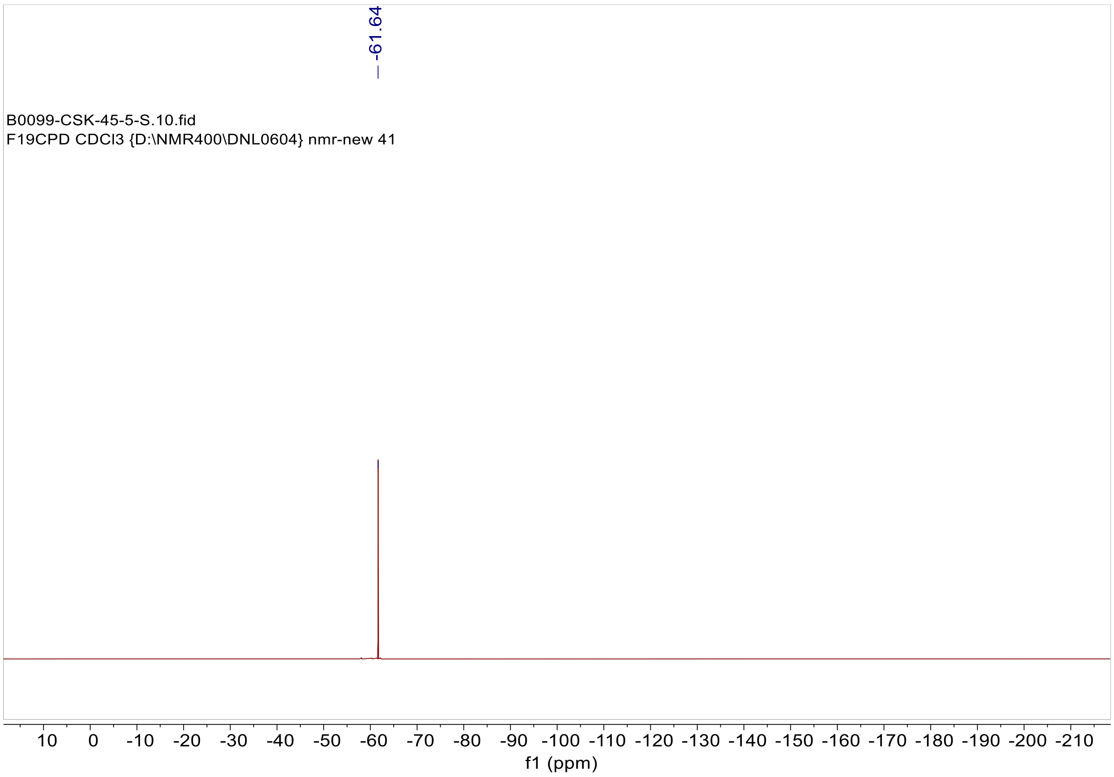

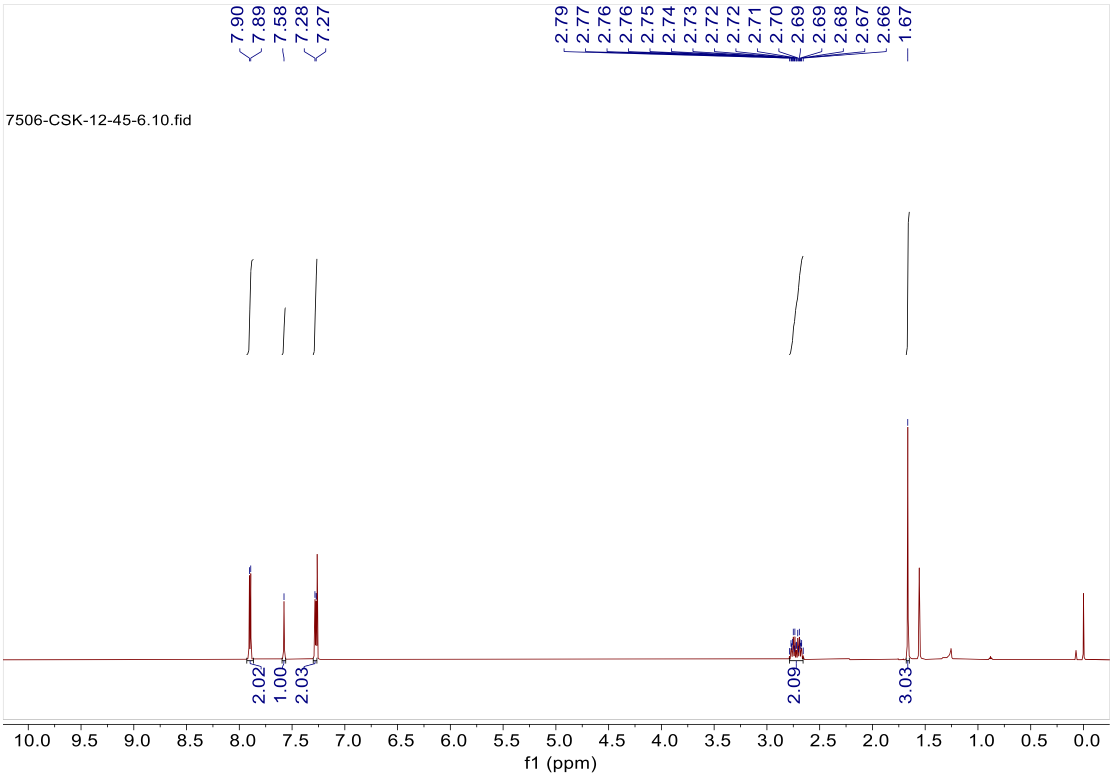

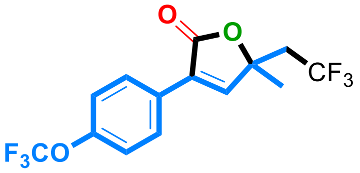

4g

^{1}H NMR (700 MHz, $CDCl_3$)

^{13}C NMR (176 MHz, $CDCl_3$)

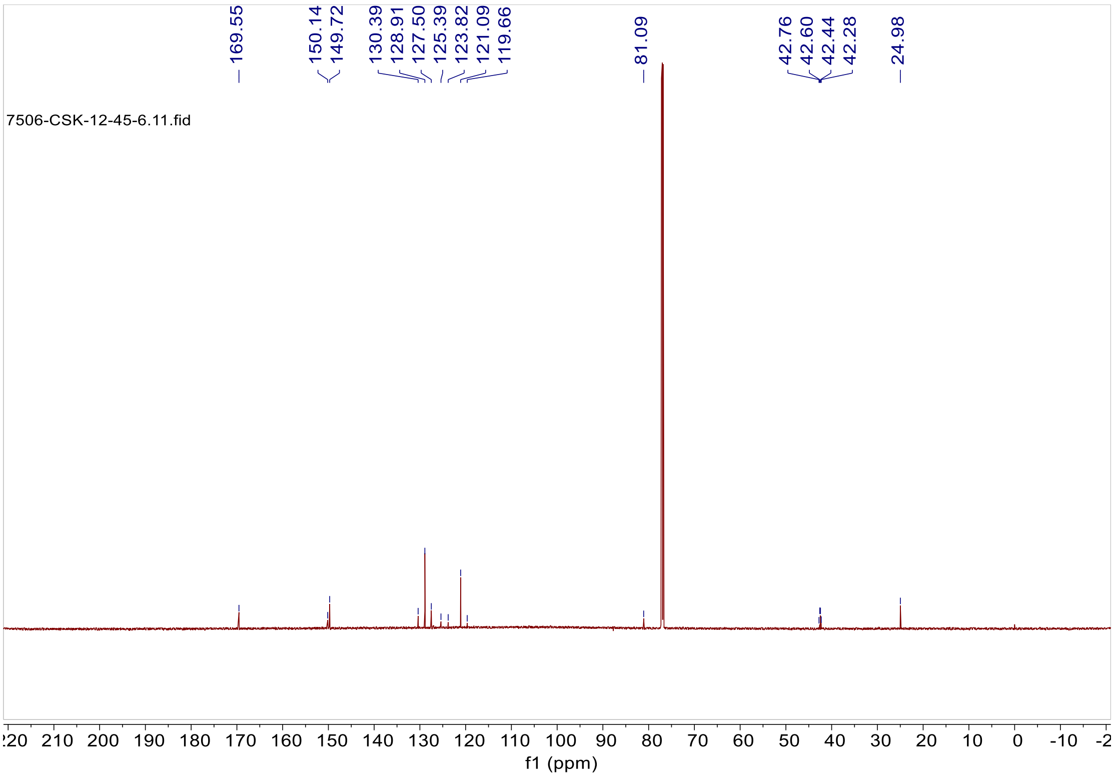

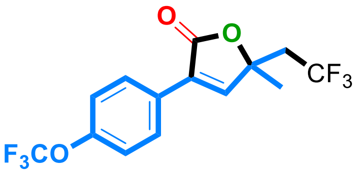

4g

^{19}F NMR (376 MHz, $CDCl_3$)

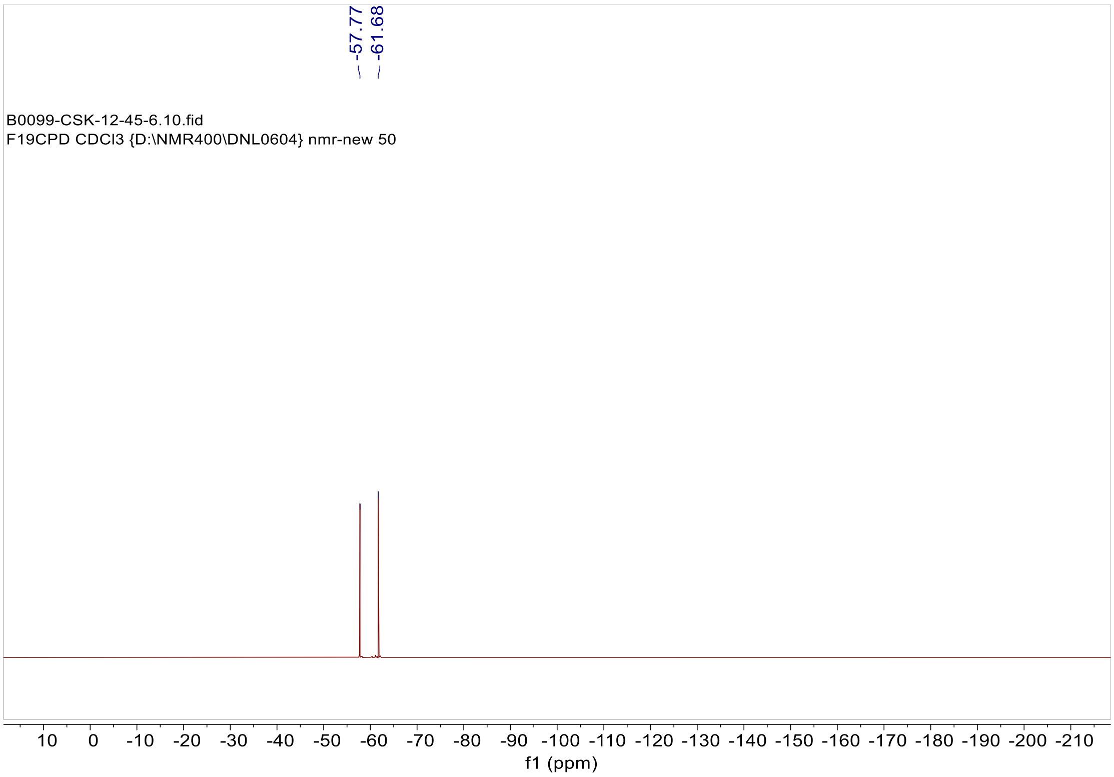

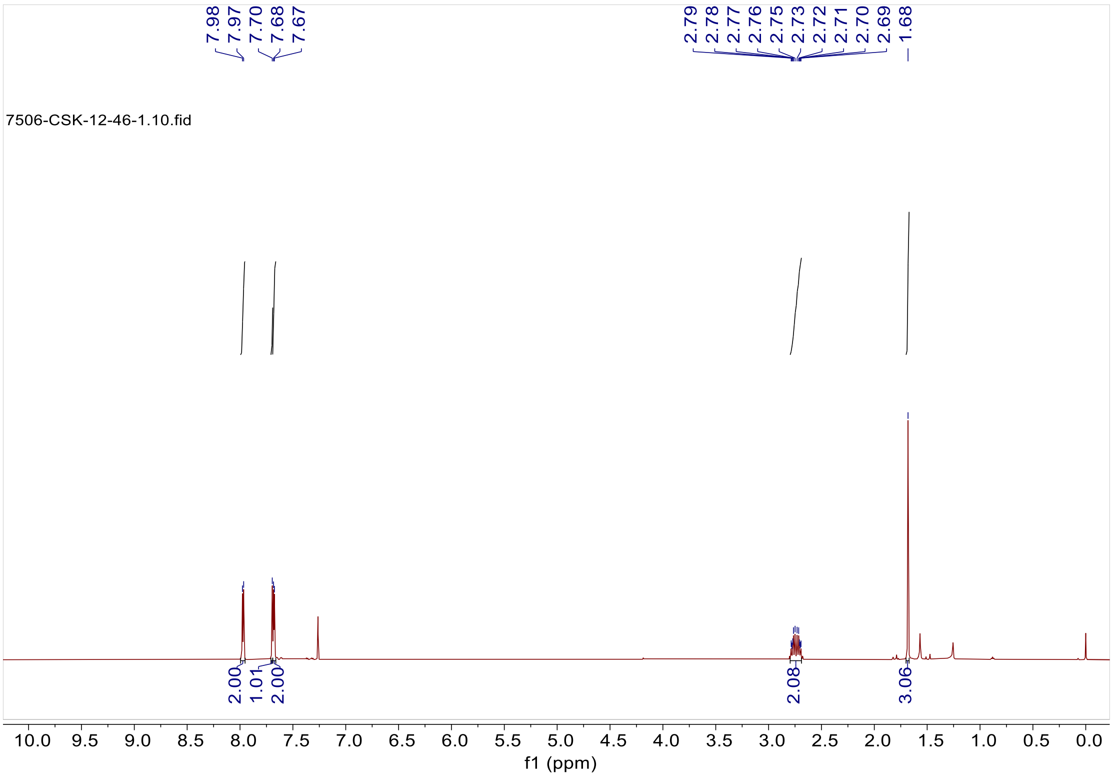

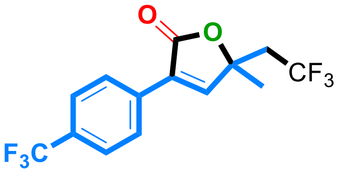

4h

^{1}H NMR (700 MHz, $CDCl_3$)
^{13}C NMR (176 MHz, $CDCl_3$)

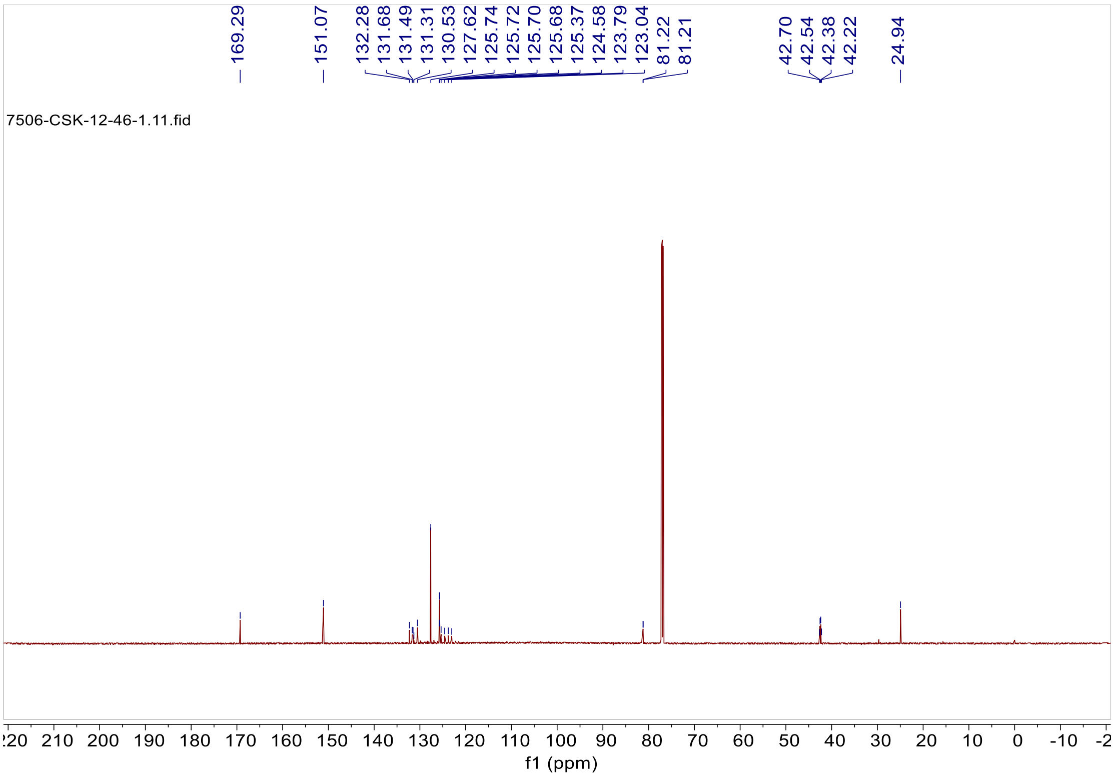

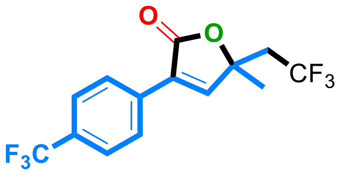

4h

^{19}F NMR (376 MHz, $CDCl_3$)

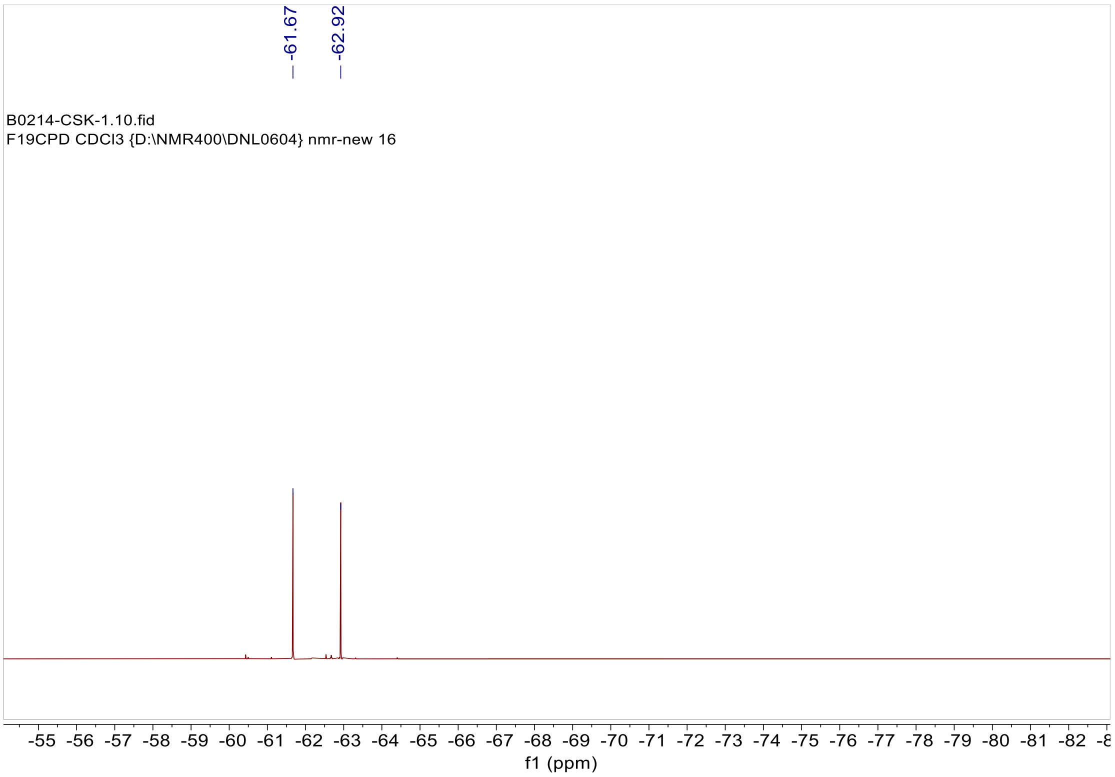

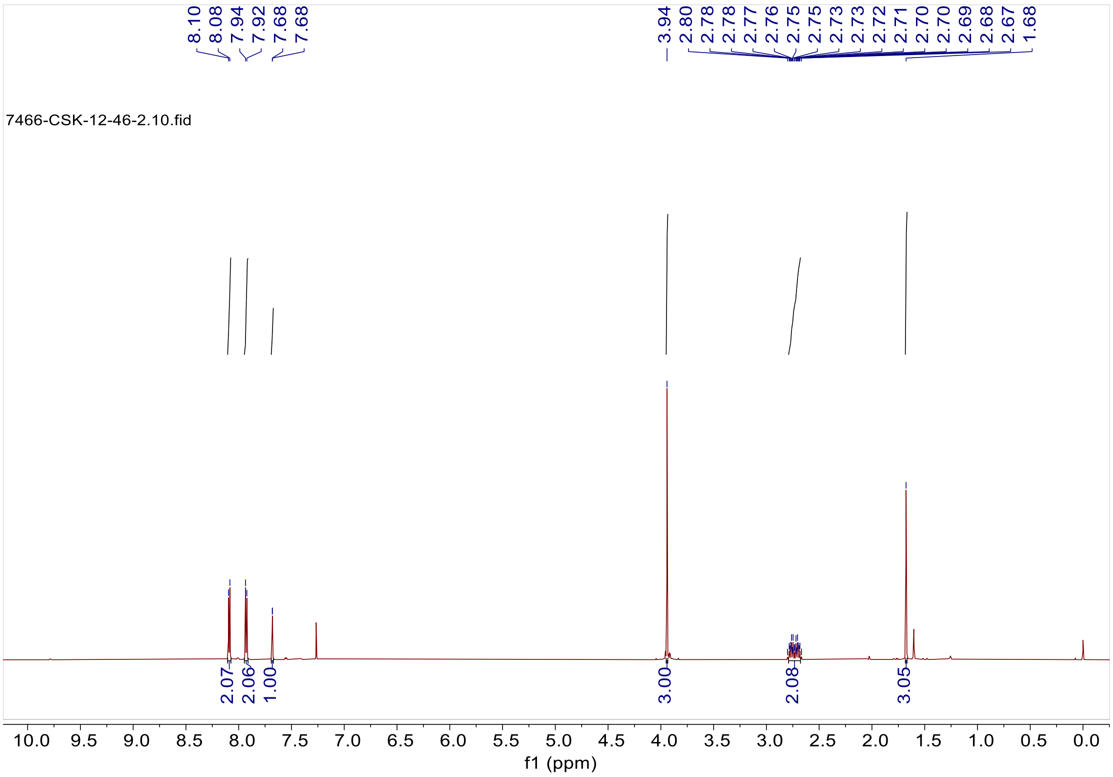

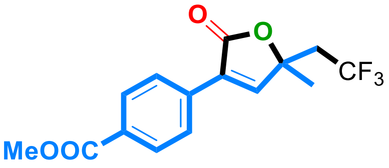

4i

^{1}H NMR (700 MHz, $CDCl_3$)
^{13}C NMR (176 MHz, $CDCl_3$)

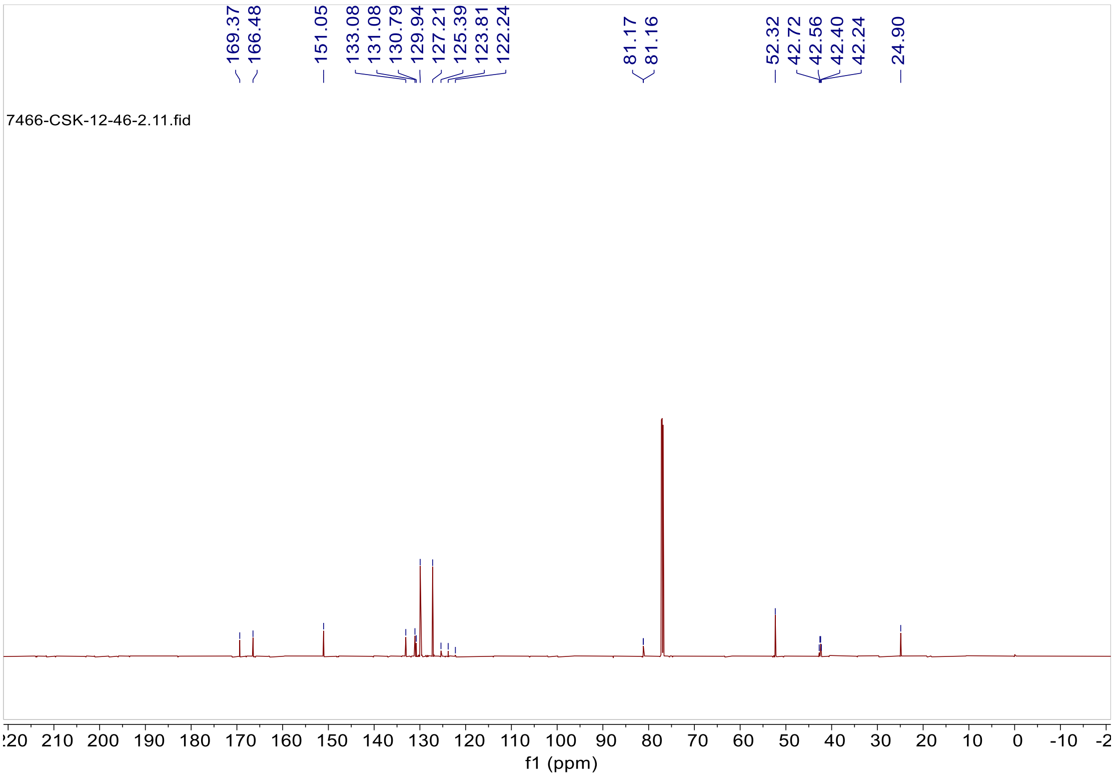

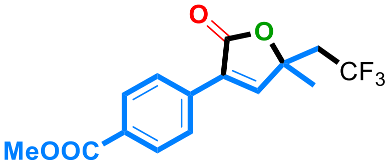

4i

^{19}F NMR (376 MHz, $CDCl_3$)

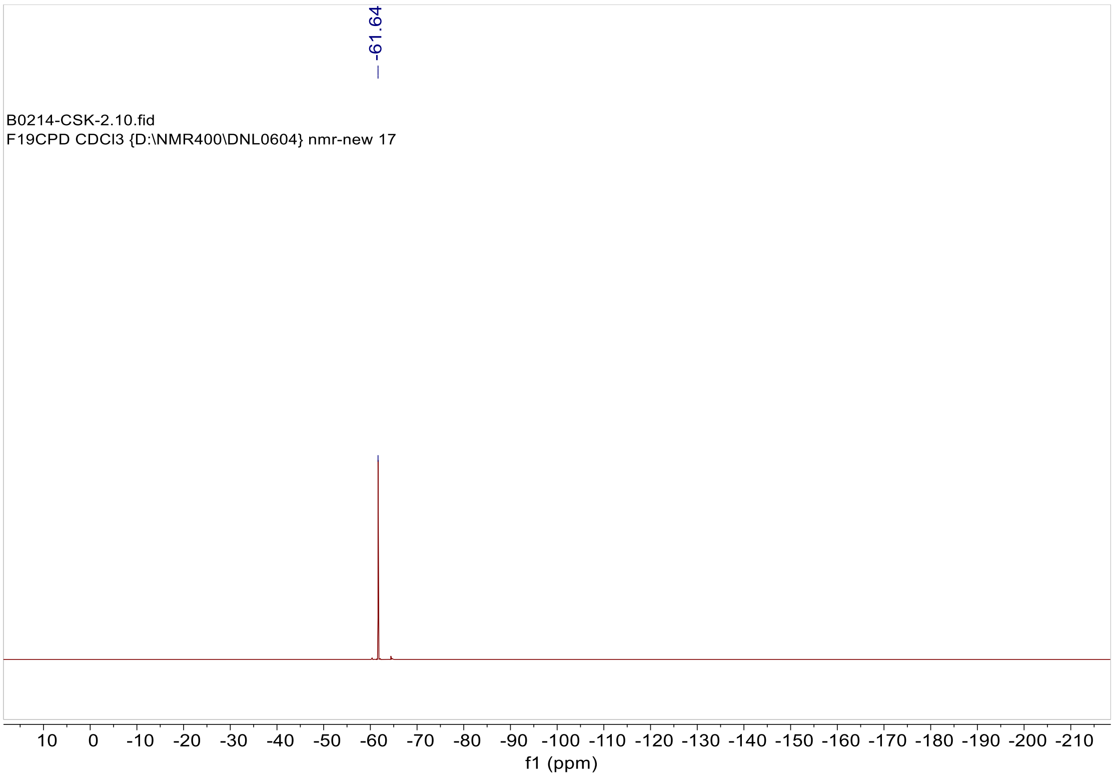

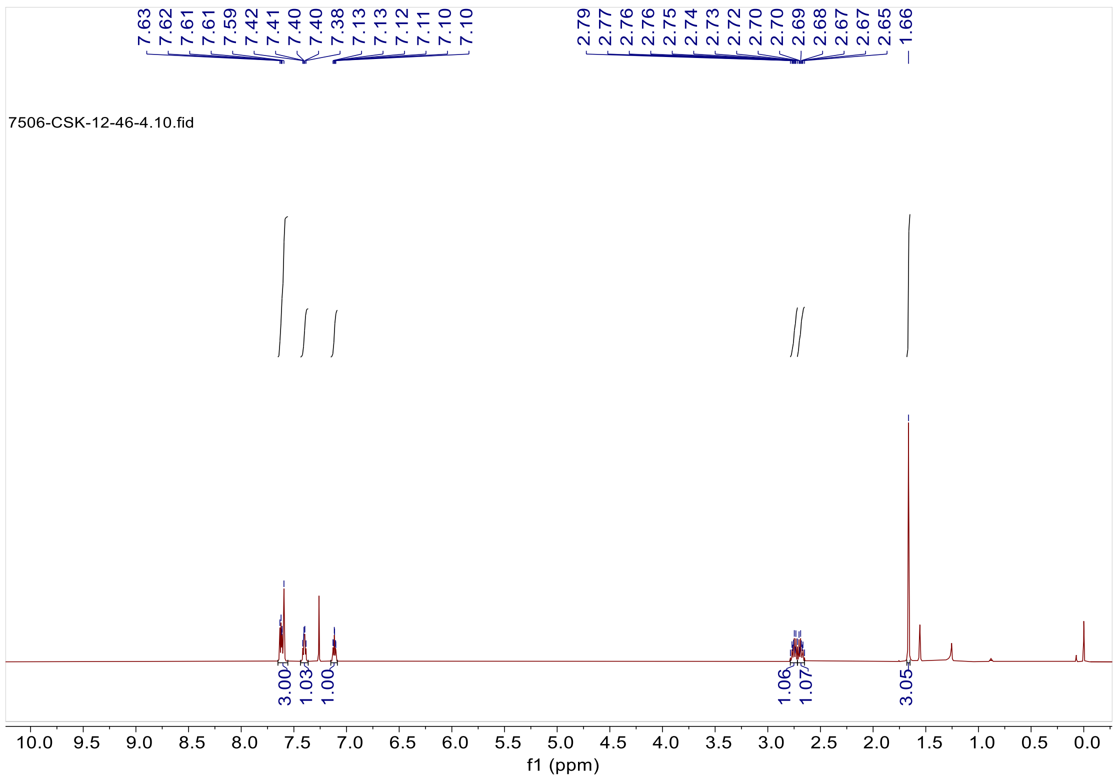

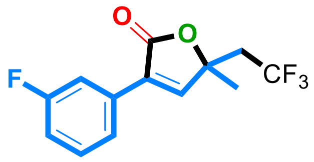

4l

^{1}H NMR (700 MHz, $CDCl_3$)

^{13}C NMR (176 MHz, $CDCl_3$)

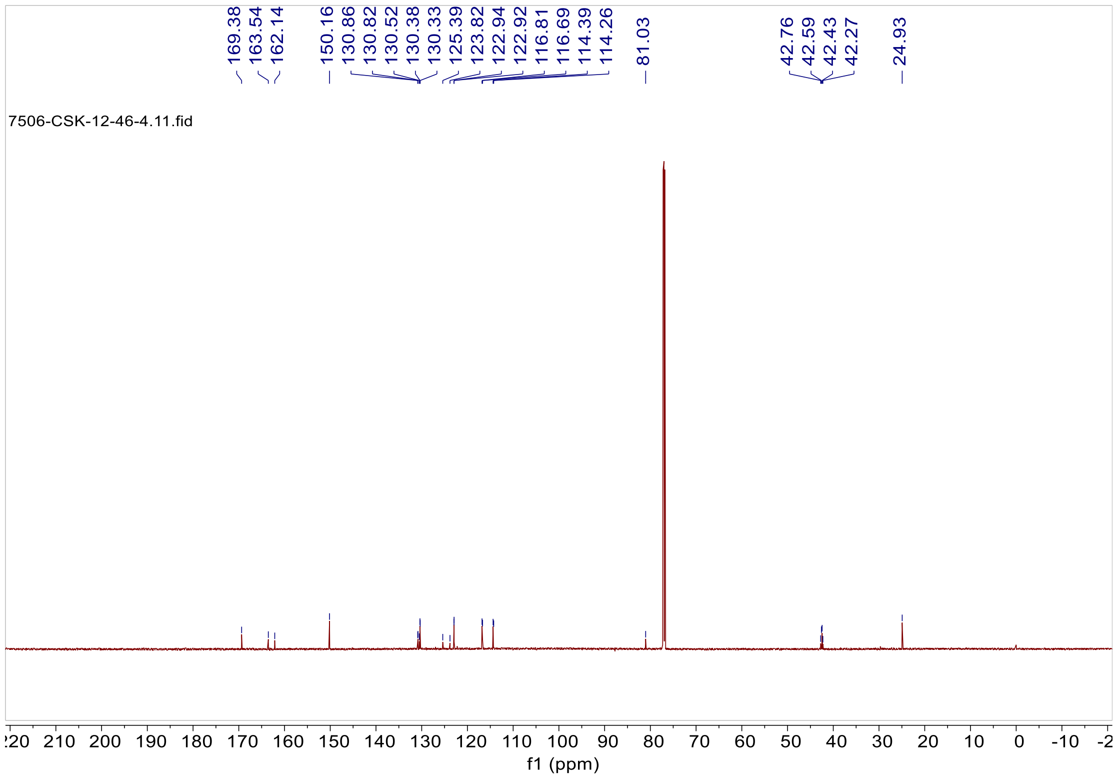

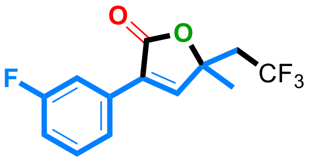

4l

^{19}F NMR (376 MHz, $CDCl_3$)

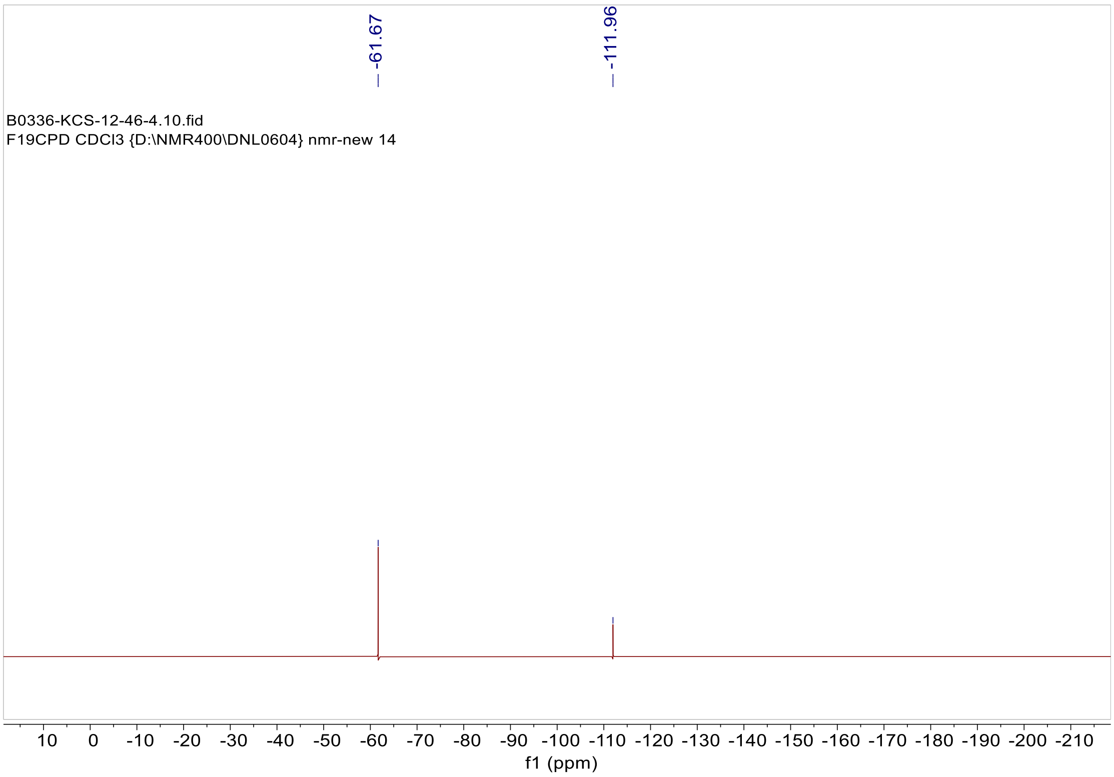

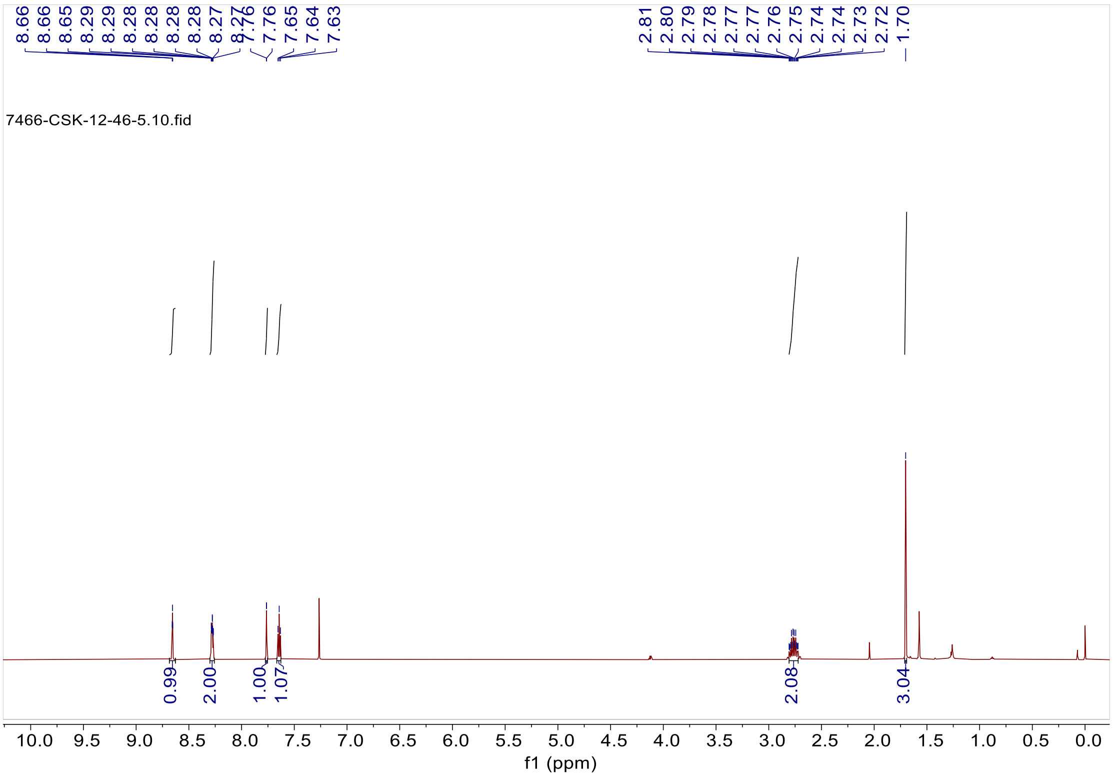

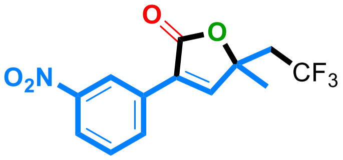

4m

^{1}H NMR (700 MHz, $CDCl_3$)
^{13}C NMR (176 MHz, $CDCl_3$)

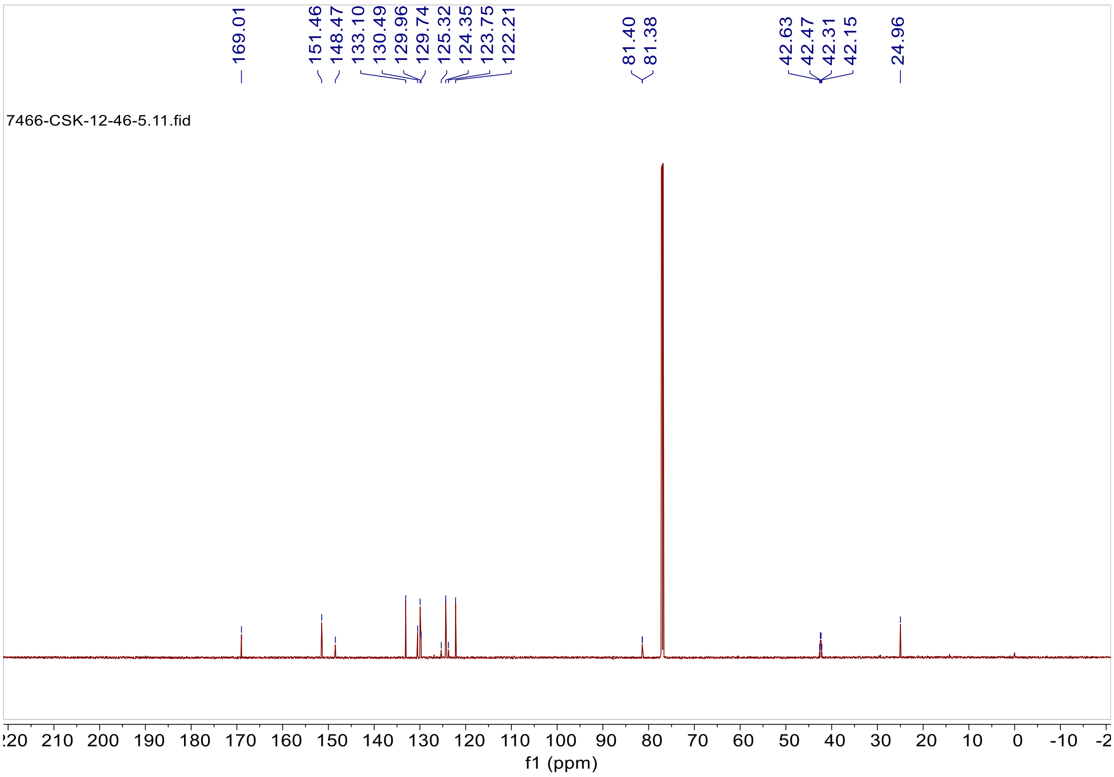

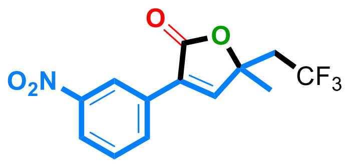

4m

^{19}F NMR (376 MHz, $CDCl_3$)

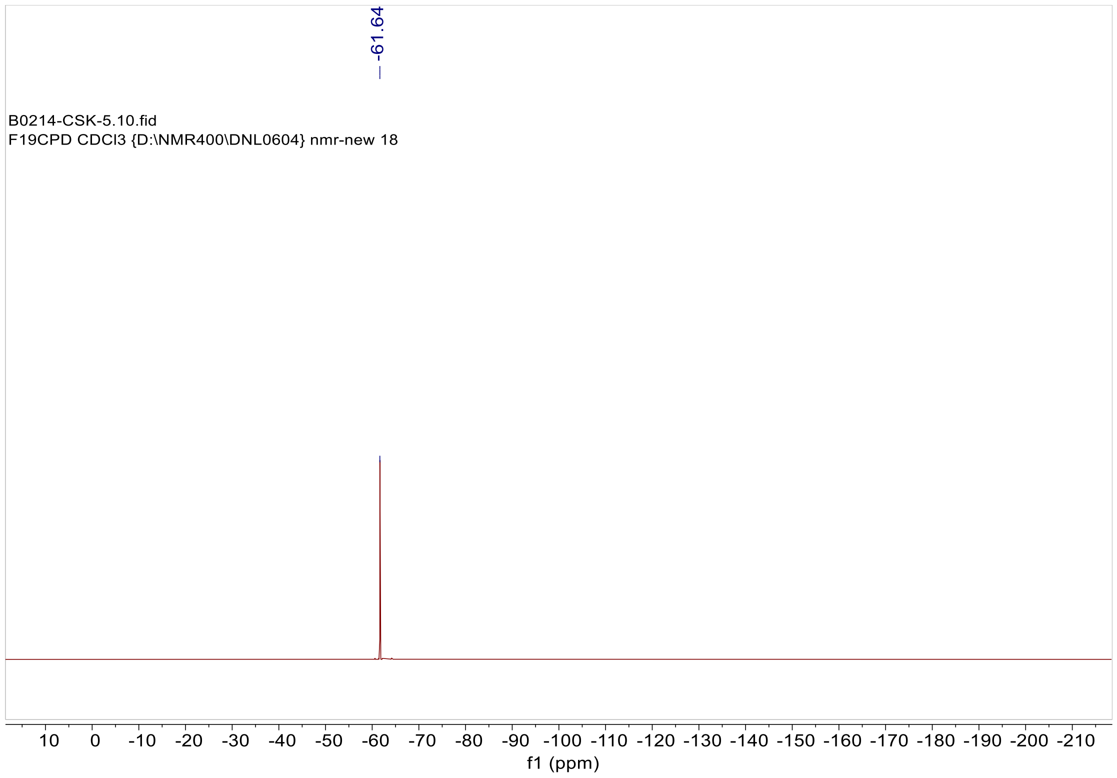

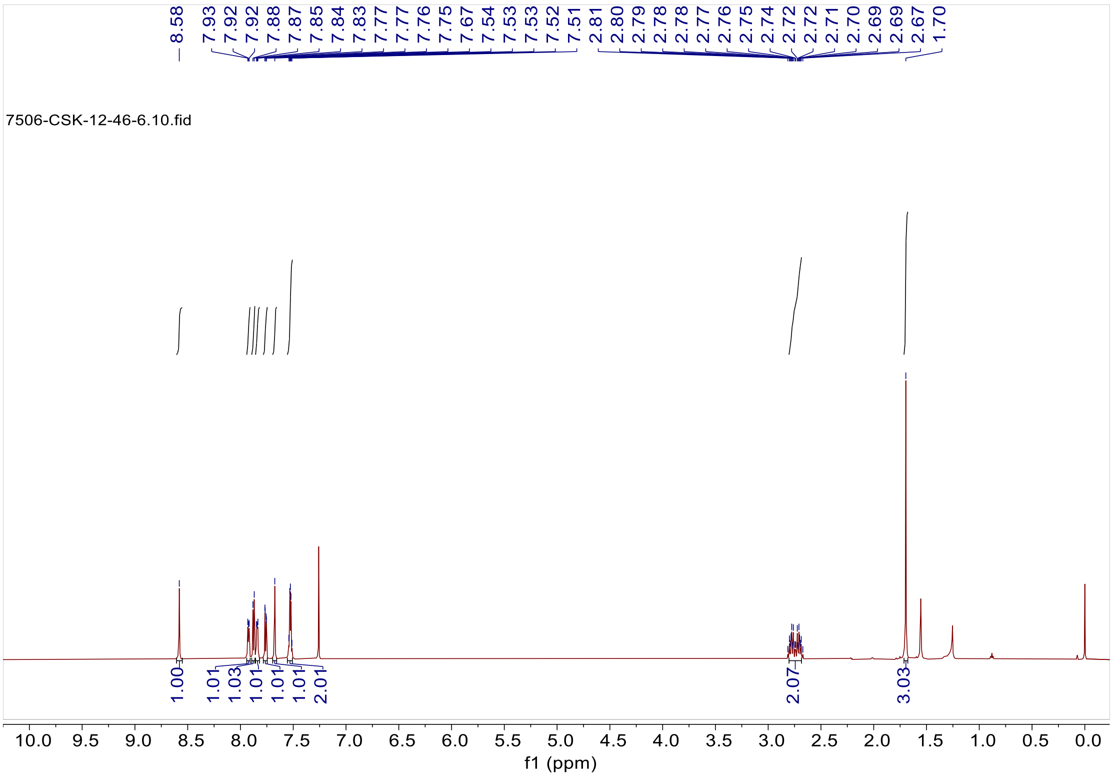

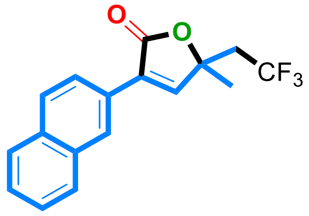

4n

^{1}H NMR (700 MHz, $CDCl_3$)
^{13}C NMR (176 MHz, $CDCl_3$)

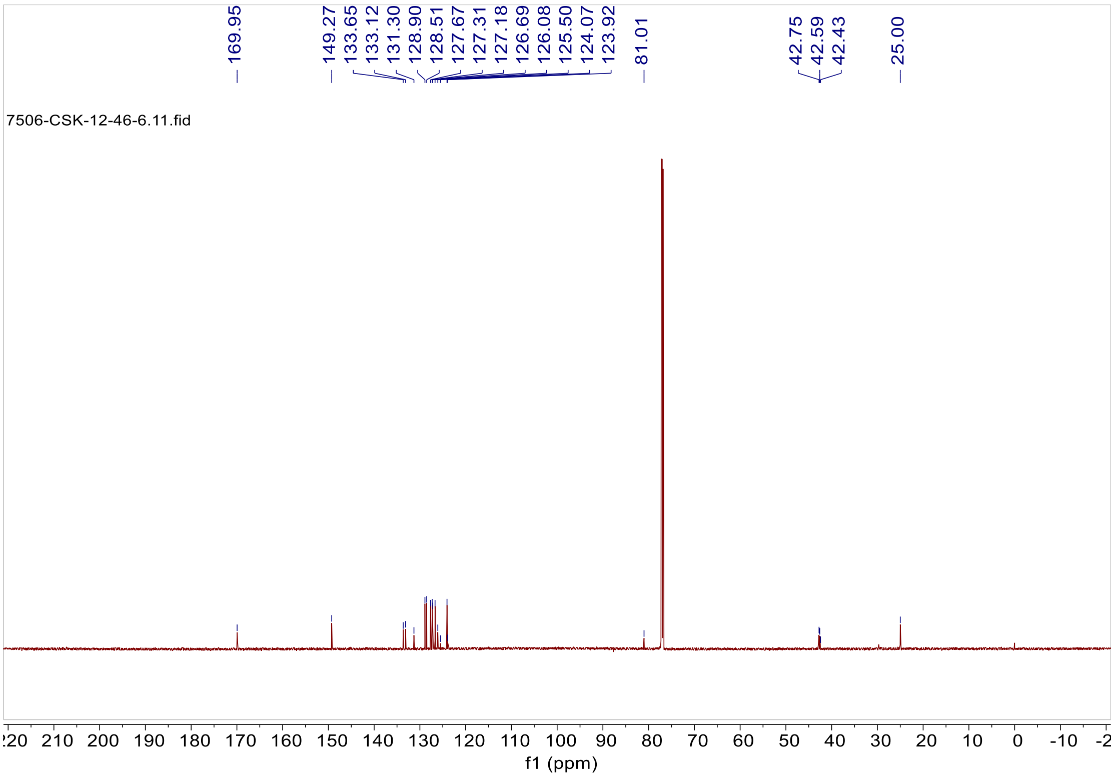

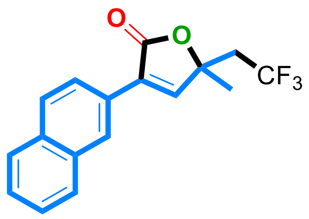

4n

^{19}F NMR (376 MHz, $CDCl_3$)

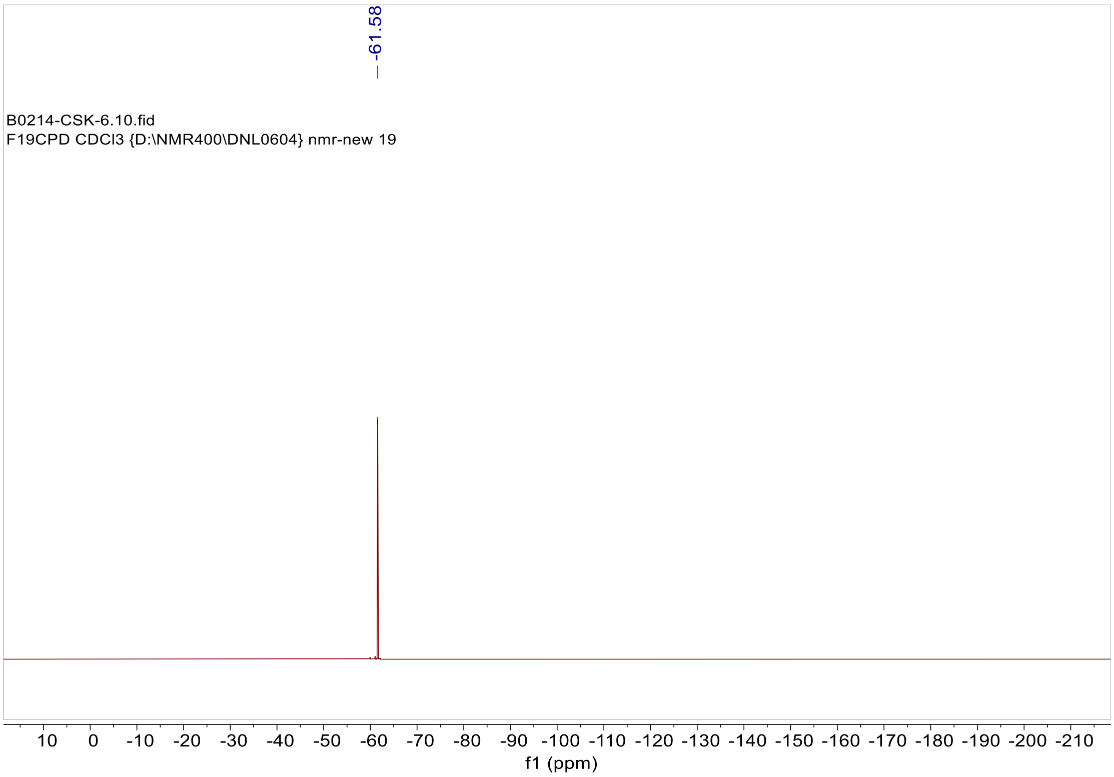

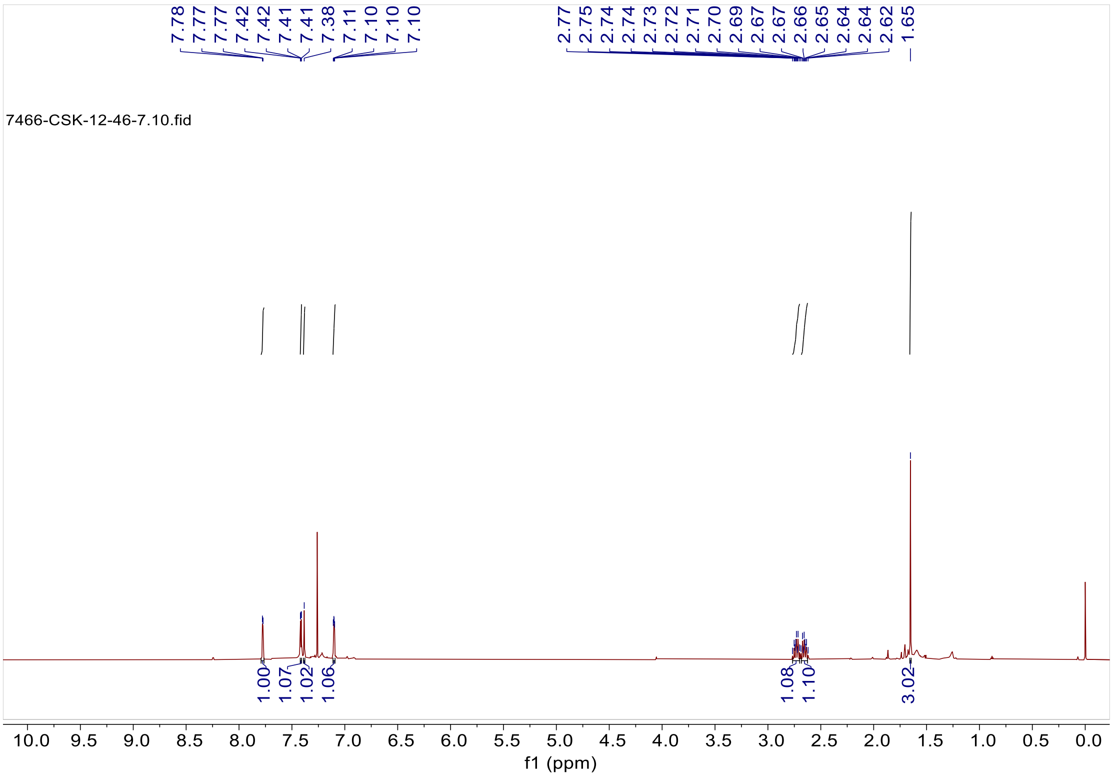

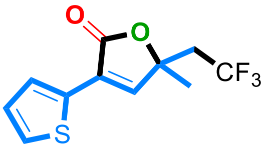

4o

^{1}H NMR (700 MHz, $CDCl_3$)

^{13}C NMR (176 MHz, $CDCl_3$)

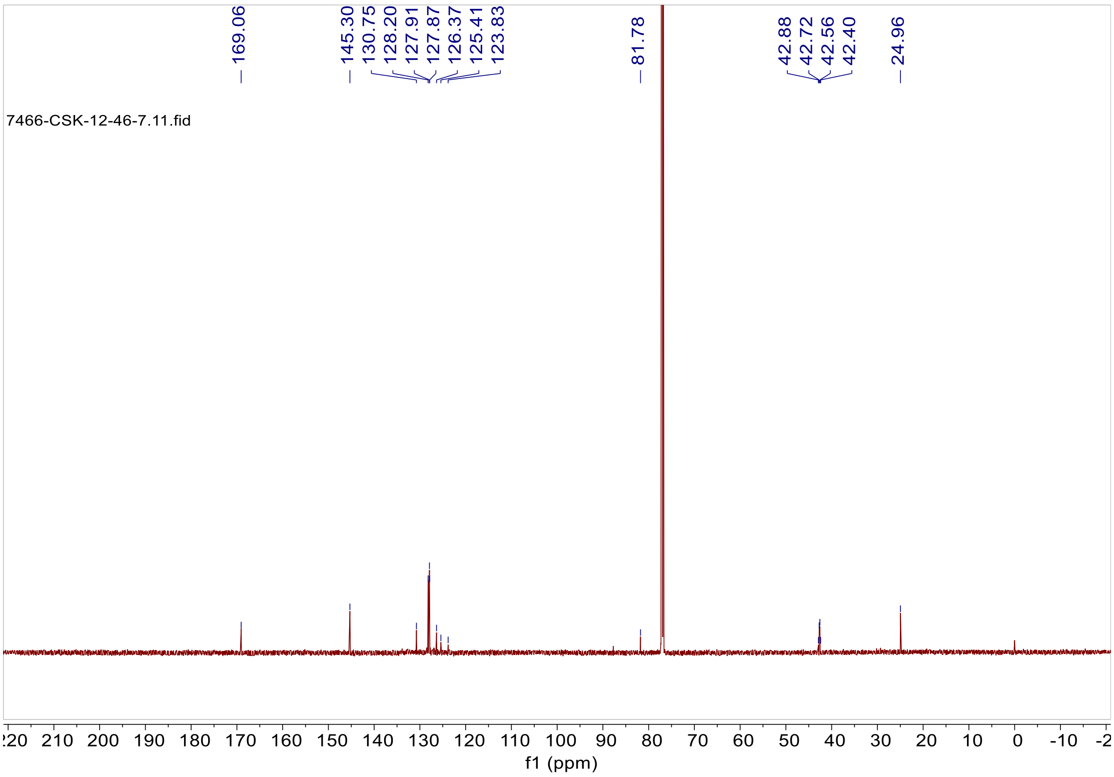

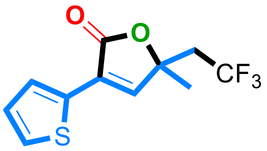

4o

^{19}F NMR (376 MHz, $CDCl_3$)

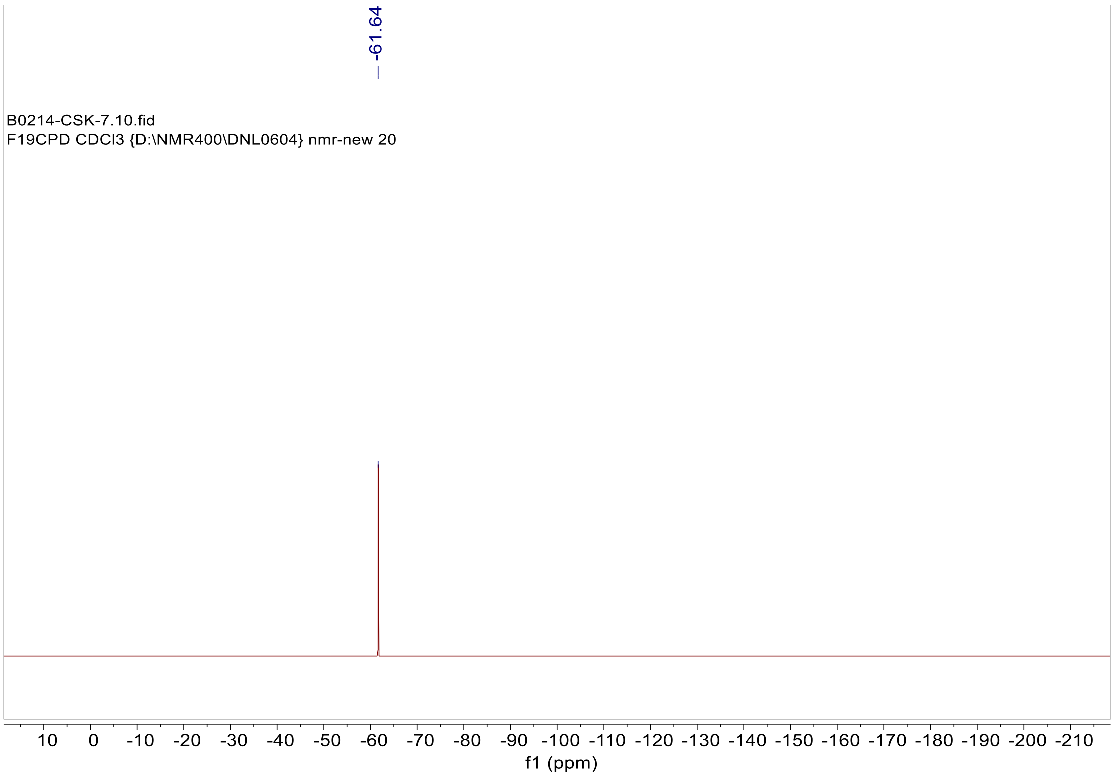

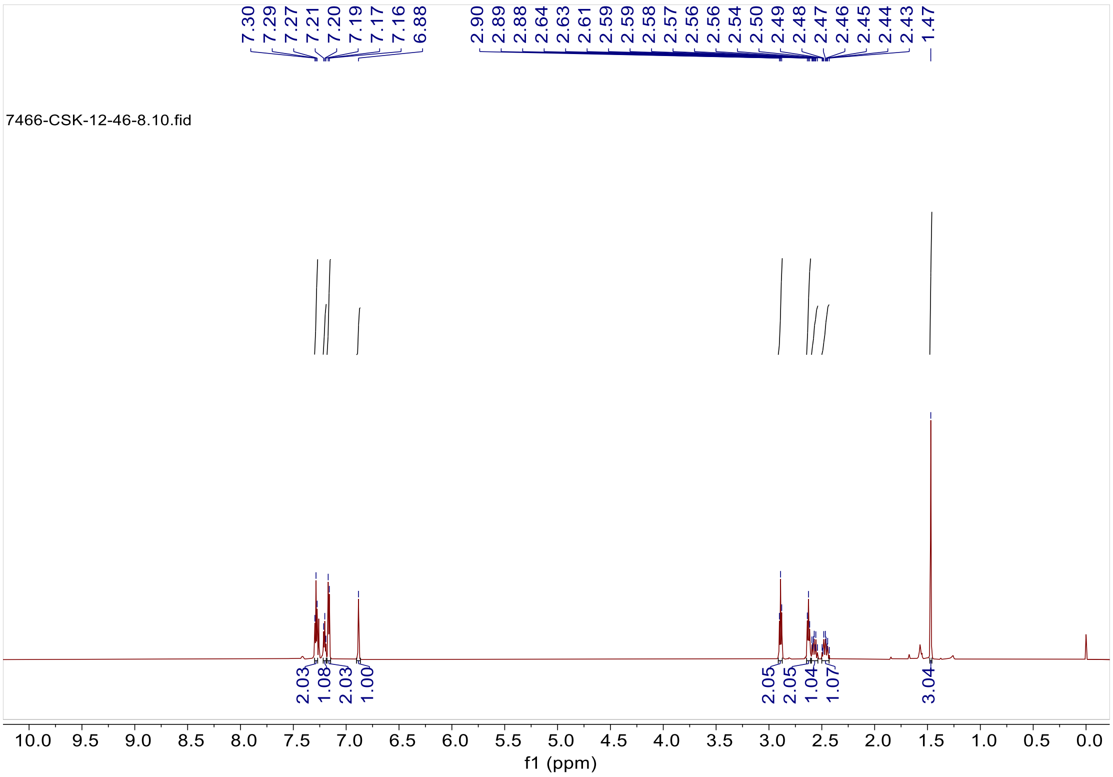

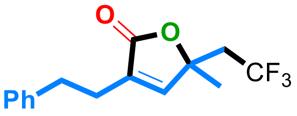

4p

^{1}H NMR (700 MHz, $CDCl_3$)

^{13}C NMR (176 MHz, $CDCl_3$)

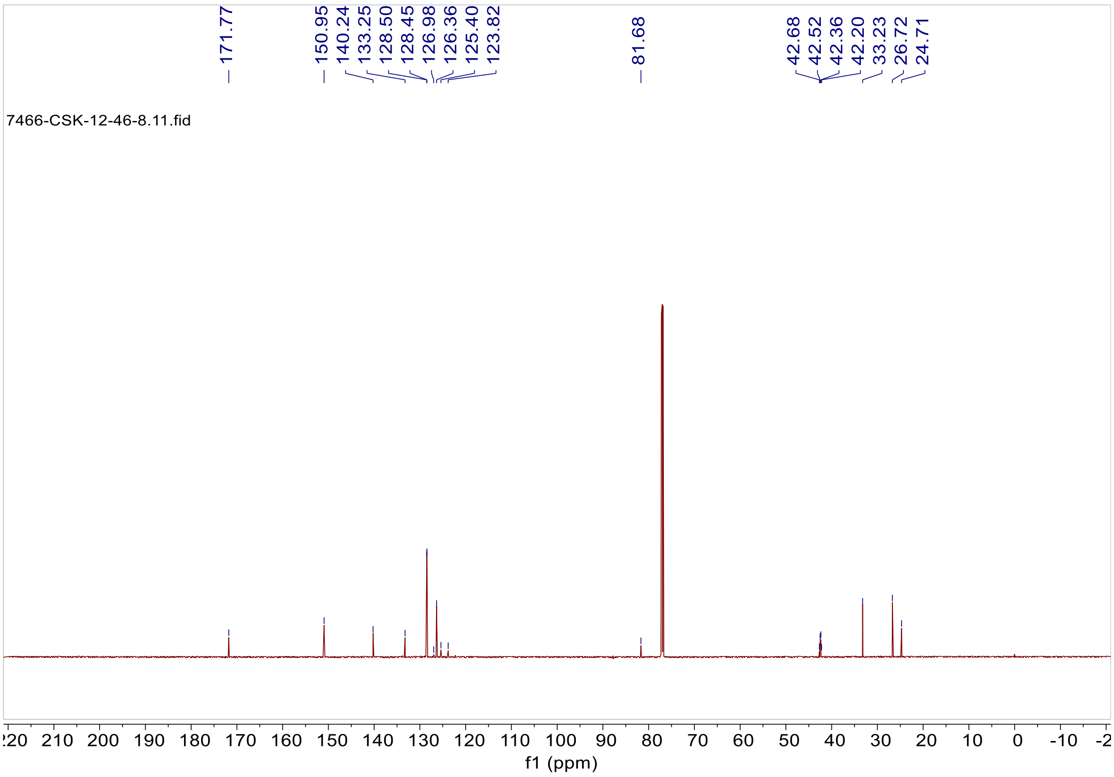

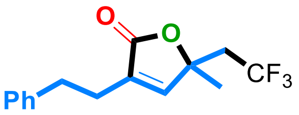

4p ^{19}F NMR (376 MHz, $CDCl_3$)

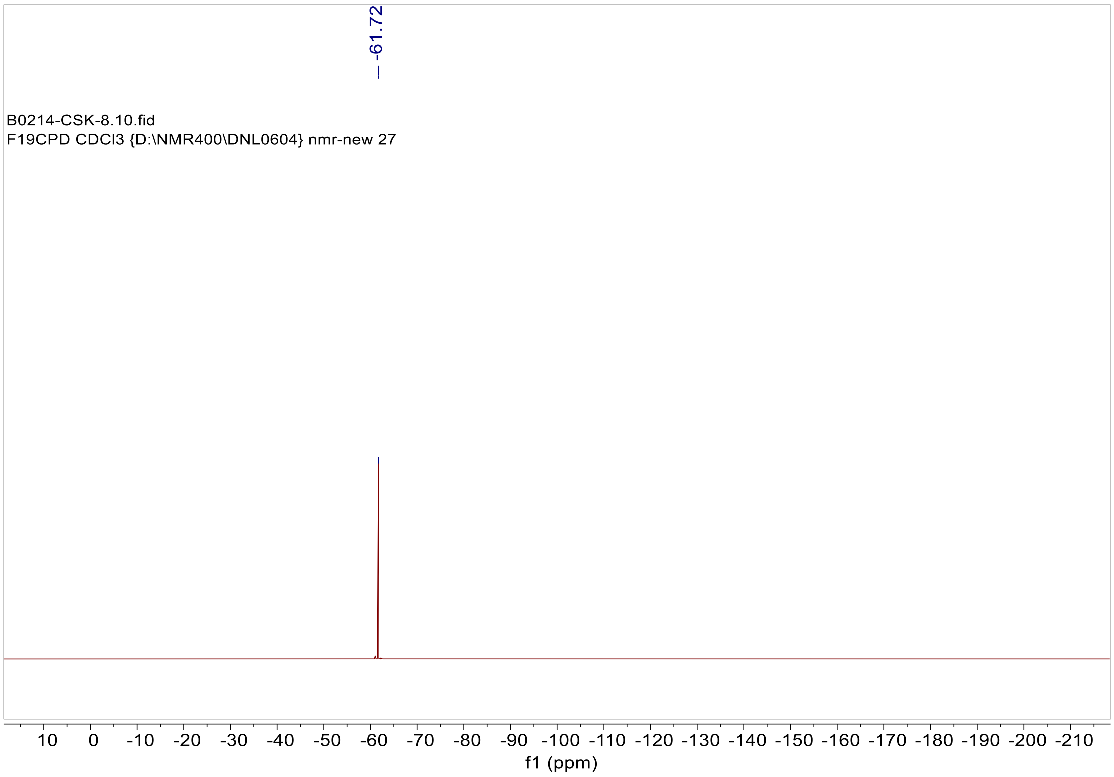

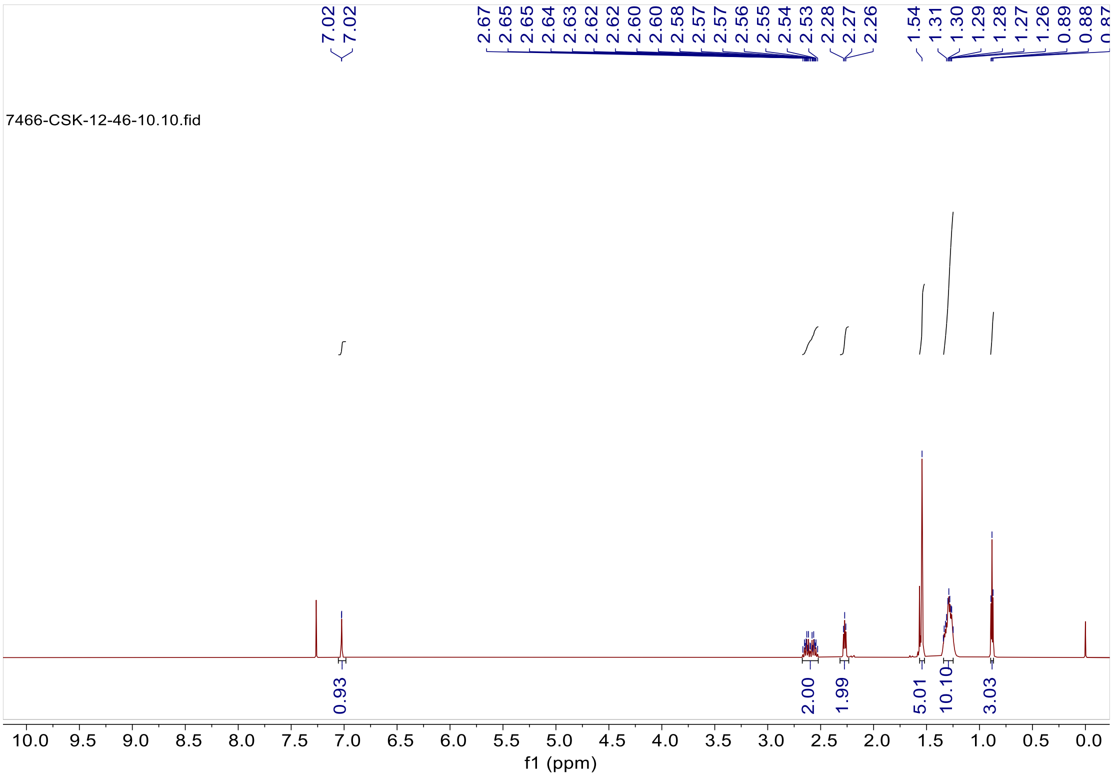

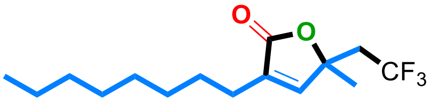

4q

^{1}H NMR (700 MHz, $CDCl_3$)
^{13}C NMR (176 MHz, $CDCl_3$)

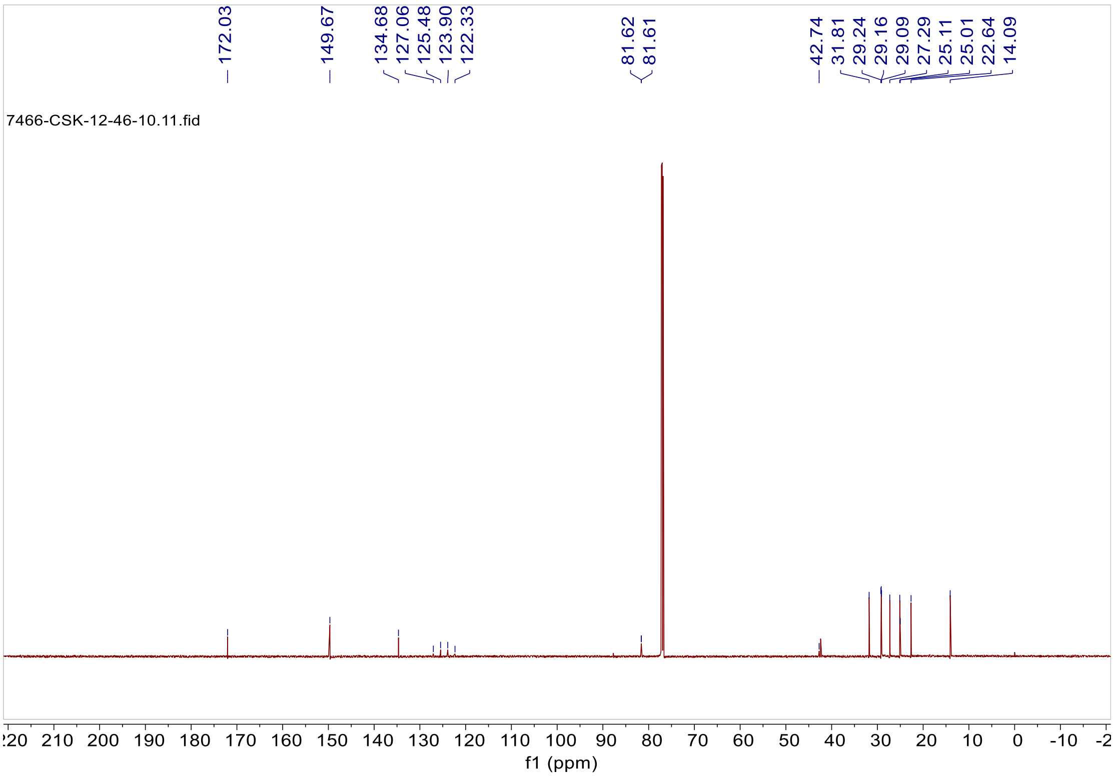

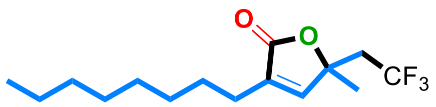

4q ^{19}F NMR (376 MHz, $CDCl_3$)

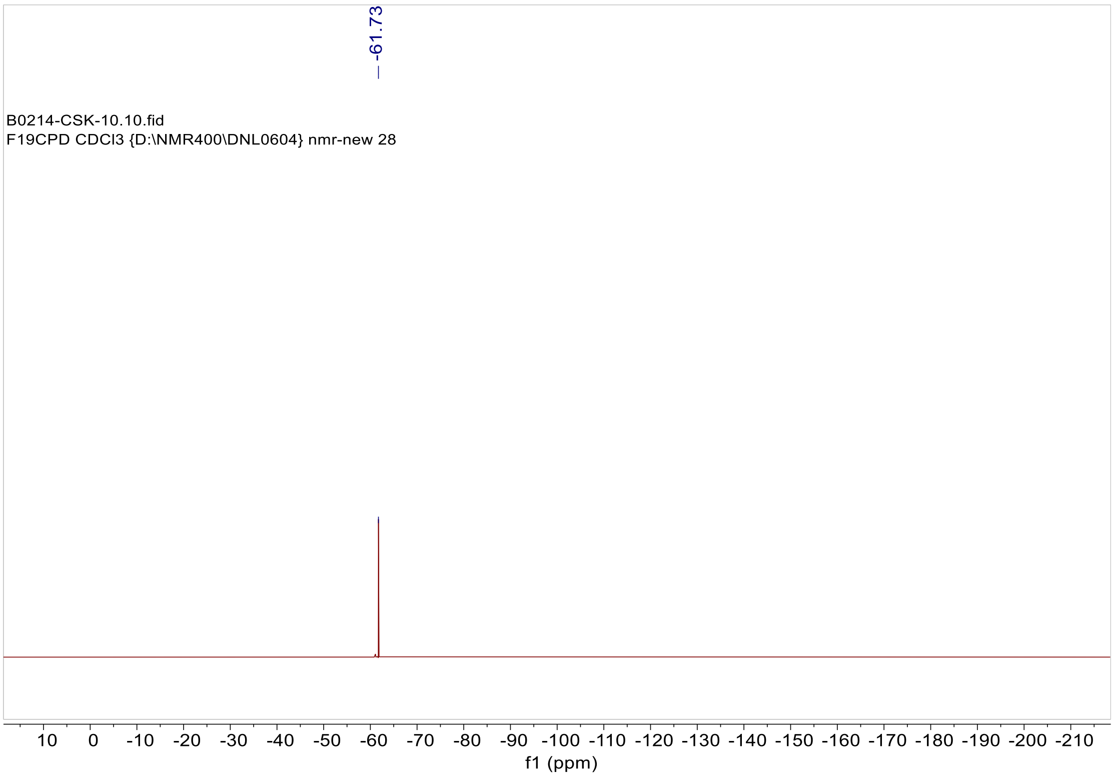

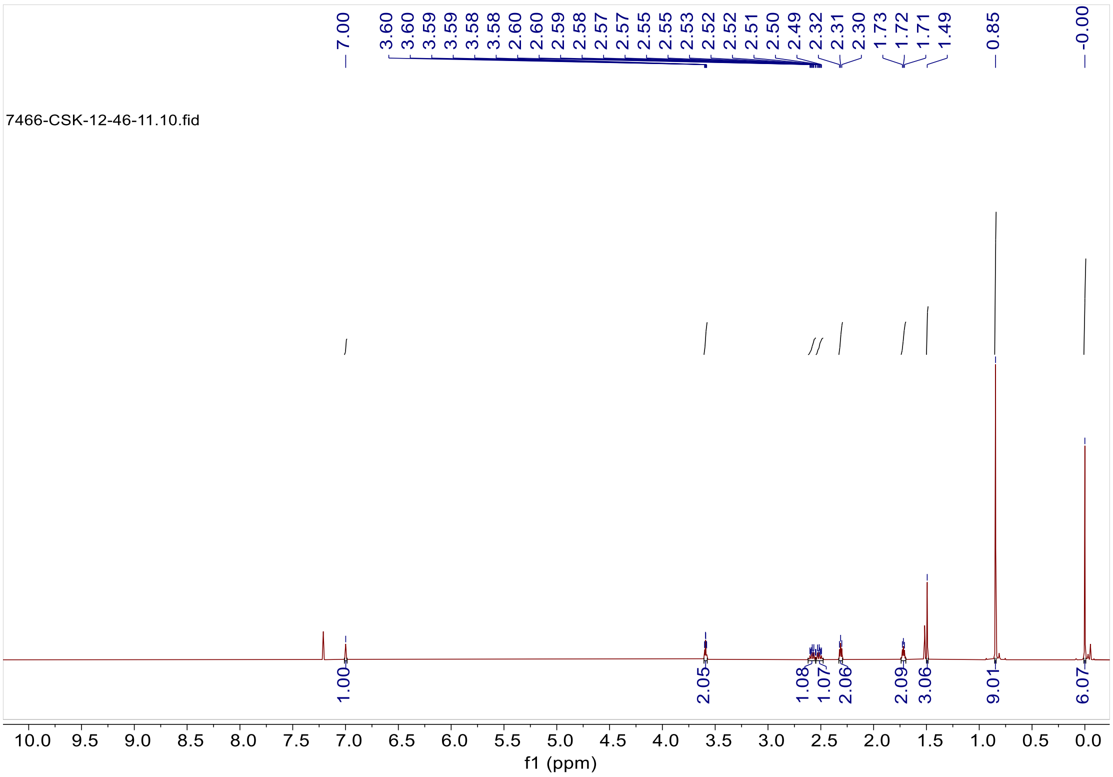

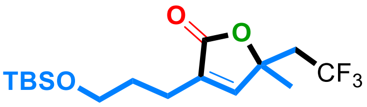

4r

^{1}H NMR (700 MHz, $CDCl_3$)
^{13}C NMR (176 MHz, $CDCl_3$)

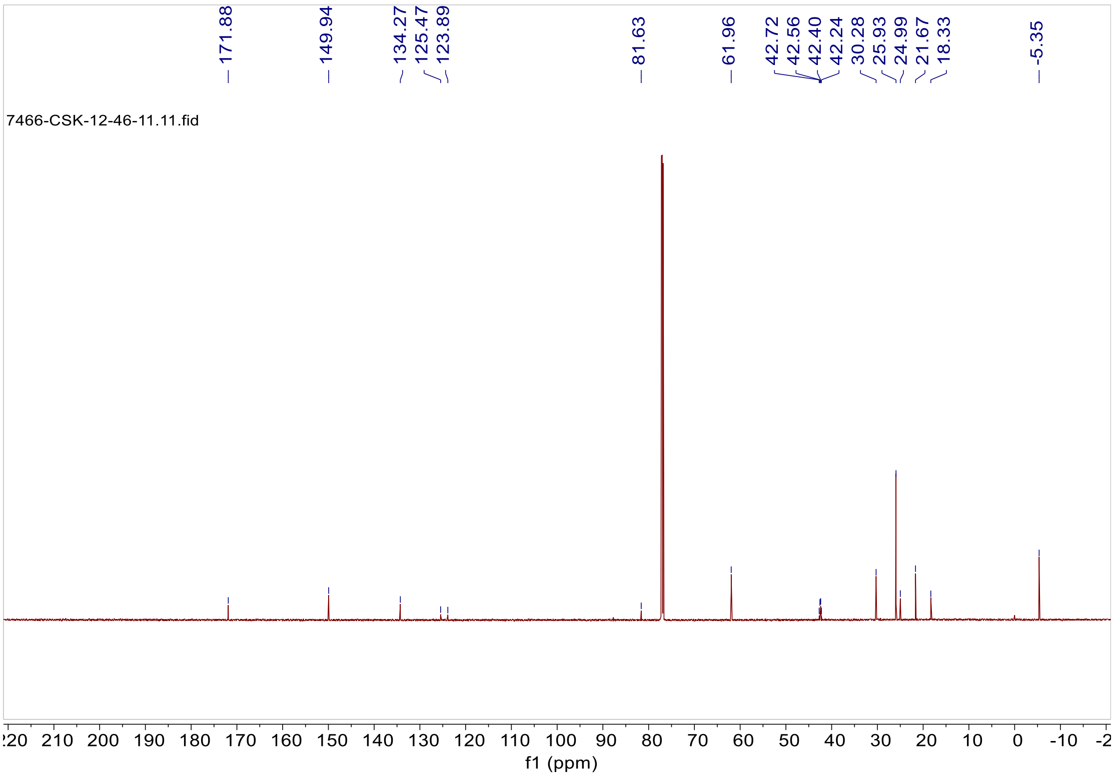

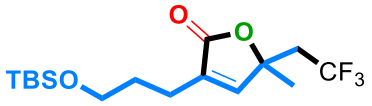

4r ^{19}F NMR (376 MHz, $CDCl_3$)

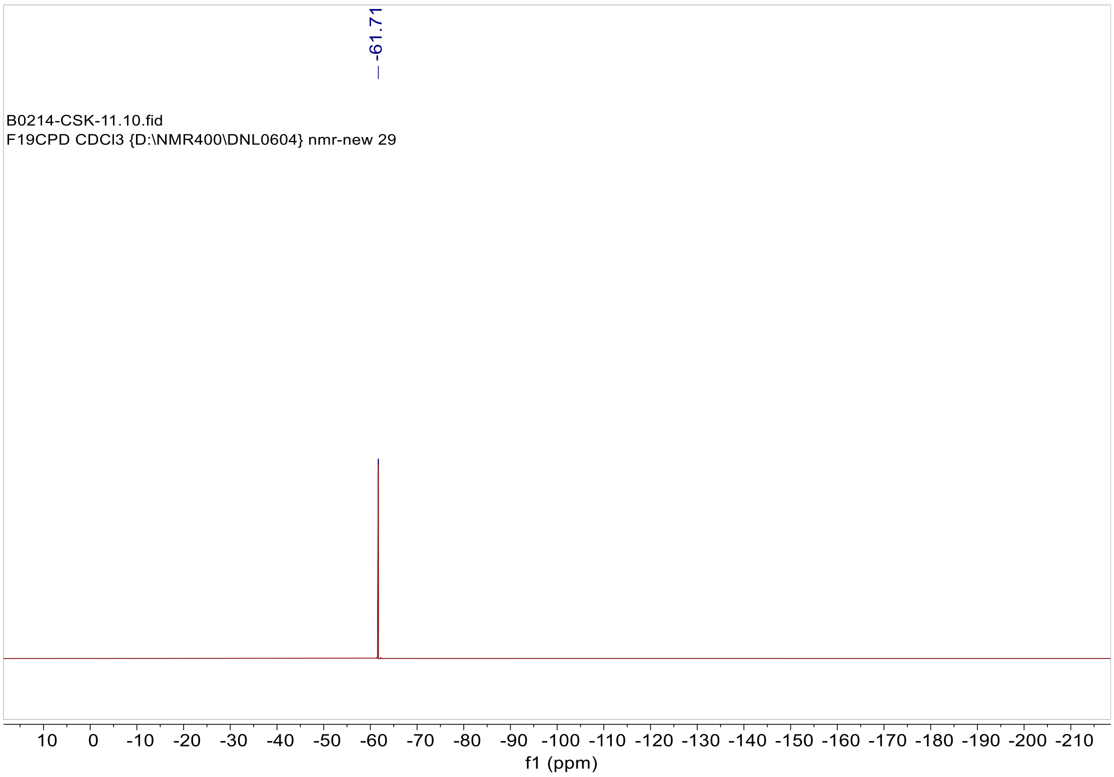

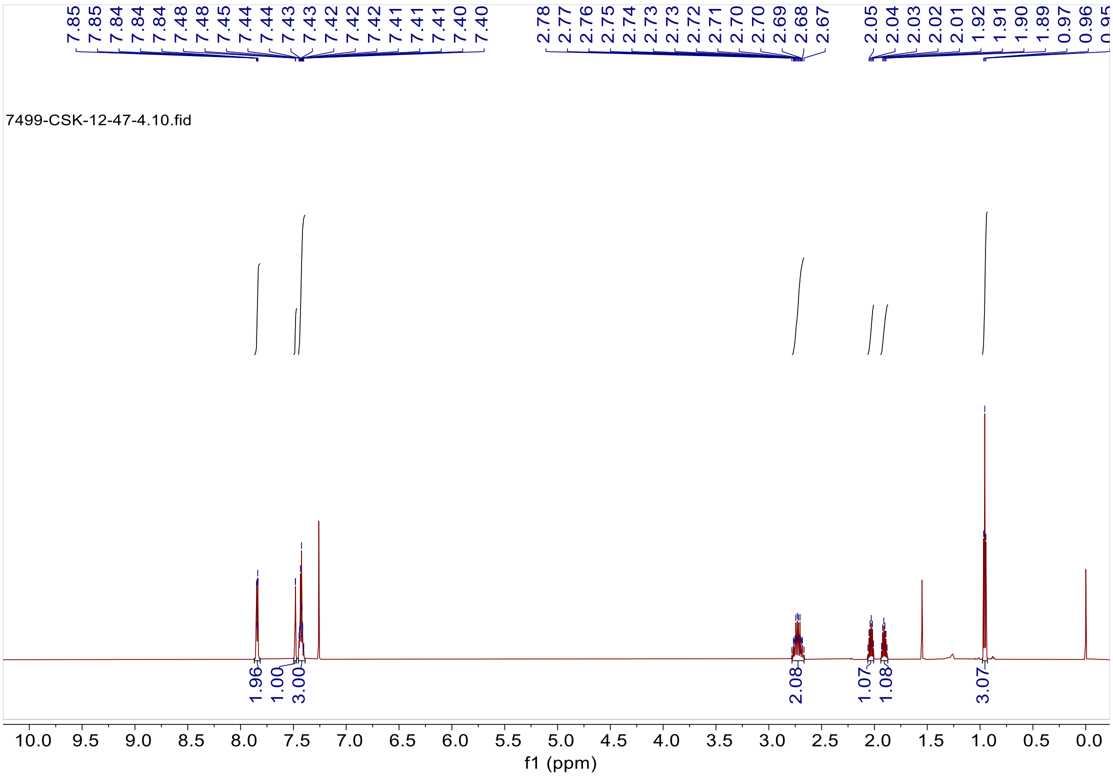

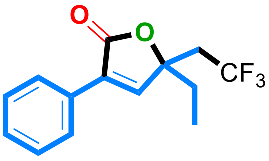

4s

^{1}H NMR (700 MHz, $CDCl_3$)

^{13}C NMR (176 MHz, $CDCl_3$)

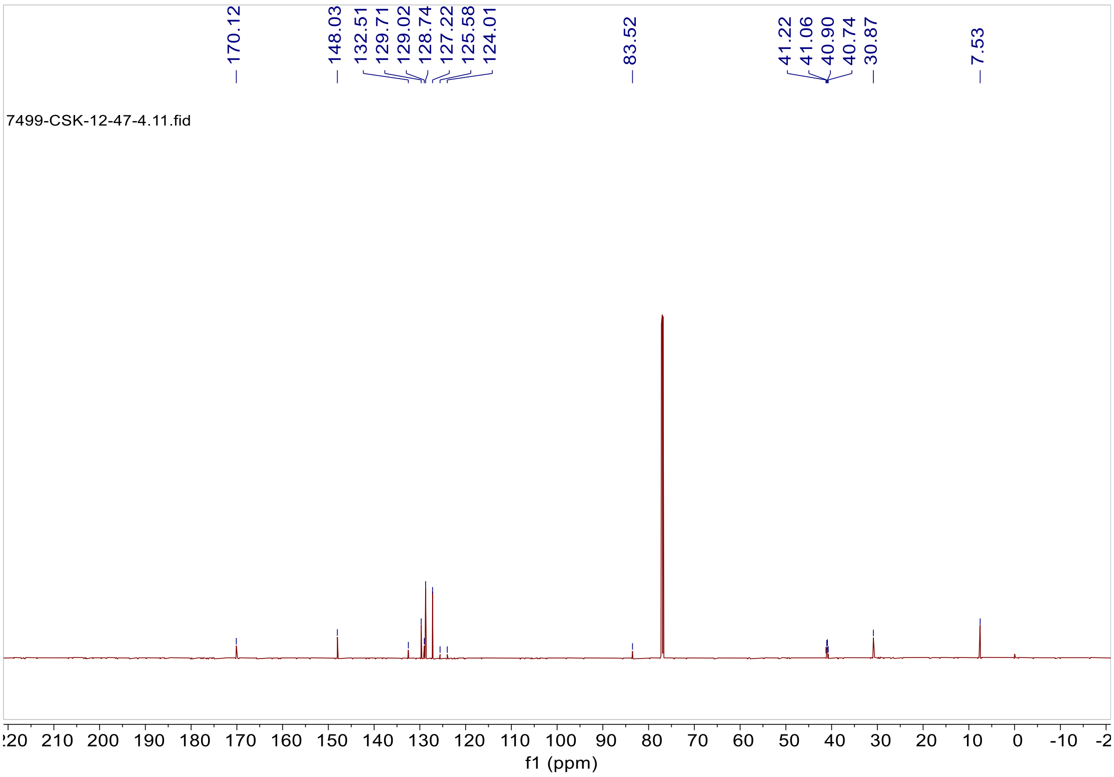

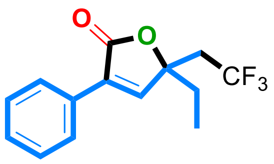

4s

^{19}F NMR (376 MHz, $CDCl_3$)

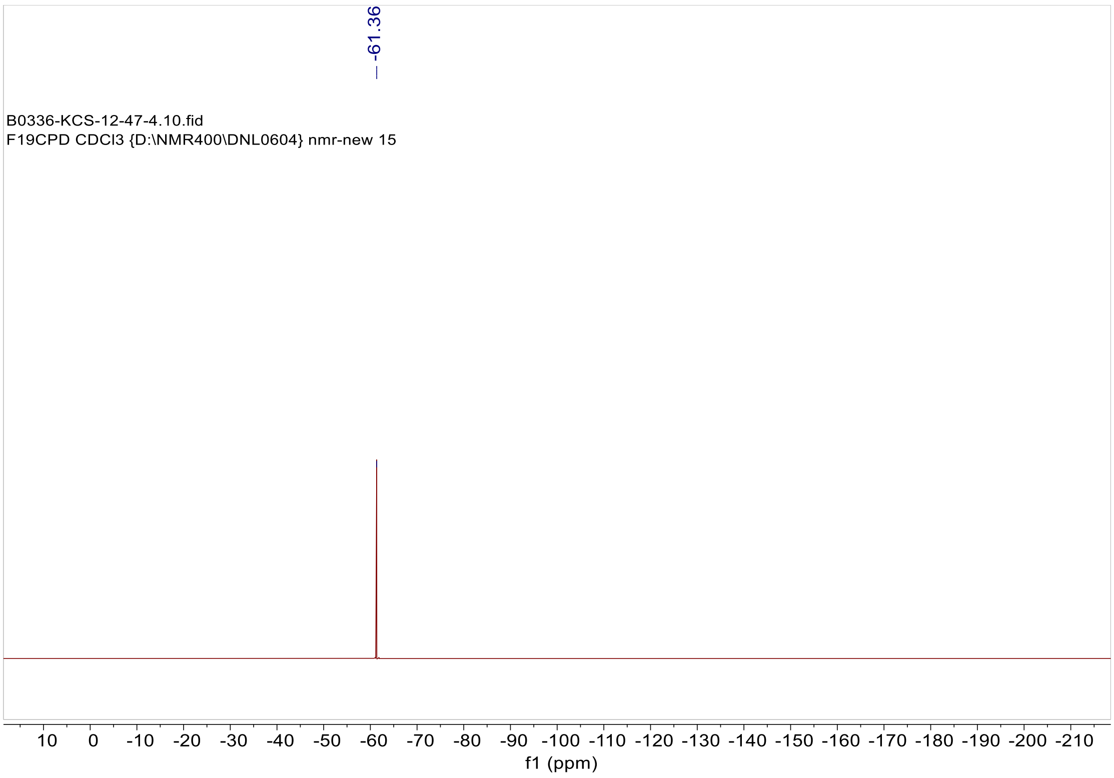

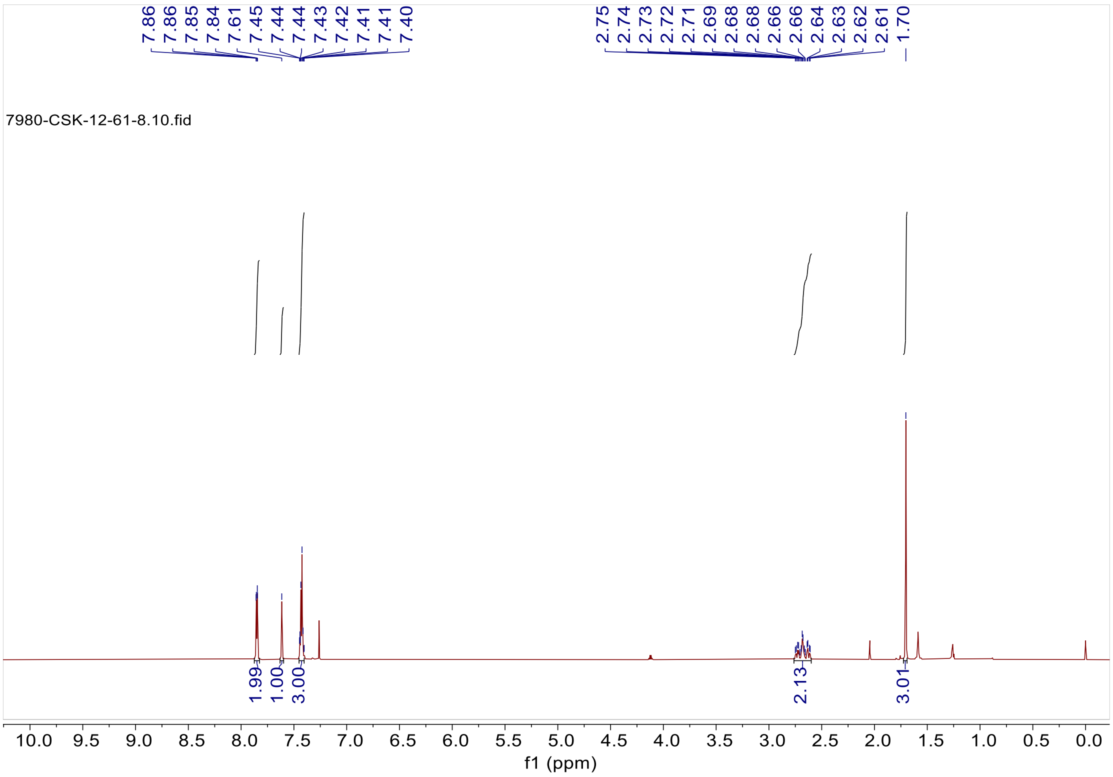

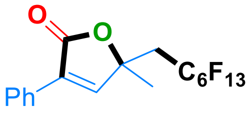

4u

^{1}H NMR (700 MHz, $CDCl_3$)

^{13}C NMR (176 MHz, $CDCl_3$)

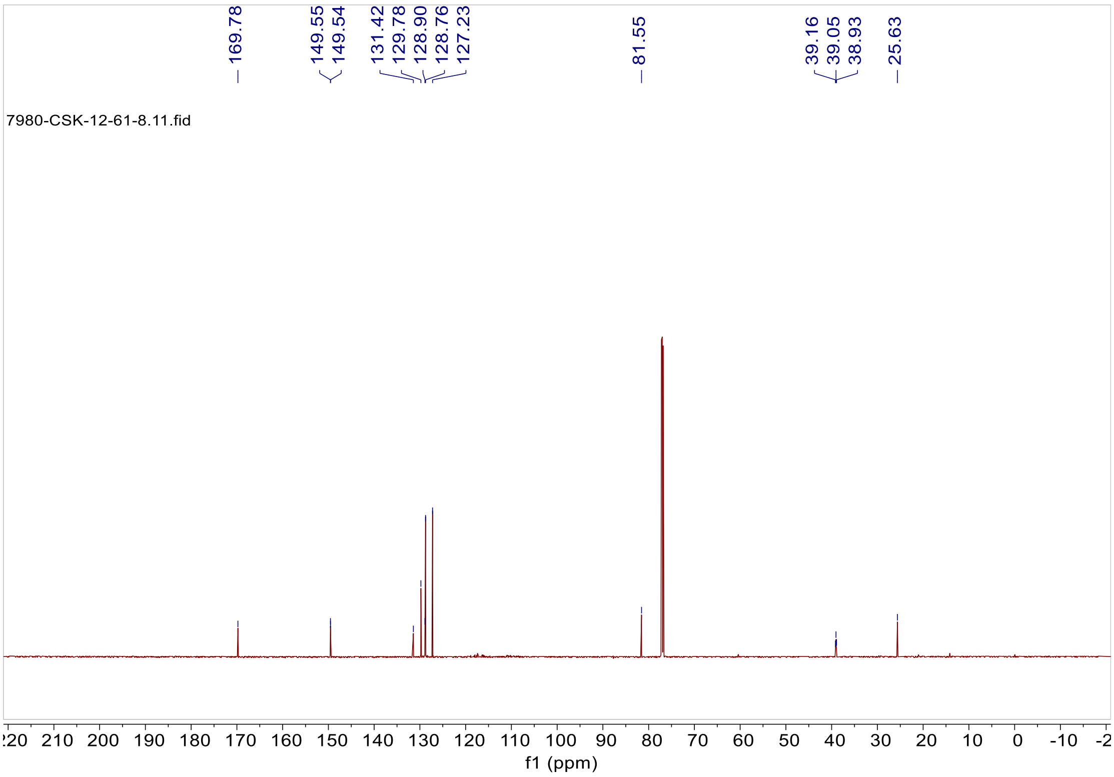

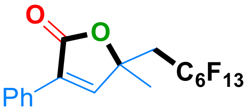

4u

^{19}F NMR (376 MHz, $CDCl_3$)

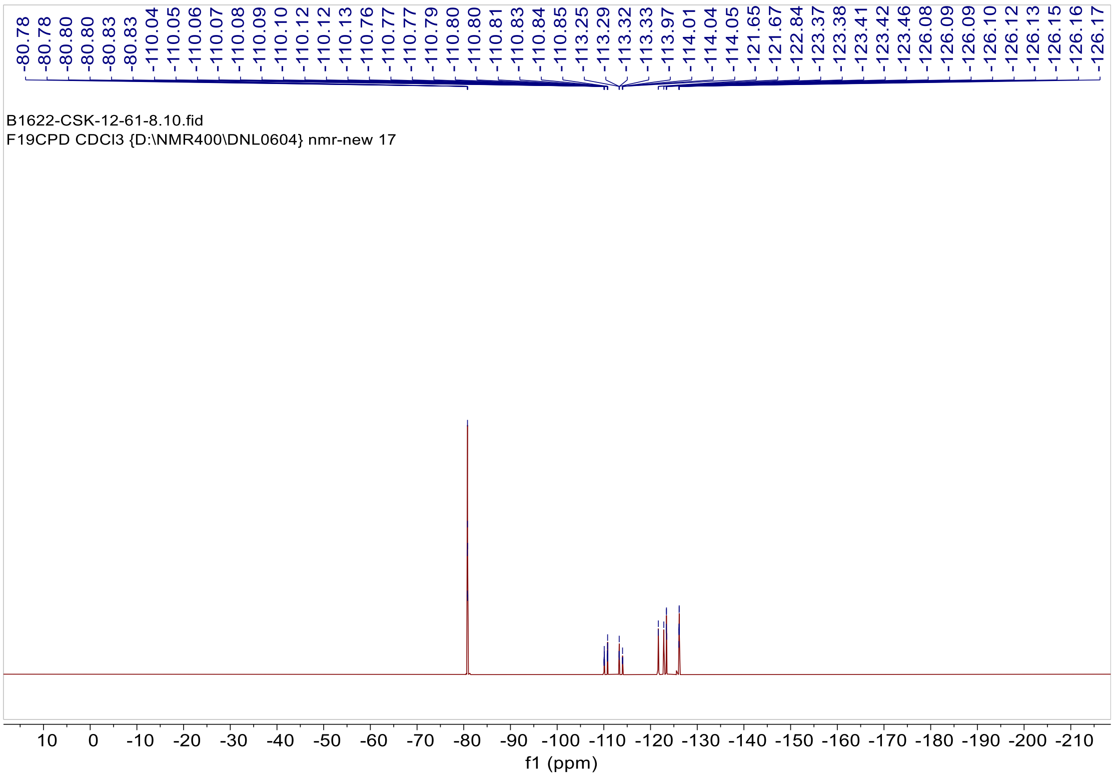

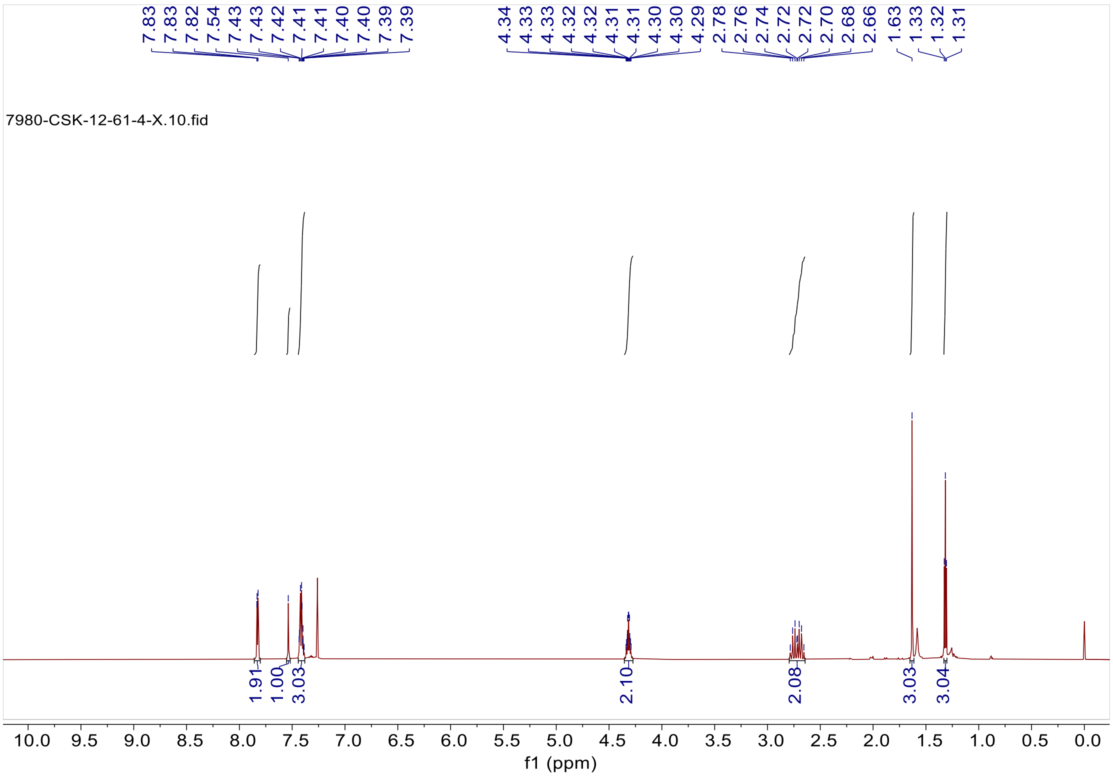

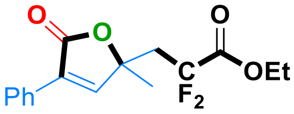

4v

^{1}H NMR (700 MHz, $CDCl_3$)
^{13}C NMR (176 MHz, $CDCl_3$)

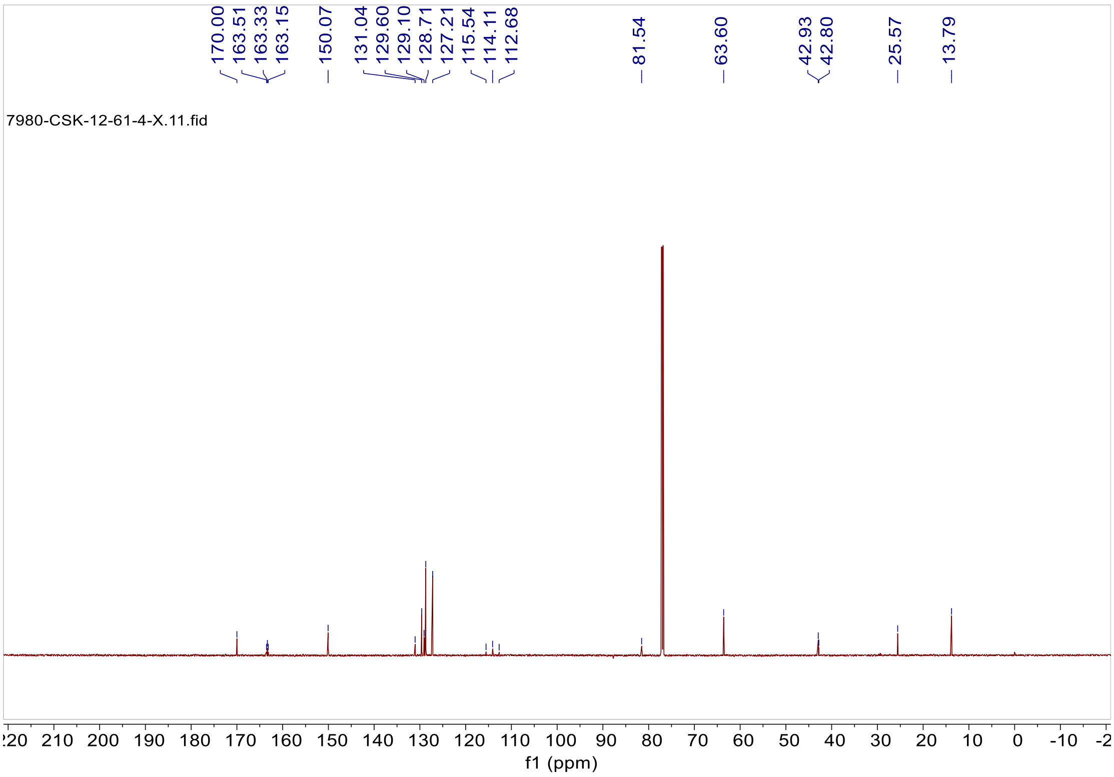

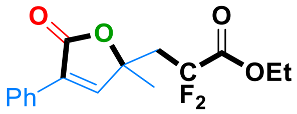

4v

^{19}F NMR (376 MHz, $CDCl_3$)

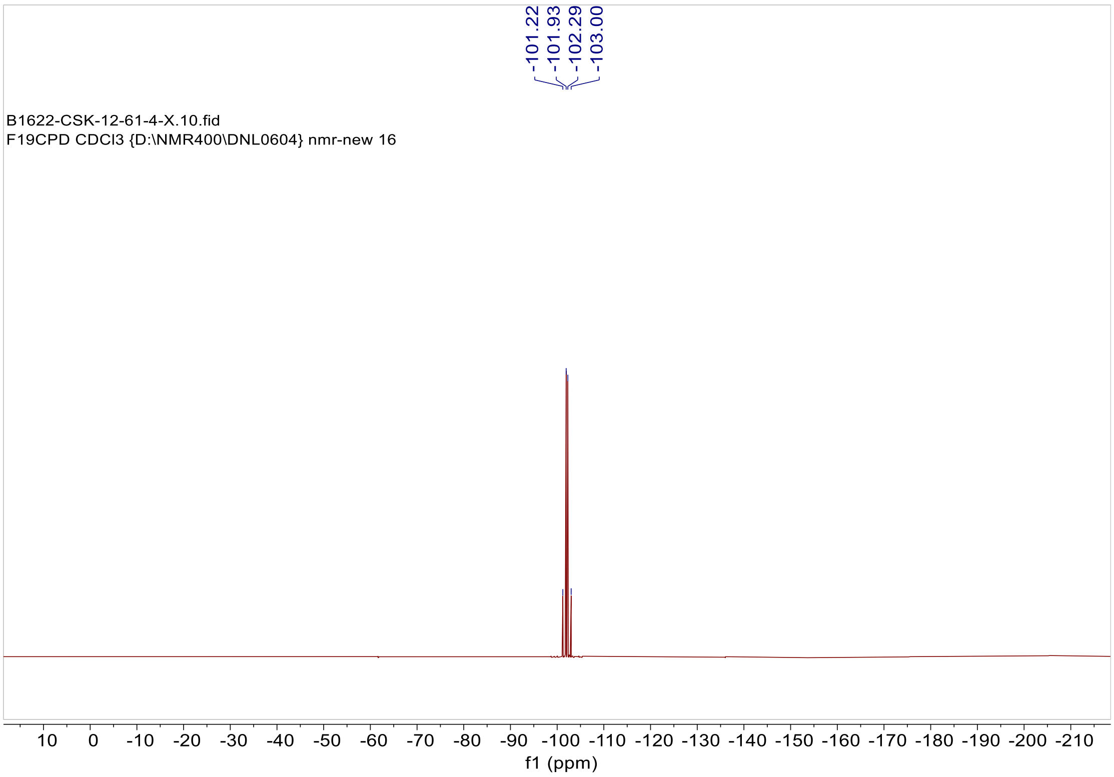

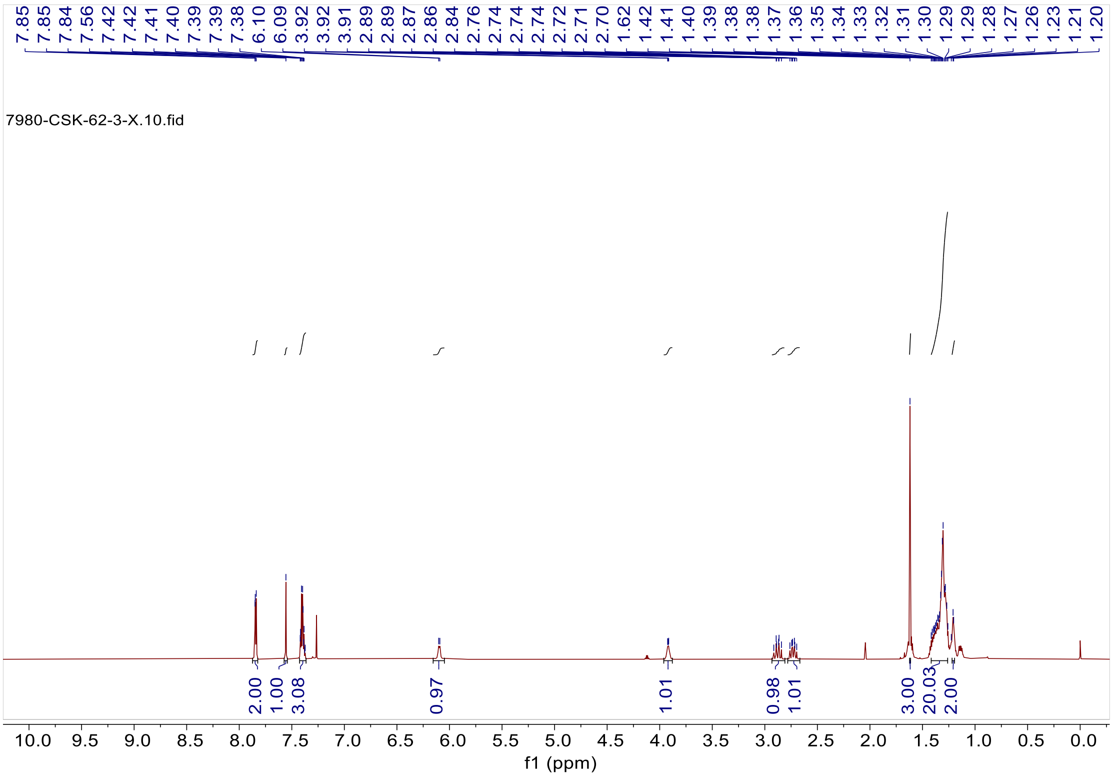

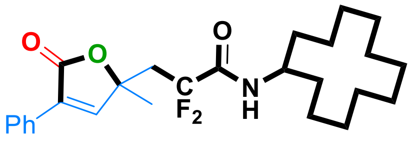

4w

^{1}H NMR (700 MHz, $CDCl_3$)
^{13}C NMR (176 MHz, $CDCl_3$)

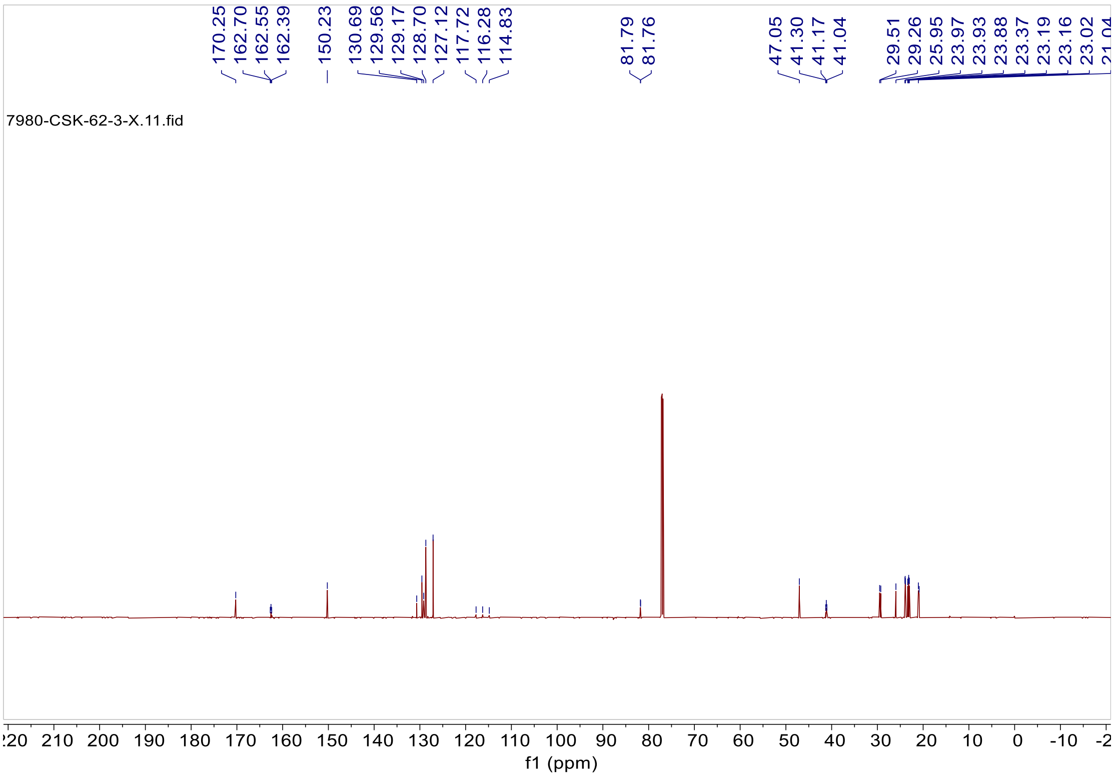

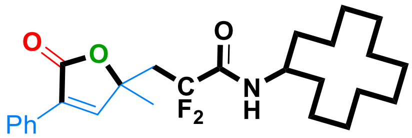

4w

^{19}F NMR (376 MHz, $CDCl_3$)

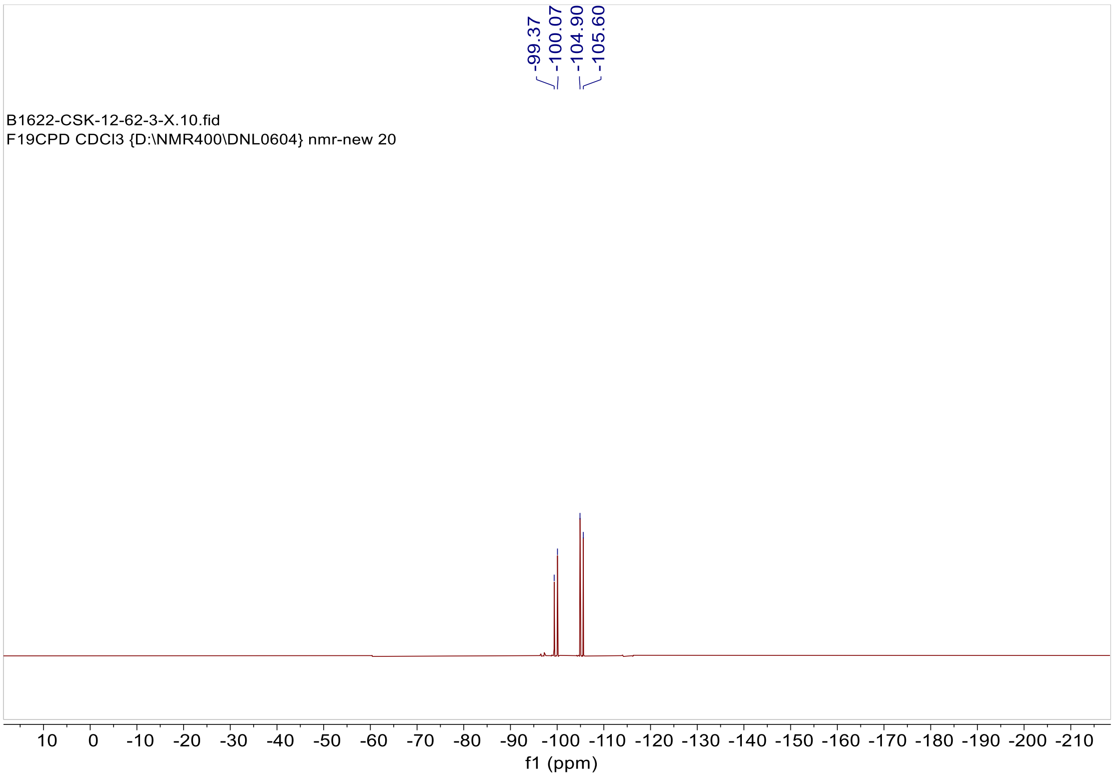

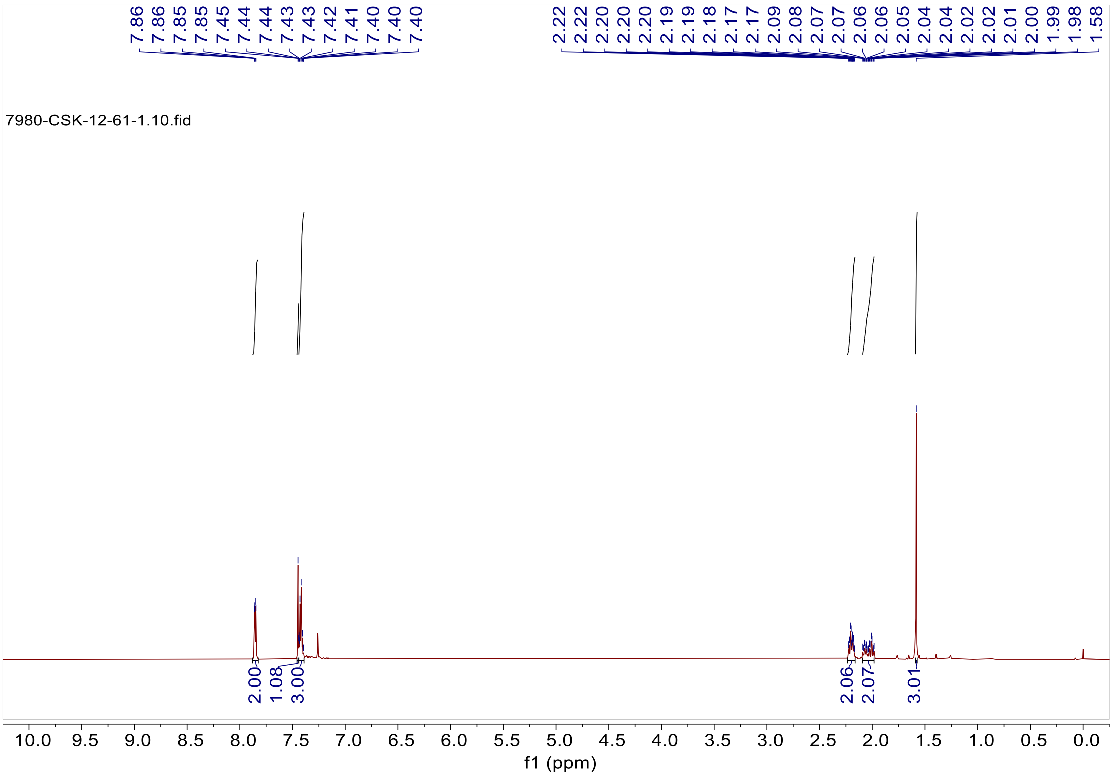

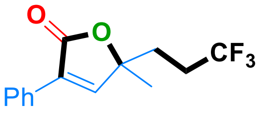

4x

^{1}H NMR (700 MHz, $CDCl_3$)

^{13}C NMR (176 MHz, $CDCl_3$)

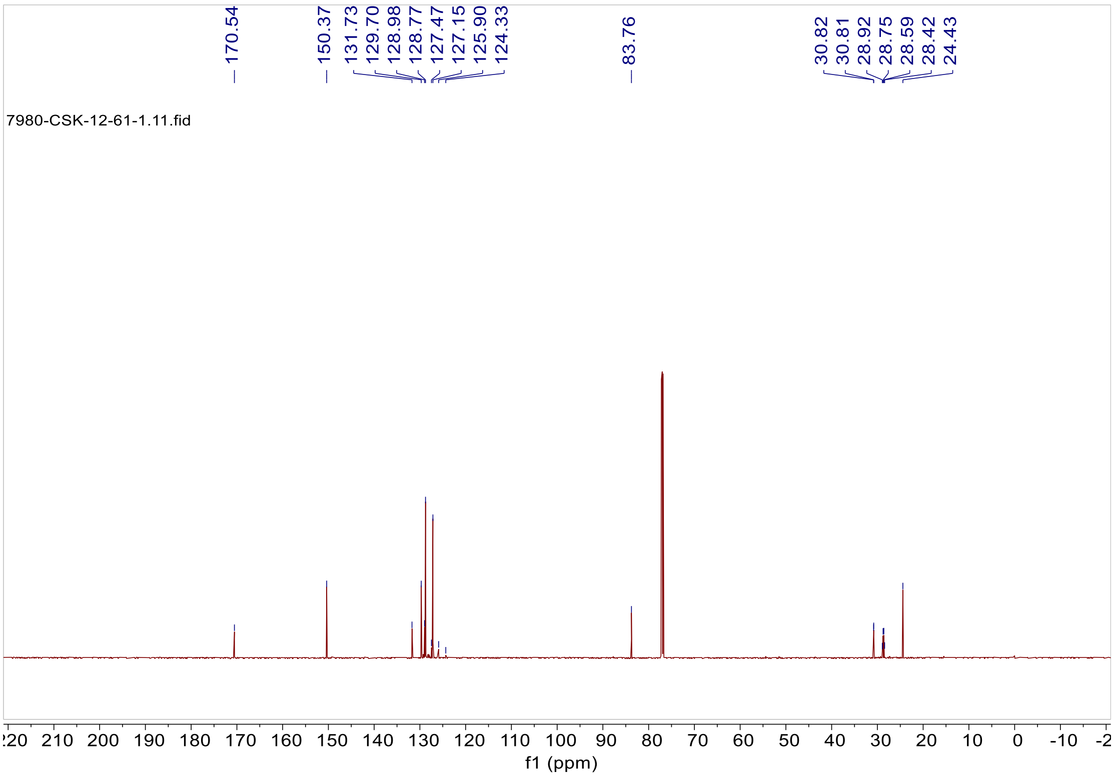

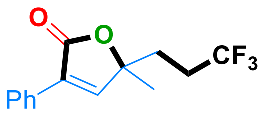

4x

^{19}F NMR (376 MHz, $CDCl_3$)

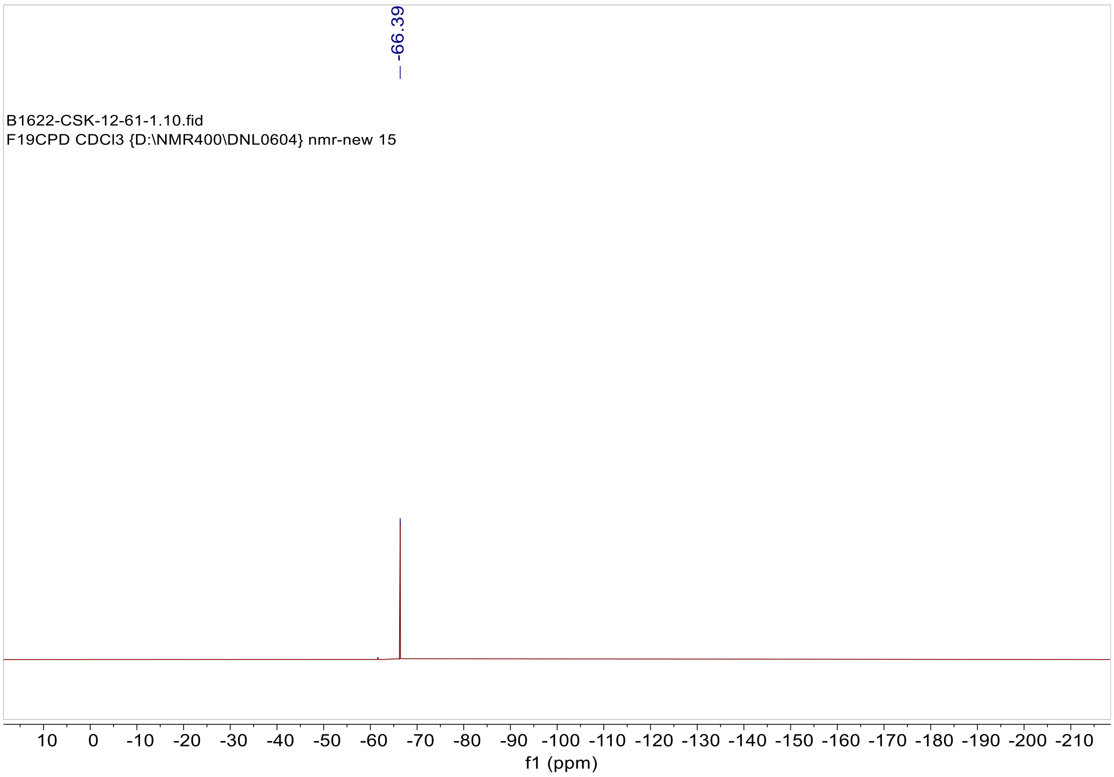

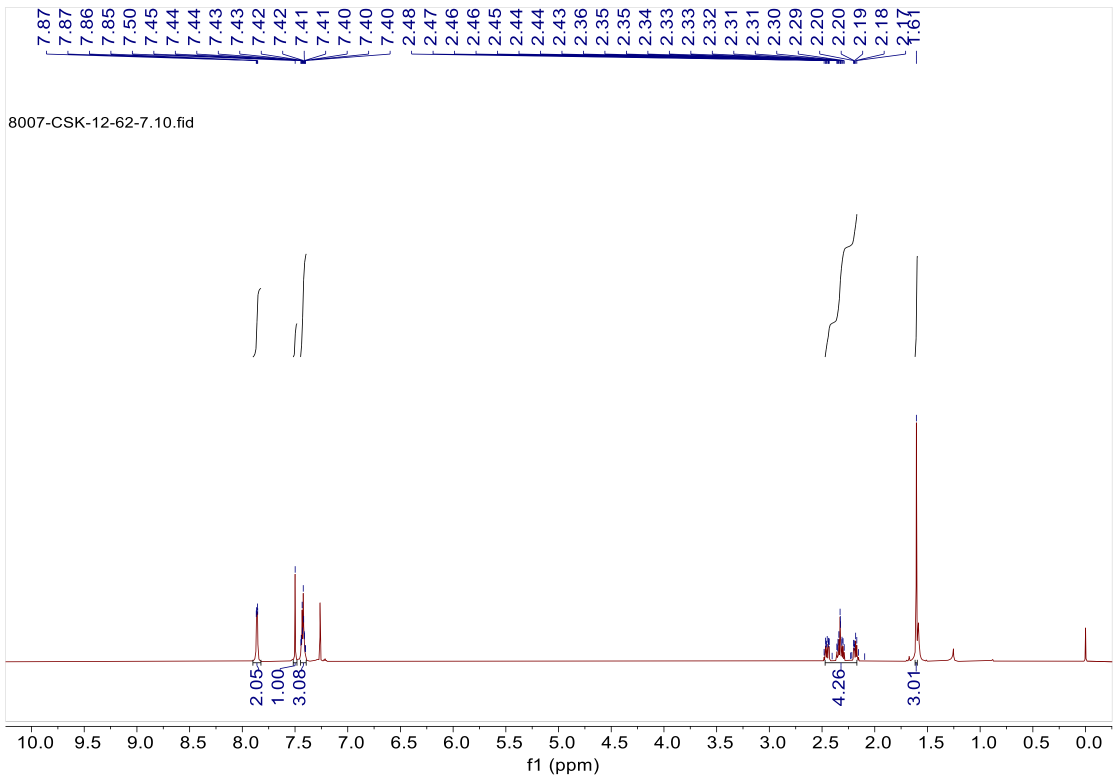

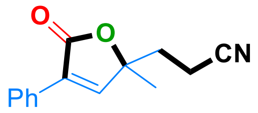

4y

^{1}H NMR (700 MHz, $CDCl_3$)
^{13}C NMR (176 MHz, $CDCl_3$)

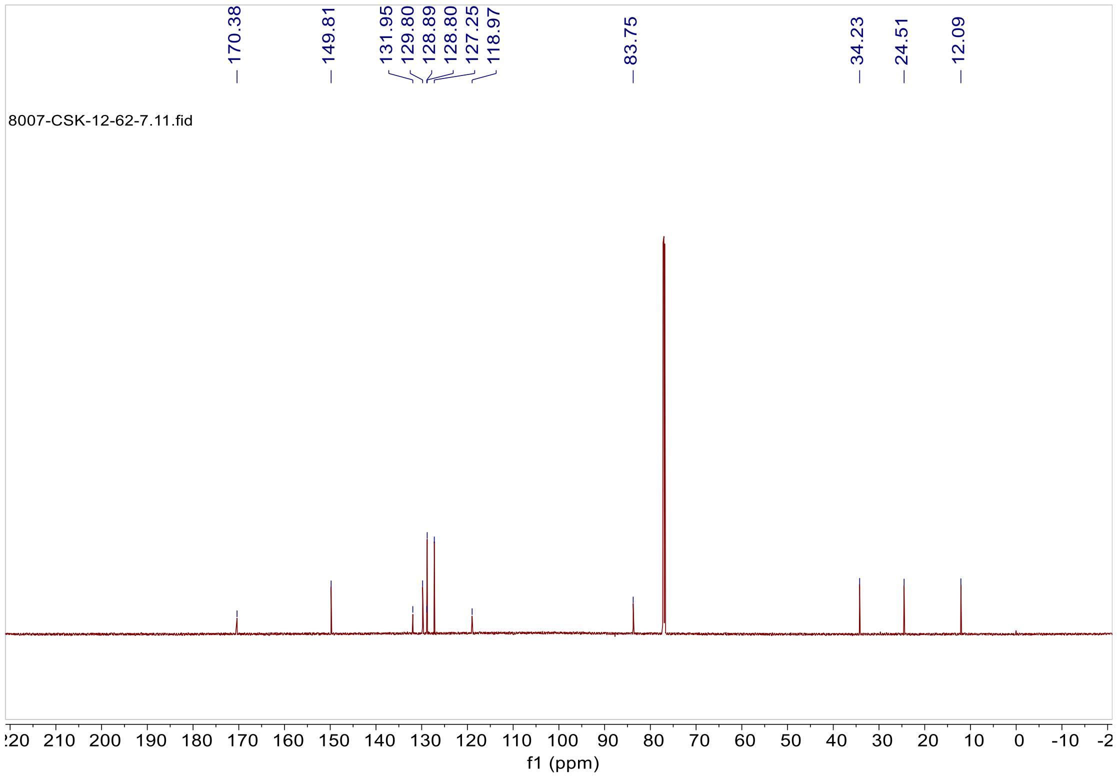

Supplement: Supplementary file 1 [file ol5c03004_si_001.pdf]
